# Inventory of the urban flora of Budapest (Hungary) highlighting new and noteworthy floristic records

**DOI:** 10.3897/BDJ.11.e110450

**Published:** 2023-11-27

**Authors:** Attila Rigó, Ákos Malatinszky, Zoltán Barina

**Affiliations:** 1 Doctoral School of Environmetnal Sciences, Hungarian University of Agriculture & Life Sciences, Páter Károly u. 1, 2100, Gödöllő, Hungary Doctoral School of Environmetnal Sciences, Hungarian University of Agriculture & Life Sciences, Páter Károly u. 1, 2100 Gödöllő Hungary; 2 Experimental Vegetation Ecology Research Group, Institute of Ecology and Botany, Centre for Ecological Research, Alkotmány út 4, 2163, Vácrátót, Hungary Experimental Vegetation Ecology Research Group, Institute of Ecology and Botany, Centre for Ecological Research, Alkotmány út 4, 2163 Vácrátót Hungary; 3 Institute for Wildlife Management and Nature Conservation, Hungarian University of Agriculture and Life Sciences, Páter Károly u. 1, 2100, Gödöllő, Hungary Institute for Wildlife Management and Nature Conservation, Hungarian University of Agriculture and Life Sciences, Páter Károly u. 1, 2100 Gödöllő Hungary; 4 H-1095, Ipar utca 3, Budapest, Hungary H-1095, Ipar utca 3 Budapest Hungary

**Keywords:** adventive, invasive, secondary habitats, occurrence records

## Abstract

**Background:**

The systematic urban floristic research of Budapest was started in 2018 by the authors with detailed methodology. One scope of the research was to gain knowledge on the plant taxa appearing in Budapest and to compile the inventory of the urban flora of Budapest.

**New information:**

We have provided the inventory of the urban flora of Budapest, which includes distribution data for all 973 taxa found in Budapest between May 2018 and May 2023. We also provided new detailed occurrence data for 49 species in Budapest. Seven of them are new to the adventive flora of Hungary (*Campanulaportenschlagiana* Roem. & Schult., *Clinopodiumnepeta* (L.) Kuntze, *Chasmanthiumlatifolium* (Michx.) H.O.Yates, *Cyrtomiumfortunei* J.Sm., *Linariamaroccana* Hook.f., *Talinumpaniculatum* (Jacq.) Gaertn.), three were rediscovered in Hungary (*Glebioniscoronaria* (L.) Cass. ex Spach, *Lagenariasiceraria* (Molina) Standl., *Sisymbriumirio* L.) and 18 were recorded for the first time in Budapest. We also provided data for two data-poor (*Artemisiascoparia* Waldst. & Kit., *Polygonumrurivagum* Jord. ex Boreau) species and we documented the major expansion of six species.

## Introduction

Scientific interest in urban ecological research, including urban floristic research, has increased in the past decades. This can be due to several reasons, one of which is the fact that urban areas are often invasion hot spots (Gaertner et al. 2017). Furthermore, spontaneously occurring plants (and other organisms) in cities pose an obvious risk to human health (Potgieter et al. 2017). In addition to these, cities can also be interesting from a taxonomic point of view, since recently more and more taxa are being described from urban environments, for example, *Stellariaruderalis* M. Lepší, P. Lepší, Z. Kaplan & P. Koutecký (Lepší et al. 2019). There were many examples for urban floristic research around the world (e.g. Tait et al. 2005, Zhao et al. 2009, Monalisa-Francisco and Ramos 2019 and Sogbossi et al. 2020). In the European context, many cities were also the subject of floristic surveys in recent decades. The most relevant of these are the floristic studies of Central European (e.g. Chocholoušková and Pyšek 2003, Feráková and Jarolímek 2010 and Jovanović et al. 2014) and Mediterranean cities (e.g. Celesti-Grapow et al. 2013, Stešević et al. 2014 and Salinitro et al. 2018). In addition, several comparative works were published on the flora of Europe's major cities (Pysek 1998, Lososová et al. 2011, La Sorte et al. 2014 and Kalusová et al. 2019). In Hungary, the floristic evaluation of Pécs (southern Hungary) was carried out by Wirth et al. (2020c) and Wirth et al. 2020d.

The research of the flora of Budapest has a long history. Sadler (1840) provided the complete flora of Pest-Pilis-Solt County of historical Hungary, where scattered data were provided on the built-up urban areas of Budapest. Borbás (1879) specifically provided the floristic evaluation of Budapest and its surroundings. In the 20^th^ century, mainly descriptive and educational works were published about the flora of Budapest (Pénzes 1942, Pénzes and Csízy 1956 and Zólyomi 1958). At the end of the century, however, an inventory of Budapest's flowering plants was published (Hegedűs 1994). Most recently, Somlyay (2011), Somlyay et al. (2016) and Somlyay and Csábi (2019) researched the natural vegetation cover of Budapest and its surroundings. Rigó and Barina (2020) started the systematic, habitat-based floristic research of the urban built-up areas of Budapest in 2018.

## Materials and methods

### Territorial delimitation

The research area was Budapest, the capital of Hungary. The city is located in Central Hungary, between northern latitude 47.613437° and 47.349269° and eastern longitude 18.926381° and 19.334538°. The size of the city is about 525 km^2^, of which 388 km^2^ is the built-up interior area (Hungarian Central Statistical Office 2023). The altitude of the city varies from 95 m to 527 m. Budapest is crossed by the River Danube and surrounded by the Buda Mountains and the Pest plains. Budapest is divided into 23 administrative districts, six of which form the city core, nine districts are the inner districts surrounding the city core and the rest are the outer districts. In 2018, the city had approx. 1.75 million inhabitants (Hungarian Central Statistical Office 2023). The study was restricted to the secondary habitats of the urban built-up areas of Budapest, natural and semi-natural habitats were not included in the research.

### Floristic procedures

Within the territorial units, we prepared a complete list of species for each of the distinguishable urban (micro-)habitats (Rigó and Barina 2020). We covered 1,613 street sections and squares in all districts of Budapest and registered a taxon list in each of them. As a result, we compiled an inventory of the urban flora of Budapest. The inventory is solely based on our recent data (2018-2023). We included the species name, residence status and distribution (in which districts of Budapest it occurred) for each species.

In the case of new, rare and noteworthy findings, we recorded and added the following data: scientific name, family, continent, country, county, municipality (= district), locality (= name of the public place), decimal latitude, decimal longitude, event date (= date of observation), habitat, individual count (= approximate number of the specimens), reproductive condition, recorder, identifier, occurrenceID. We also added notes on the origin, distribution, residence time and invasion potential, if relevant.

Nomenclature and classification follows World Flora Online (2023). We used Csiky et al. (2023) to determine the residence time of alien taxa.

## Data resources

We used data from Suppl. material 1 for listing new and noteworthy floristic data in the 'Taxon Treatment' section and used Suppl. materials 2, 3 for the inventory of the secondary habitats of the urban, built-up areas of Budapest.

## Taxon treatments

### Achillea filipendulina

Lam. 1783

01171DE8-4C7C-596C-89AC-E3D4293A82FD

#### Materials

- **Type status:** Other material. **Occurrence:** recordedBy: Attila Rigó; individualCount: 20; reproductiveCondition: non-reproductive; occurrenceID: 1E05CFC0-22FF-5306-8C50-639A9EFDDE79; **Taxon:** scientificName: Achilleafilipendulina; family: Asteraceae; **Location:** continent: Europe; country: Hungary; county: Budapest; municipality: Budapest I.; locality: Hunyadi János road; decimalLatitude: 47.500629; decimalLongitude: 19.036963; **Identification:** identifiedBy: Attila Rigó; **Event:** eventDate: 29/04/2022; habitat: flower bed
- **Type status:** Other material. **Occurrence:** recordedBy: Attila Rigó; individualCount: 3; reproductiveCondition: non-reproductive; occurrenceID: DCD6088C-CE2B-5BD0-A4E7-F021E9C2EAE9; **Taxon:** scientificName: Achilleafilipendulina; family: Asteraceae; **Location:** continent: Europe; country: Hungary; county: Budapest; municipality: Budapest IX.; locality: Közraktár street; decimalLatitude: 47.481188; decimalLongitude: 19.065478; **Identification:** identifiedBy: Attila Rigó; **Event:** eventDate: 23/05/2022; habitat: crevices of pavement
- **Type status:** Other material. **Occurrence:** recordedBy: Attila Rigó; individualCount: 15; reproductiveCondition: non-reproductive; occurrenceID: 5E505BAF-5C43-52BF-BCAE-7F8E6F3489C6; **Taxon:** scientificName: Achilleafilipendulina; family: Asteraceae; taxonRank: species; **Location:** continent: Europe; country: Hungary; county: Budapest; municipality: Budapest IX.; locality: Nehru park; decimalLatitude: 47.480913; decimalLongitude: 19.064301; **Identification:** identifiedBy: Attila Rigó; **Event:** eventDate: 23/05/2022; habitat: flower beds and crevices of pavement
- **Type status:** Other material. **Occurrence:** recordedBy: Attila Rigó; individualCount: 2; reproductiveCondition: in bloom; occurrenceID: B195FC41-F6B6-520A-9B99-98FA2298958F; **Taxon:** scientificName: Achilleafilipendulina; family: Asteraceae; taxonRank: species; **Location:** continent: Europe; country: Hungary; county: Budapest; municipality: Budapest VIII.; locality: Corvin alley; decimalLatitude: 47.486356; decimalLongitude: 19.071072; **Identification:** identifiedBy: Attila Rigó; **Event:** eventDate: 19/06/2022; habitat: crevices of pavement
- **Type status:** Other material. **Occurrence:** recordedBy: Attila Rigó; individualCount: 20; reproductiveCondition: non-reproductive; occurrenceID: BDD44849-A1A5-5240-AAFF-4CD702CDEC17; **Taxon:** scientificName: Achilleafilipendulina; family: Asteraceae; taxonRank: species; **Location:** continent: Europe; country: Hungary; county: Budapest; municipality: Budapest II.; locality: Millenáris park; decimalLatitude: 47.511024; decimalLongitude: 19.025587; **Identification:** identifiedBy: Attila Rigó; **Event:** eventDate: 06/07/2022; habitat: crevices of pavement
- **Type status:** Other material. **Occurrence:** recordedBy: Attila Rigó; individualCount: 3; reproductiveCondition: fruit-bearing; occurrenceID: 0CFF4A48-21EE-5D9D-8A57-E4001E3F154A; **Taxon:** scientificName: Achilleafilipendulina; family: Asteraceae; taxonRank: species; **Location:** continent: Europe; country: Hungary; county: Budapest; municipality: Budapest XIV.; locality: Keszeg street; decimalLatitude: 47.499807; decimalLongitude: 19.282639; **Identification:** identifiedBy: Attila Rigó; **Event:** eventDate: 24/07/2022; habitat: crevices of pavement
- **Type status:** Other material. **Occurrence:** recordedBy: Attila Rigó; individualCount: 5; reproductiveCondition: non-reproductive; occurrenceID: B67E5638-0CAF-599C-9FFB-56037BEF59C2; **Taxon:** scientificName: Achilleafilipendulina; family: Asteraceae; taxonRank: species; **Location:** continent: Europe; country: Hungary; county: Budapest; municipality: Budapest XIII.; locality: Schulek Frigyes promenade; decimalLatitude: 47.535881; decimalLongitude: 19.052308; **Identification:** identifiedBy: Attila Rigó; **Event:** eventDate: 27/10/2022; habitat: crevices of pavement
- **Type status:** Other material. **Occurrence:** recordedBy: Attila Rigó; individualCount: 6; reproductiveCondition: non-reproductive; occurrenceID: C96F3579-0DBF-5469-8A2F-504183EF6D21; **Taxon:** scientificName: Achilleafilipendulina; family: Asteraceae; taxonRank: species; **Location:** continent: Europe; country: Hungary; county: Budapest; municipality: Budapest IX.; locality: Angyal István park; decimalLatitude: 47.485695; decimalLongitude: 19.069504; **Identification:** identifiedBy: Attila Rigó; **Event:** eventDate: 24/11/2022; habitat: crevices of pavement
- **Type status:** Other material. **Occurrence:** recordedBy: Attila Rigó, Ákos Malatinszky; individualCount: 30; reproductiveCondition: in bloom; occurrenceID: 28582A6E-F40B-5B61-A52E-D52D39829182; **Taxon:** scientificName: Achilleafilipendulina; family: Asteraceae; taxonRank: species; **Location:** continent: Europe; country: Hungary; county: Budapest; municipality: Budapest IX.; locality: Nádasdy street; decimalLatitude: 47.470380; decimalLongitude: 19.080150; **Identification:** identifiedBy: Attila Rigó; **Event:** eventDate: 08/10/2021; habitat: gravelly vacant ground plot
- **Type status:** Other material. **Occurrence:** recordedBy: Zoltán Barina; individualCount: 3; reproductiveCondition: in bloom; occurrenceID: 9E55C8AA-CA66-5BC9-9909-1D04821AFD32; **Taxon:** scientificName: Achilleafilipendulina; family: Asteraceae; taxonRank: species; **Location:** continent: Europe; country: Hungary; county: Budapest; municipality: Budapest VIII.; locality: Rákóczi street; decimalLatitude: 47.499339; decimalLongitude: 19.079543; **Identification:** identifiedBy: Zoltán Barina; **Event:** eventDate: 15/09/2022; habitat: crevices of road pavement
- **Type status:** Other material. **Occurrence:** recordedBy: Zoltán Barina; individualCount: 3; reproductiveCondition: in bloom; occurrenceID: 57833445-6E5C-5BBF-9E75-BB155B13C95A; **Taxon:** scientificName: Achilleafilipendulina; family: Asteraceae; taxonRank: species; **Location:** continent: Europe; country: Hungary; county: Budapest; municipality: Budapest I.; locality: Friedrich Born quay; decimalLatitude: 47.495136; decimalLongitude: 19.043271; **Identification:** identifiedBy: Zoltán Barina; **Event:** eventDate: 30/05/2023; habitat: crevices of road pavement

#### Notes

A plant of south-western and central Asian origin (Liu et al. 2020). The species occurs in several Europaean countries and the USA as an alien escaping from cultivation (Soriano 2014, Wirth et al. 2020a and Eliáš Jr. et al. 2023). These are the first records of the species from Budapest.

### Allium ramosum

L. 1753

3E20632C-3884-5132-9519-154817A8CD05

#### Materials

- **Type status:** Other material. **Occurrence:** recordedBy: Attila Rigó; individualCount: 12; reproductiveCondition: in bloom; occurrenceID: 7D863E20-5DC6-5BF1-BB1E-6BF347D7D451; **Taxon:** scientificName: Alliumramosum s.l.; family: Amaryllidaceae; taxonRank: species; **Location:** continent: Europe; country: Hungary; county: Budapest; municipality: Budapest XXI.; locality: Szent Imre square; decimalLatitude: 47.322186; decimalLongitude: 19.069610; **Identification:** identifiedBy: Attila Rigó; **Event:** eventDate: 04/09/2022; habitat: amenity grassland
- **Type status:** Other material. **Occurrence:** recordedBy: Attila Rigó; individualCount: 10; reproductiveCondition: in bloom; occurrenceID: 7087E948-FA32-55CA-8FA2-B56144712F83; **Taxon:** scientificName: Alliumramosum s.l.; family: Amaryllidaceae; taxonRank: species; **Location:** continent: Europe; country: Hungary; county: Budapest; municipality: Budapest XI.; locality: Csíkihegyek street; decimalLatitude: 47.469866; decimalLongitude: 18.995671; **Identification:** identifiedBy: Attila Rigó; **Event:** eventDate: 18/09/2022; habitat: crevices of pavement
- **Type status:** Other material. **Occurrence:** recordedBy: Attila Rigó; individualCount: 5; reproductiveCondition: in bloom; occurrenceID: 84290FF1-105D-5606-A99F-800D55A2AB0D; **Taxon:** scientificName: Alliumramosum s.l.; family: Amaryllidaceae; taxonRank: species; **Location:** continent: Europe; country: Hungary; county: Budapest; municipality: Budapest IX.; locality: Haller street; decimalLatitude: 47.478302; decimalLongitude: 19.086394; **Identification:** identifiedBy: Attila Rigó; **Event:** eventDate: 25/09/2022; habitat: crevices of pavement

#### Notes

Asian species sometimes cultivated as a vegetable, European introductions are somewhat neglected (Seregin and Korniak 2013). Recently, some subspontaneous occurrences were reported from Pécs (southern Hungary) (Wirth et al. 2020a). These are the first published records of the species from Budapest.

### Artemisia scoparia

Waldst. & Kit. 1801

5EDF8BB9-061F-5BC7-A695-8CFE7A759D08

#### Materials

- **Type status:** Other material. **Occurrence:** recordedBy: Zoltán Barina; individualCount: 15; reproductiveCondition: fruit-bearing; occurrenceID: CF1B3970-08BA-5CEA-8E96-7BE4292B5062; **Taxon:** scientificName: Artemisiascoparia; family: Asteraceae; taxonRank: species; **Location:** continent: Europe; country: Hungary; county: Budapest; municipality: Budapest IX.; locality: Kvassay Jenő road; decimalLatitude: 47.465895; decimalLongitude: 19.075075; **Identification:** identifiedBy: Zoltán Barina; **Event:** eventDate: 26/09/2018; habitat: amenity grassland and urban hedges

#### Notes

This pioneer is native to Hungary and can be found in sandy areas, but had few confirmed records from the country (Király and Király 2018). It had not been found in urban environments before.

### Begonia cucullata

Willd. 1805

2C83972C-E328-56D5-A02C-7239EB0340C5

#### Materials

- **Type status:** Other material. **Occurrence:** recordedBy: Attila Rigó; individualCount: 5; reproductiveCondition: in bloom; occurrenceID: EFA2F61A-06CD-5BD1-B5A3-A19A17DE8FBD; **Taxon:** scientificName: Begoniacucullata; family: Begoniaceae; taxonRank: species; **Location:** continent: Europe; country: Hungary; county: Budapest; municipality: Budapest XI.; locality: Regős street; decimalLatitude: 47.468795; decimalLongitude: 19.002185; **Identification:** identifiedBy: Attila Rigó; **Event:** eventDate: 18/09/2022; habitat: crevices in pavement

#### Notes

A widely cultivated ornamental of South American origin that became naturalised and even invasive in tropical and subtropical regions (Lim 2014). The species only appears as a casual in Europe (e.g. Verloove 2006). There were some recent records of the species from Hungary (Rigó 2019, Rigó and Barina 2020 and Wirth et al. 2020a).

### Borago officinalis

L. 1753

0128233D-6369-5B9F-8734-EEE30490BD68

#### Materials

- **Type status:** Other material. **Occurrence:** recordedBy: Attila Rigó; individualCount: 1; reproductiveCondition: in bloom; occurrenceID: 564ABCC7-0422-5485-BFA2-FA6914507104; **Taxon:** scientificName: Boragoofficinalis; family: Boraginaceae; taxonRank: species; **Location:** continent: Europe; country: Hungary; county: Budapest; municipality: Budapest III.; locality: Hímző street; decimalLatitude: 47.595968; decimalLongitude: 19.059515; **Identification:** identifiedBy: Attila Rigó; **Event:** eventDate: 23/10/2022; habitat: crevice of pavement

#### Notes

An important medicinal plant widely cultivated in the Mediterranean that originates from Syria (Gupta and Singh 2010). The plant has very few records from Hungary (e.g. Haszonits et al. 2021). A rare escape from cultivation.

### Bromus catharticus

Vahl 1791

AB37B794-25FB-5A87-BE37-4ECE16394450

#### Materials

- **Type status:** Other material. **Occurrence:** recordedBy: Attila Rigó; individualCount: 1; reproductiveCondition: in bloom; occurrenceID: 5D6425E3-3BC6-5E8E-823C-5D368BC5233A; **Taxon:** scientificName: Bromuscatharticus; family: Poaceae; taxonRank: species; **Location:** continent: Europe; country: Hungary; county: Budapest; municipality: Budapest III.; locality: Holdudvar park; decimalLatitude: 47.545226; decimalLongitude: 19.031976; **Identification:** identifiedBy: Attila Rigó; **Event:** eventDate: 23/09/2022; habitat: amenity grassland
- **Type status:** Other material. **Occurrence:** recordedBy: Attila Rigó; individualCount: 30; reproductiveCondition: in bloom; occurrenceID: 0A3D6474-F82C-53E2-A6E6-09ACE0F2B56C; **Taxon:** scientificName: Bromuscatharticus; family: Poaceae; taxonRank: species; **Location:** continent: Europe; country: Hungary; county: Budapest; municipality: Budapest XV.; locality: Gábor Áron street; decimalLatitude: 47.548715; decimalLongitude: 19.121553; **Identification:** identifiedBy: Attila Rigó; **Event:** eventDate: 05/11/2022; habitat: road verge
- **Type status:** Other material. **Occurrence:** recordedBy: Attila Rigó; individualCount: 1; reproductiveCondition: in bloom; occurrenceID: BB29B5F1-7805-5C7F-A6DB-D04A84ACBA3E; **Taxon:** scientificName: Bromuscatharticus; family: Poaceae; taxonRank: species; **Location:** continent: Europe; country: Hungary; county: Budapest; municipality: Budapest III.; locality: Gyűrű street; decimalLatitude: 47.598651; decimalLongitude: 19.059620; **Identification:** identifiedBy: Attila Rigó; **Event:** eventDate: 31/05/2023; habitat: crevice of pavement

#### Notes

Native to South America and introduced as a winter forage worldwide (Hamzeh'ee et al. 2007). In Hungary, this species has very few records (Barina 2006, Kovács and Mesterházy 2015, Király and Király 2018 and Molnár et al. 2019). It had not been found in Budapest before.

### Campanula portenschlagiana

Roem. & Schult. 1819

EB0295A0-A26C-5DF1-AEB2-77D31B154A6B

#### Materials

- **Type status:** Other material. **Occurrence:** recordedBy: Attila Rigó; individualCount: 3; reproductiveCondition: in bloom; occurrenceID: 6CEDDF4D-1D47-5012-8DCB-57122450D2D2; **Taxon:** scientificName: Campanulaportenschlagiana; family: Campanulaceae; taxonRank: species; **Location:** continent: Europe; country: Hungary; county: Budapest; municipality: Budapest XII.; locality: Avar street; decimalLatitude: 47.492147; decimalLongitude: 19.027990; **Identification:** identifiedBy: Attila Rigó; **Event:** eventDate: 13/05/2022; habitat: crack at the base of a building

#### Notes

A lesser-known rock garden plant in Hungary, which superficially resembles *Campanulaposcharskyana* Degen (Dunkel 2006). Naturalised in Belgium (Verloove 2006). This is the first record of the species from Hungary.

### Catapodium rigidum

(L.) C.E.Hubb. 1953

82ED1A53-BB7E-538B-90E2-989B380DA543

#### Materials

- **Type status:** Other material. **Occurrence:** recordedBy: Attila Rigó; individualCount: 400; reproductiveCondition: in bloom; occurrenceID: E462FC53-17F9-5261-91C6-A50EAF89A1F7; **Taxon:** scientificName: Catapodiumrigidum; family: Poaceae; taxonRank: species; **Location:** continent: Europe; country: Hungary; county: Budapest; municipality: Budapest III.; locality: Gyűrű street; decimalLatitude: 47.598549; decimalLongitude: 19.060334; **Identification:** identifiedBy: Attila Rigó; **Event:** eventDate: 31/05/2023; habitat: small trampled lawn patch and crevices of pavement

#### Notes

Native to Europe and the Mediterranean, while it appears as an alien in Australia, Africa, Asia, South and North America (Bhat et al. 2021). It is not native to Hungary, but there had been two previous records from western Hungary (Solymosi 2008 and Schmidt 2019). The population found in Budapest is its north-easternmost recorded occurrence in Hungary.

### Cenchrus alopecuroides

(L.) Thunb. 1794

18ABC722-BE6C-5646-A0F0-1AB662C0DADA

#### Materials

- **Type status:** Other material. **Occurrence:** recordedBy: Attila Rigó; individualCount: 8; reproductiveCondition: in bloom; occurrenceID: B6FDE991-C377-5705-A62B-9625811D23AC; **Taxon:** scientificName: Cenchrusalopecuroides; family: Poaceae; taxonRank: species; **Location:** continent: Europe; country: Hungary; county: Budapest; municipality: Budapest XI.; locality: ELTE campus; decimalLatitude: 47.473569; decimalLongitude: 19.060760; **Identification:** identifiedBy: Attila Rigó; **Event:** eventDate: 13/08/2022; habitat: lawn
- **Type status:** Other material. **Occurrence:** recordedBy: Attila Rigó; individualCount: 1; reproductiveCondition: freuit-bearing; occurrenceID: 7B54E486-AF2A-5454-8DCC-C6A31E3F0A51; **Taxon:** scientificName: Cenchrusalopecuroides; family: Poaceae; taxonRank: species; **Location:** continent: Europe; country: Hungary; county: Budapest; municipality: Budapest XV.; locality: Kinizsi street; decimalLatitude: 47.546916; decimalLongitude: 19.112470; **Identification:** identifiedBy: Attila Rigó; **Event:** eventDate: 05/11/2022; habitat: crevices of pavement

#### Notes

A perennial ornamental grass that originates from Asia and Australia which was introduced worldwide and became naturalised in the USA (Deme et al. 2019). In Hungary, the species was first recorded in 2019 and its records are increasing (Deme et al. 2019 and Schmidt and Haszonits 2021).

### Chasmanthium latifolium

(Michx.) H.O.Yates 1966

F4CBB729-6580-545C-8A14-E3A50E5F64F4

#### Materials

- **Type status:** Other material. **Occurrence:** recordedBy: Attila Rigó; individualCount: 6; reproductiveCondition: in bloom; occurrenceID: 0A07816F-598B-5200-95CF-E94B6AAC039E; **Taxon:** scientificName: Chasmanthiumlatifolium; family: Poaceae; taxonRank: species; **Location:** continent: Europe; country: Hungary; county: Budapest; municipality: Budapest VIII.; locality: Mindszenthy József square; decimalLatitude: 47.491792; decimalLongitude: 19.075540; **Identification:** identifiedBy: Attila Rigó; **Event:** eventDate: 10/10/2021; habitat: urban hedge

#### Notes

A riparian plant endemic to the south-eastern part of the USA (Sanchez-Ken and Clark 2023) and used as an ornamental plant in other parts of the world (Chelariu 2017). This is its first documented escape from cultivation in Hungary.

### Clinopodium nepeta

(L.) Kuntze 1891

275FF48B-7B1A-5B7A-9A31-9039E5C227ED

#### Materials

- **Type status:** Other material. **Occurrence:** recordedBy: Attila Rigó; individualCount: 6; reproductiveCondition: in bloom; occurrenceID: 2B8DCA19-04BA-5987-8191-06A0230BB283; **Taxon:** scientificName: Clinopdiumnepeta; family: Lamiaceae; taxonRank: species; **Location:** continent: Europe; country: Hungary; county: Budapest; municipality: Margaret Island; locality: ruins of the Dominican nunnery; decimalLatitude: 47.529275; decimalLongitude: 19.051076; **Identification:** identifiedBy: Attila Rigó; **Event:** eventDate: 25/06/2019; habitat: on disturbed ground
- **Type status:** Other material. **Occurrence:** recordedBy: Attila Rigó; individualCount: 2; reproductiveCondition: in bloom; occurrenceID: 11BD2AA6-804F-5F62-B2EC-1E735DA79908; **Taxon:** scientificName: Clinopdiumnepeta; family: Lamiaceae; taxonRank: species; **Location:** continent: Europe; country: Hungary; county: Budapest; municipality: Budapest IX.; locality: Likőr street; decimalLatitude: 47.477753; decimalLongitude: 19.072751; **Identification:** identifiedBy: Attila Rigó; **Event:** eventDate: 31/07/2022; habitat: crevices of pavement
- **Type status:** Other material. **Occurrence:** recordedBy: Attila Rigó; individualCount: 3; reproductiveCondition: fruit-bearing; occurrenceID: C93BD580-B690-5877-9D7D-FC6A5C1ACD36; **Taxon:** scientificName: Clinopdiumnepeta; family: Lamiaceae; taxonRank: species; **Location:** continent: Europe; country: Hungary; county: Budapest; municipality: Budapest III.; locality: Bogdáni road; decimalLatitude: 47.549862; decimalLongitude: 19.046616; **Identification:** identifiedBy: Attila Rigó; **Event:** eventDate: 29/09/2022; habitat: crack at the base of an industrial building

#### Notes

Ornamental and medicinal plant that originates from the Mediterranean and is rarely cultivated worldwide; sometimes it escapes from cultivation (González-Gallegos et al. 2017). These are its first records from Hungary.

### Cyperus eragrostis

Lam. 1791

F21BB0BA-B068-5236-A9E4-57137EAD3249

#### Materials

- **Type status:** Other material. **Occurrence:** recordedBy: Attila Rigó, Ákos Malatinszky; individualCount: 4; reproductiveCondition: fruit-bearing; occurrenceID: 86215EC2-B0FC-5239-BCB2-94576C287559; **Taxon:** scientificName: Cyperuseragrostis; family: Cyperaceae; taxonRank: species; **Location:** continent: Europe; country: Hungary; county: Budapest; municipality: Budapest III.; locality: Ágoston street; decimalLatitude: 47.545764; decimalLongitude: 19.031707; **Identification:** identifiedBy: Attila Rigó; **Event:** eventDate: 23/09/2022; habitat: irrigated lawn in residential area
- **Type status:** Other material. **Occurrence:** recordedBy: Attila Rigó; individualCount: 3; reproductiveCondition: fruit-bearing; occurrenceID: 886EA755-6A31-53BA-B1FF-ACBF05618556; **Taxon:** scientificName: Cyperuseragrostis; family: Cyperaceae; taxonRank: species; **Location:** continent: Europe; country: Hungary; county: Budapest; municipality: Budapest VIII.; locality: ELTE Botanical Garden; decimalLatitude: 47.485348; decimalLongitude: 19.084849; **Identification:** identifiedBy: Attila Rigó; **Event:** eventDate: 26/10/2022; habitat: flower pot in botanical garden
- **Type status:** Other material. **Occurrence:** recordedBy: Attila Rigó; individualCount: 2; reproductiveCondition: fruit-bearing; occurrenceID: 561D2ED8-0B11-5534-9204-79FC7897BC51; **Taxon:** scientificName: Cyperuseragrostis; family: Cyperaceae; taxonRank: species; **Location:** continent: Europe; country: Hungary; county: Budapest; municipality: Budapest X.; locality: Kőbányai road; decimalLatitude: 47.483073; decimalLongitude: 19.117633; **Identification:** identifiedBy: Attila Rigó; **Event:** eventDate: 28/10/2022; habitat: flower pot in garden store

#### Notes

Native to South America, but occurs in southern, western and central Europe, North Africa and North America as an alien introduced as an ornamental (Mokni and Verloove 2021), with some recent Hungarian records (Mesterházy 2021). These are the first records of the plant from Budapest. Aside from these records, the authors found a small population at the bank of the Danube, north of Budapest. The plant might become invasive.

### Cyrtomium falcatum

(L.f.) C.Presl 1836

550680E5-13C7-5E3E-9BD0-1986601369E3

#### Materials

- **Type status:** Other material. **Occurrence:** recordedBy: Attila Rigó; individualCount: 1; reproductiveCondition: non-reproductive; occurrenceID: A8E1477E-E92A-5D34-8FDB-0C7A79FB8B57; **Taxon:** scientificName: Cyrtomiumfalcatum; family: Dryopteridaceae; taxonRank: species; **Location:** continent: Europe; country: Hungary; county: Budapest; municipality: Budapest XXI.; locality: Vegyigépgyár street; decimalLatitude: 47.426498; decimalLongitude: 19.054428; **Identification:** identifiedBy: Attila Rigó; **Event:** eventDate: 21/10/2022; habitat: mortar of old brick wall of an industrial building

#### Notes

This species is an evergreen ornamental fern native to East Asia and introduced to North America and Europe (Maslo 2022). It is considered potentially invasive in South Africa (McCulloch-Jones et al. 2021). The first report from Hungary was by Tamás et al. (2017). This is its second record from Hungary.

### Cyrtomium fortunei

J.Sm. 1866

56F4C48F-1A21-5AD1-9B03-ECDEEE251E21

#### Materials

- **Type status:** Other material. **Occurrence:** recordedBy: Attila Rigó; individualCount: 1; reproductiveCondition: non-reproductive; occurrenceID: A5E062AF-E26B-5236-BB1E-732C75989D46; **Taxon:** scientificName: Cyrtomiumfortunei; family: Dryopteridaceae; taxonRank: species; **Location:** continent: Europe; country: Hungary; county: Budapest; municipality: Budapest VII.; locality: Pétery Sándor Hospital; decimalLatitude: 47.502904; decimalLongitude: 19.079430; **Identification:** identifiedBy: Attila Rigó; **Event:** eventDate: 04/11/2022; habitat: mortar of old brick wall of a hospital building

#### Notes

An East Asian fern that is cultivated as an ornamental and occurs as a garden escapee in European countries, recently reported from Slovenia (Jogan et al. 2022). This is the first record of the plant from Hungary.

### Datura innoxia

Mill. 1768

BBDCE8F1-5104-512C-82A0-4AEE257EBE9E

#### Materials

- **Type status:** Other material. **Occurrence:** recordedBy: Attila Rigó; individualCount: 4; reproductiveCondition: in bloom; occurrenceID: 74E672F1-4917-58AA-8C6F-FD88D0D8C9CE; **Taxon:** scientificName: Daturainnoxia; family: Solanaceae; taxonRank: species; **Location:** continent: Europe; country: Hungary; county: Budapest; municipality: Budapest IV.; locality: Rózsa street; decimalLatitude: 47.564219; decimalLongitude: 19.099698; **Identification:** identifiedBy: Attila Rigó; **Event:** eventDate: 12/10/2022; habitat: cracks at the base of a wall

#### Notes

Native to Central and South America and planted as a rare ornamental and medicinal plant in Europe, which occasionally escapes from cultivation (Maslo and Šarić 2019). In Hungary, it has few confirmed records and is usually intermixed with *Daturawrightii* Regel (Király et al. 2009), which is more abundant in Budapest in our opinion.

### Datura wrightii

Regel 1859

CF6ADCDA-41DD-520A-9691-9332AF3E99B7

#### Materials

- **Type status:** Other material. **Occurrence:** recordedBy: Zoltán Barina; individualCount: 1; reproductiveCondition: in bloom; occurrenceID: FC97A977-D1C4-5D4B-AD3F-5620347D2B3E; **Taxon:** scientificName: Daturawrightii; family: Solanaceae; taxonRank: species; **Location:** continent: Europe; country: Hungary; county: Budapest; municipality: Budapest VIII.; locality: Orczy square; decimalLatitude: 47.489043; decimalLongitude: 19.091997; **Identification:** identifiedBy: Attila Rigó; **Event:** eventDate: 01/10/2018; habitat: amenity grassland
- **Type status:** Other material. **Occurrence:** recordedBy: Zoltán Barina; individualCount: 3; reproductiveCondition: in bloom; occurrenceID: 13AEC779-6638-5880-8263-69178E4EEF79; **Taxon:** scientificName: Daturawrightii; family: Solanaceae; taxonRank: species; **Location:** continent: Europe; country: Hungary; county: Budapest; municipality: Budapest XIII.; locality: Vág street; decimalLatitude: 47.524098; decimalLongitude: 19.057650; **Identification:** identifiedBy: Attila Rigó; **Event:** eventDate: 03/11/2018; habitat: front garden
- **Type status:** Other material. **Occurrence:** recordedBy: Attila Rigó, Ákos Malatinszky; individualCount: 3; reproductiveCondition: in bloom; occurrenceID: 60A340CC-55C1-5FDE-85FE-7F019CD77111; **Taxon:** scientificName: Daturawrightii; family: Solanaceae; taxonRank: species; **Location:** continent: Europe; country: Hungary; county: Budapest; municipality: Budapest XXI.; locality: Szent Imre square; decimalLatitude: 47.432132; decimalLongitude: 19.068959; **Identification:** identifiedBy: Attila Rigó; **Event:** eventDate: 04/09/2022; habitat: road verge and crevices of pavement
- **Type status:** Other material. **Occurrence:** recordedBy: Attila Rigó; individualCount: 8; reproductiveCondition: in bloom; occurrenceID: 7695FDE6-7CBC-5915-8076-94781D598A2B; **Taxon:** scientificName: Daturawrightii; family: Solanaceae; taxonRank: species; **Location:** continent: Europe; country: Hungary; county: Budapest; municipality: Budapest XXI.; locality: Templom street; decimalLatitude: 47.432934; decimalLongitude: 19.062990; **Identification:** identifiedBy: Attila Rigó; **Event:** eventDate: 04/09/2022; habitat: road verge and crevices of pavement
- **Type status:** Other material. **Occurrence:** recordedBy: Attila Rigó; individualCount: 2; reproductiveCondition: in bloom; occurrenceID: 8D71076D-0918-5A43-B445-895677F6FEF7; **Taxon:** scientificName: Daturawrightii; family: Solanaceae; taxonRank: species; **Location:** continent: Europe; country: Hungary; county: Budapest; municipality: Budapest XXII.; locality: Vár street; decimalLatitude: 47.424662; decimalLongitude: 19.037695; **Identification:** identifiedBy: Attila Rigó; **Event:** eventDate: 11/09/2022; habitat: road verge and crevices of pavement
- **Type status:** Other material. **Occurrence:** recordedBy: Attila Rigó; individualCount: 10; reproductiveCondition: in bloom; occurrenceID: E7082AD4-4BCA-542A-8B33-DF259A6D5BFC; **Taxon:** scientificName: Daturawrightii; family: Solanaceae; taxonRank: species; **Location:** continent: Europe; country: Hungary; county: Budapest; municipality: Budapest XI.; locality: Csíkihegyek street; decimalLatitude: 47.470830; decimalLongitude: 18.994885; **Identification:** identifiedBy: Attila Rigó; **Event:** eventDate: 18/09/2022; habitat: crevices of pavement
- **Type status:** Other material. **Occurrence:** recordedBy: Attila Rigó; individualCount: 1; reproductiveCondition: non-reproductive; occurrenceID: E7206307-0246-5FE9-965A-818908B7803C; **Taxon:** scientificName: Daturawrightii; family: Solanaceae; taxonRank: species; **Location:** continent: Europe; country: Hungary; county: Budapest; municipality: Budapest IX.; locality: Haller street; decimalLatitude: 47.477706; decimalLongitude: 19.084978; **Identification:** identifiedBy: Attila Rigó; **Event:** eventDate: 25/09/2022; habitat: crack at the base of a building
- **Type status:** Other material. **Occurrence:** recordedBy: Attila Rigó; individualCount: 5; reproductiveCondition: fruit-bearing; occurrenceID: 3CBA7808-619E-5AF6-8C6E-2F70B4DCC77C; **Taxon:** scientificName: Daturawrightii; family: Solanaceae; taxonRank: species; **Location:** continent: Europe; country: Hungary; county: Budapest; municipality: Budapest XV.; locality: M3 motorway; decimalLatitude: 47.549794; decimalLongitude: 19.122221; **Identification:** identifiedBy: Attila Rigó; **Event:** eventDate: 07/11/2022; habitat: disturbed ground

#### Description

A somewhat neglected ornamental and medicinal plant of Central American origin, which is planted occasionally in Europe (Verloove 2008). Invasive in some parts of southern Europe (Verloove 2008) and has recently become invasive in Romania (Niculescu 2022). In Hungary, it has very few records and appears as a locally naturalised alien (Király et al. 2009). Very much resembles *Daturainnoxia* Mill. and is distinguished, based on stem indumentum (Király et al. 2009).

### Digitaria ciliaris

(Retz.) Koeler 1802

1879AE53-9400-5985-BCD6-E9DF0FE8ECF7

#### Materials

- **Type status:** Other material. **Occurrence:** recordedBy: Attila Rigó; individualCount: 30; reproductiveCondition: fruit-bearing; occurrenceID: 8AA8BBF4-C9CB-5147-B812-CF7D31A44550; **Taxon:** scientificName: Digitariaciliaris; family: Poaceae; taxonRank: species; **Location:** continent: Europe; country: Hungary; county: Budapest; municipality: Budapest VIII.; locality: Corvin alley; decimalLatitude: 47.486308; decimalLongitude: 19.071415; **Identification:** identifiedBy: Attila Rigó; **Event:** eventDate: 19/06/2022; habitat: crack at the base of a wall
- **Type status:** Other material. **Occurrence:** recordedBy: Attila Rigó; individualCount: 20; reproductiveCondition: fruit-bearing; occurrenceID: F64B63C4-269D-5A12-BD0A-5EA5FF9DD99B; **Taxon:** scientificName: Digitariaciliaris; family: Poaceae; taxonRank: species; **Location:** continent: Europe; country: Hungary; county: Budapest; municipality: Budapest II.; locality: Rét street; decimalLatitude: 47.513422; decimalLongitude: 19.024173; **Identification:** identifiedBy: Attila Rigó; **Event:** eventDate: 06/07/2022; habitat: crevices of pavement

#### Notes

A rather rare and neglected weed of the Hungarian flora (Király 2009). New to Budapest.

### Eclipta prostrata

(L.) L. 1771

9DB58D46-82AA-5AB7-B210-6E088D1F24C9

#### Materials

- **Type status:** Other material. **Occurrence:** recordedBy: Attila Rigó; individualCount: 5; reproductiveCondition: fruit-bearing; occurrenceID: C0C595D9-D32F-5624-AAC5-DF44DD4B26DF; **Taxon:** scientificName: Ecliptaprostrata; family: Asteraceae; taxonRank: species; **Location:** continent: Europe; country: Hungary; county: Budapest; municipality: Budapest III.; locality: Árpád street; decimalLatitude: 47.590467; decimalLongitude: 19.051000; **Identification:** identifiedBy: Attila Rigó; **Event:** eventDate: 28/09/2022; habitat: flower boxes in a garden store
- **Type status:** Other material. **Occurrence:** recordedBy: Attila Rigó; individualCount: 8; reproductiveCondition: fruit-bearing; occurrenceID: 6DEDE7AC-AB34-50C3-9A9B-DAEF209A7A18; **Taxon:** scientificName: Ecliptaprostrata; family: Asteraceae; taxonRank: species; **Location:** continent: Europe; country: Hungary; county: Budapest; municipality: Budapest II.; locality: Zilah street; decimalLatitude: 47.515390; decimalLongitude: 19.010740; **Identification:** identifiedBy: Attila Rigó; **Event:** eventDate: 22/10/2022; habitat: crevices of pavement in a garden store

#### Notes

Native to Asia, but introduced as a medicinal plant throughout subtropical and tropical regions (Duc et al. 2021) and spreading as an alien weed in Europe as well (Jeričević and Jeričević 2017). In Hungary, many of its records are from garden stores (Takács et al. 2020), but a population was discovered on the shore of the Danube (Mesterházy 2021). New to Budapest.

### Eragrostis spectabilis

(Pursh) Steud. 1840

B9C466EF-9CE7-56B5-9440-4AE26A6AF04A

#### Materials

- **Type status:** Other material. **Occurrence:** recordedBy: Attila Rigó; individualCount: 10; reproductiveCondition: fruit-bearing; occurrenceID: 6FF399E9-40A9-59AE-9546-B32AA41C7696; **Taxon:** scientificName: Eragrostisspectabilis; family: Poaceae; taxonRank: species; **Location:** continent: Europe; country: Hungary; county: Budapest; municipality: Budapest II.; locality: Margit boulevard; decimalLatitude: 47.509142; decimalLongitude: 19.027732; **Identification:** identifiedBy: Attila Rigó; **Event:** eventDate: 01/09/2022; habitat: crevices of pavement

#### Notes

A perennial grass native to North America which is used as an ornamental in Europe and Asia. Some studies suggest that it could potentially become invasive (Qin and Wu 2013 and Wirth et al. 2020a). This species had only been recorded once in Hungary, with one specimen found in a flower bed near the parent plants at Pécs (S Hungary) (Wirth et al. 2020a). In the new locality in Budapest, we could not find the propagule source nearby.

### Eragrostis virescens

J.Presl 1830

63A7C5BA-23EB-5B08-AAEF-E7D851361F73

#### Materials

- **Type status:** Other material. **Occurrence:** recordedBy: Attila Rigó, Ákos Malatinszky; individualCount: 2; reproductiveCondition: fruit-bearing; occurrenceID: 274CA359-66DF-58EF-88E4-B0B17BAC5A66; **Taxon:** scientificName: Eragrostisvirescens; family: Poaceae; taxonRank: species; **Location:** continent: Europe; country: Hungary; county: Budapest; municipality: Budapest I.; locality: Döbrentei square; decimalLatitude: 47.490624; decimalLongitude: 19.045969; **Identification:** identifiedBy: Attila Rigó; **Event:** eventDate: 05/09/2022; habitat: flower bed
- **Type status:** Other material. **Occurrence:** recordedBy: Attila Rigó; individualCount: 8; reproductiveCondition: fruit-bearing; occurrenceID: F3B40299-BBDD-5B21-B46A-4C09406E4F07; **Taxon:** scientificName: Eragrostisvirescens; family: Poaceae; taxonRank: species; **Location:** continent: Europe; country: Hungary; county: Budapest; municipality: Budapest XIV.; locality: Városliget; decimalLatitude: 47.512438; decimalLongitude: 19.082074; **Identification:** identifiedBy: Attila Rigó; **Event:** eventDate: 14/09/2022; habitat: flower bed

#### Notes

Native to North, Central and South America and introduced to Europe, South Africa and Australia. The first record of this species in Hungary was published in 1929 by Antal Pénzes (as *E.neo-mexicana* Vasey) and, recently, some small populations were found in the country (Király et al. 2019 and Rigó and Barina 2020).

### Erigeron sumatrensis

Retz. 1788

22131192-3382-5926-A4E1-837BA5DBF562

#### Materials

- **Type status:** Other material. **Occurrence:** recordedBy: Attila Rigó; individualCount: 2; reproductiveCondition: non-reproductive; occurrenceID: 6C528509-4B29-51A1-A7B6-21EEB95D87EE; **Taxon:** scientificName: Erigeronsumatrensis; family: Asteraceae; taxonRank: species; **Location:** continent: Europe; country: Hungary; county: Budapest; municipality: Budapest III.; locality: Vízimolnár street; decimalLatitude: 47.568365; decimalLongitude: 19.053618; **Identification:** identifiedBy: Attila Rigó; **Event:** eventDate: 15/07/2022; habitat: lawn in residential area
- **Type status:** Other material. **Occurrence:** recordedBy: Attila Rigó; individualCount: 2; reproductiveCondition: in bloom; occurrenceID: 77E0A6C6-B508-5A9C-9BA6-5880AF0501F0; **Taxon:** scientificName: Erigeronsumatrensis; family: Asteraceae; taxonRank: species; **Location:** continent: Europe; country: Hungary; county: Budapest; municipality: Budapest XIV.; locality: Zalán street; decimalLatitude: 47.504394; decimalLongitude: 19.142996; **Identification:** identifiedBy: Attila Rigó; **Event:** eventDate: 30/07/2022; habitat: crevices of pavement in a garden store
- **Type status:** Other material. **Occurrence:** recordedBy: Attila Rigó; individualCount: 8; reproductiveCondition: fruit bearing; occurrenceID: 92E274D0-CE98-50CC-AC50-86F76675C797; **Taxon:** scientificName: Erigeronsumatrensis; family: Asteraceae; taxonRank: species; **Location:** continent: Europe; country: Hungary; county: Budapest; municipality: Budapest XXI.; locality: Célgép street; decimalLatitude: 47.420865; decimalLongitude: 19.054745; **Identification:** identifiedBy: Attila Rigó; **Event:** eventDate: 04/09/2022; habitat: road verge
- **Type status:** Other material. **Occurrence:** recordedBy: Attila Rigó; individualCount: 2; reproductiveCondition: fruit bearing; occurrenceID: 9F990E79-2524-5C22-9F17-BB33FCA13889; **Taxon:** scientificName: Erigeronsumatrensis; family: Asteraceae; taxonRank: species; **Location:** continent: Europe; country: Hungary; county: Budapest; municipality: Budapest XI.; locality: Törökugrató street; decimalLatitude: 47.469426; decimalLongitude: 18.999988; **Identification:** identifiedBy: Attila Rigó; **Event:** eventDate: 18/09/2022; habitat: crevices of pavement
- **Type status:** Other material. **Occurrence:** recordedBy: Attila Rigó; individualCount: 3; reproductiveCondition: in bloom; occurrenceID: FD5449EF-F986-5C0B-B702-15E45335DE48; **Taxon:** scientificName: Erigeronsumatrensis; family: Asteraceae; taxonRank: species; **Location:** continent: Europe; country: Hungary; county: Budapest; municipality: Budapest III.; locality: Holdudvar park; decimalLatitude: 47.545814; decimalLongitude: 19.031568; **Identification:** identifiedBy: Attila Rigó; **Event:** eventDate: 23/09/2022; habitat: lawn in residential area
- **Type status:** Other material. **Occurrence:** recordedBy: Attila Rigó; individualCount: 4; reproductiveCondition: fruit bearing; occurrenceID: AB6003CB-E406-5754-8CF8-CC0C7F17C658; **Taxon:** scientificName: Erigeronsumatrensis; family: Asteraceae; taxonRank: species; **Location:** continent: Europe; country: Hungary; county: Budapest; municipality: Budapest IX.; locality: Szent István Hospital; decimalLatitude: 47.477145; decimalLongitude: 19.088545; **Identification:** identifiedBy: Attila Rigó; **Event:** eventDate: 25/09/2022; habitat: crack at the base of a hospital building
- **Type status:** Other material. **Occurrence:** recordedBy: Attila Rigó; individualCount: 120; reproductiveCondition: fruit bearing; occurrenceID: 2D09D538-ABF9-5BEE-B899-DB0D4189FA68; **Taxon:** scientificName: Erigeronsumatrensis; family: Asteraceae; taxonRank: species; **Location:** continent: Europe; country: Hungary; county: Budapest; municipality: Budapest III.; locality: Árpád street; decimalLatitude: 47.590993; decimalLongitude: 19.052483; **Identification:** identifiedBy: Attila Rigó; **Event:** eventDate: 28/09/2022; habitat: gravelly places, lawns, crevices of road pavement and flower beds in a garden store
- **Type status:** Other material. **Occurrence:** recordedBy: Attila Rigó; individualCount: 70; reproductiveCondition: fruit bearing; occurrenceID: 94E1C16B-7351-5750-AB5C-8426EEBD9EAC; **Taxon:** scientificName: Erigeronsumatrensis; family: Asteraceae; taxonRank: species; **Location:** continent: Europe; country: Hungary; county: Budapest; municipality: Budapest IX.; locality: Illatos road; decimalLatitude: 47.454848; decimalLongitude: 19.098770; **Identification:** identifiedBy: Attila Rigó; **Event:** eventDate: 24/10/2022; habitat: disturbed road slpoe
- **Type status:** Other material. **Occurrence:** recordedBy: Attila Rigó; individualCount: 2; reproductiveCondition: fruit bearing; occurrenceID: BE6F4F51-296A-5772-AB40-E0BFD6E493DF; **Taxon:** scientificName: Erigeronsumatrensis; family: Asteraceae; taxonRank: species; **Location:** continent: Europe; country: Hungary; county: Budapest; municipality: Budapest IX.; locality: Soroksári road; decimalLatitude: 47.468196; decimalLongitude: 19.075402; **Identification:** identifiedBy: Attila Rigó; **Event:** eventDate: 24/10/2022; habitat: crevices of road pavement
- **Type status:** Other material. **Occurrence:** recordedBy: Attila Rigó; individualCount: 3; reproductiveCondition: fruit bearing; occurrenceID: 29BA6892-FF8E-5FD9-9ABD-0E88B24EF9EE; **Taxon:** scientificName: Erigeronsumatrensis; family: Asteraceae; taxonRank: species; **Location:** continent: Europe; country: Hungary; county: Budapest; municipality: Budapest XV.; locality: Alag street; decimalLatitude: 47.580112; decimalLongitude: 19.119699; **Identification:** identifiedBy: Attila Rigó; **Event:** eventDate: 09/11/2022; habitat: road verge
- **Type status:** Other material. **Occurrence:** recordedBy: Attila Rigó; individualCount: 4; reproductiveCondition: fruit bearing; occurrenceID: E886FAA5-FF7A-5CCF-8324-68A2EE6A7B43; **Taxon:** scientificName: Erigeronsumatrensis; family: Asteraceae; taxonRank: species; **Location:** continent: Europe; country: Hungary; county: Budapest; municipality: Budapest XI.; locality: Bartók Béla road; decimalLatitude: 47.471007; decimalLongitude: 19.028059; **Identification:** identifiedBy: Attila Rigó; **Event:** eventDate: 17/11/2022; habitat: crack at the base of a building

#### Notes

A thermophilous weed native to South America spreading in warm regions and introduced in many European countries (Maslo and Šarić 2021). In Hungary, it was first found in Pécs (southern Hungary) (Wirth and Csiky 2020). New to Budapest. The plant has some large populations in Budapest, which suggests that it has become locally naturalised.

### Euphorbia prostrata

Aiton 1789

C8294452-558B-5118-ABF7-8F50A5B29E2C

#### Materials

- **Type status:** Other material. **Occurrence:** recordedBy: Attila Rigó; individualCount: 20; reproductiveCondition: fruit-bearing; occurrenceID: 652EF125-FA6D-541B-A447-E9C64944742F; **Taxon:** scientificName: Euphorbiaprostrata; family: Euphorbiaceae; taxonRank: species; **Location:** continent: Europe; country: Hungary; county: Budapest; municipality: Budapest VIII.; locality: Rákóczi square; decimalLatitude: 47.493036; decimalLongitude: 19.072113; **Identification:** identifiedBy: Attila Rigó; **Event:** eventDate: 17/06/2022; habitat: flower bed
- **Type status:** Other material. **Occurrence:** recordedBy: Attila Rigó; individualCount: 150; reproductiveCondition: fruit-bearing; occurrenceID: 55E03241-25C8-57DA-9BBF-A8F642C8E362; **Taxon:** scientificName: Euphorbiaprostrata; family: Euphorbiaceae; taxonRank: species; **Location:** continent: Europe; country: Hungary; county: Budapest; municipality: Budapest II.; locality: Millenáris park; decimalLatitude: 47.509776; decimalLongitude: 19.027559; **Identification:** identifiedBy: Attila Rigó; **Event:** eventDate: 06/07/2022; habitat: flower bed
- **Type status:** Other material. **Occurrence:** recordedBy: Attila Rigó; individualCount: 150; reproductiveCondition: fruit-bearing; occurrenceID: 60EE153E-4EF9-540A-801A-52D9F1870EF8; **Taxon:** scientificName: Euphorbiaprostrata; family: Euphorbiaceae; taxonRank: species; **Location:** continent: Europe; country: Hungary; county: Budapest; municipality: Budapest XV.; locality: Pólus Center; decimalLatitude: 47.552816; decimalLongitude: 19.141799; **Identification:** identifiedBy: Attila Rigó; **Event:** eventDate: 10/07/2022; habitat: flower beds and flower boxes
- **Type status:** Other material. **Occurrence:** recordedBy: Attila Rigó; individualCount: 100; reproductiveCondition: fruit-bearing; occurrenceID: 55308800-B141-53A3-84D8-BD4D0AE3877F; **Taxon:** scientificName: Euphorbiaprostrata; family: Euphorbiaceae; taxonRank: species; **Location:** continent: Europe; country: Hungary; county: Budapest; municipality: Budapest XIV.; locality: Zalán street; decimalLatitude: 47.504579; decimalLongitude: 19.143785; **Identification:** identifiedBy: Attila Rigó; **Event:** eventDate: 31/07/2022; habitat: crevices in pavement and flower boxes in a garden store
- **Type status:** Other material. **Occurrence:** recordedBy: Attila Rigó, Ákos Malatinszky; individualCount: 30; reproductiveCondition: fruit-bearing; occurrenceID: 5615DFC2-BCAB-5ABB-9E45-82367222AE1F; **Taxon:** scientificName: Euphorbiaprostrata; family: Euphorbiaceae; taxonRank: species; **Location:** continent: Europe; country: Hungary; county: Budapest; municipality: Budapest VIII.; locality: Múzeumkert; decimalLatitude: 47.491698; decimalLongitude: 19.061867; **Identification:** identifiedBy: Attila Rigó; **Event:** eventDate: 10/08/2022; habitat: planting pit for trees and lawn
- **Type status:** Other material. **Occurrence:** recordedBy: Attila Rigó; individualCount: 20; reproductiveCondition: fruit-bearing; occurrenceID: E888BC3E-53DD-513E-8F46-65FE910505A8; **Taxon:** scientificName: Euphorbiaprostrata; family: Euphorbiaceae; taxonRank: species; **Location:** continent: Europe; country: Hungary; county: Budapest; municipality: Budapest XI.; locality: ELTE campus; decimalLatitude: 47.473115; decimalLongitude: 19.060514; **Identification:** identifiedBy: Attila Rigó; **Event:** eventDate: 13/08/2022; habitat: crevices in pavement
- **Type status:** Other material. **Occurrence:** recordedBy: Attila Rigó; individualCount: 20; reproductiveCondition: fruit-bearing; occurrenceID: 1183045E-2D93-5AA7-8B37-220105A870FD; **Taxon:** scientificName: Euphorbiaprostrata; family: Euphorbiaceae; taxonRank: species; **Location:** continent: Europe; country: Hungary; county: Budapest; municipality: Budapest XI.; locality: Infopark promenade; decimalLatitude: 47.470581; decimalLongitude: 19.060040; **Identification:** identifiedBy: Attila Rigó; **Event:** eventDate: 13/08/2022; habitat: lawn and crevices of road pavement
- **Type status:** Other material. **Occurrence:** recordedBy: Attila Rigó; individualCount: 20; reproductiveCondition: fruit-bearing; occurrenceID: 032B26D4-2AD8-53F0-9B9B-9DFE88352F54; **Taxon:** scientificName: Euphorbiaprostrata; family: Euphorbiaceae; taxonRank: species; **Location:** continent: Europe; country: Hungary; county: Budapest; municipality: Budapest IV.; locality: Árpád street; decimalLatitude: 47.560381; decimalLongitude: 19.083281; **Identification:** identifiedBy: Attila Rigó; **Event:** eventDate: 06/09/2022; habitat: flower bed
- **Type status:** Other material. **Occurrence:** recordedBy: Attila Rigó; individualCount: 10; reproductiveCondition: fruit-bearing; occurrenceID: 034B71D9-4402-58C0-A8AE-CA3EDD19378B; **Taxon:** scientificName: Euphorbiaprostrata; family: Euphorbiaceae; taxonRank: species; **Location:** continent: Europe; country: Hungary; county: Budapest; municipality: Budapest XVI.; locality: Lassú street; decimalLatitude: 47.521718; decimalLongitude: 19.222590; **Identification:** identifiedBy: Attila Rigó; **Event:** eventDate: 07/09/2022; habitat: crevices in pavement
- **Type status:** Other material. **Occurrence:** recordedBy: Attila Rigó; individualCount: 20; reproductiveCondition: fruit-bearing; occurrenceID: A0C15287-E067-5BD0-B71A-0AEF5734916A; **Taxon:** scientificName: Euphorbiaprostrata; family: Euphorbiaceae; taxonRank: species; **Location:** continent: Europe; country: Hungary; county: Budapest; municipality: Budapest XVI.; locality: Levedi street; decimalLatitude: 47.529780; decimalLongitude: 19.214648; **Identification:** identifiedBy: Attila Rigó; **Event:** eventDate: 07/09/2022; habitat: front garden
- **Type status:** Other material. **Occurrence:** recordedBy: Attila Rigó; individualCount: 5; reproductiveCondition: fruit-bearing; occurrenceID: F55ED372-E798-584E-B768-BA437A52120C; **Taxon:** scientificName: Euphorbiaprostrata; family: Euphorbiaceae; taxonRank: species; **Location:** continent: Europe; country: Hungary; county: Budapest; municipality: Budapest XIV.; locality: Termál street; decimalLatitude: 47.520949; decimalLongitude: 19.126483; **Identification:** identifiedBy: Attila Rigó; **Event:** eventDate: 10/09/2022; habitat: crevices in pavement
- **Type status:** Other material. **Occurrence:** recordedBy: Attila Rigó; individualCount: 300; reproductiveCondition: fruit-bearing; occurrenceID: 70034AAA-1535-5886-98D7-2FF07C4C68D0; **Taxon:** scientificName: Euphorbiaprostrata; family: Euphorbiaceae; taxonRank: species; **Location:** continent: Europe; country: Hungary; county: Budapest; municipality: Budapest III.; locality: Árpád street; decimalLatitude: 47.591159; decimalLongitude: 19.052656; **Identification:** identifiedBy: Attila Rigó; **Event:** eventDate: 28/09/2022; habitat: lawn, crevices of pavement, disturbed ground, flower boxes
- **Type status:** Other material. **Occurrence:** recordedBy: Attila Rigó; individualCount: 30; reproductiveCondition: fruit-bearing; occurrenceID: 5ABBEEF3-411E-5E59-98F6-4C817EB6B9BE; **Taxon:** scientificName: Euphorbiaprostrata; family: Euphorbiaceae; taxonRank: species; **Location:** continent: Europe; country: Hungary; county: Budapest; municipality: Budapest VI.; locality: Millenium roof garden; decimalLatitude: 47.512361; decimalLongitude: 19.059076; **Identification:** identifiedBy: Attila Rigó; **Event:** eventDate: 17/10/2022; habitat: crevices in pavement

#### Notes

Originates from North America, but a widespread alien throughout the world, especially as an urban weed (Molnár et al. 2020). First found in Hungary near Szeged (southern Hungary) (Bátori et al. 2012), but recently it was found in the urban flora of many Hungarian cities and towns. Widespread in Budapest. Studies suggest that this species is a stowaway of the ornamental plant trade (Molnár et al. 2020).

### Euphorbia serpens

Kunth 1817

CD2799F0-CAE0-5425-9BEE-F56CF81BAB3D

#### Materials

- **Type status:** Other material. **Occurrence:** recordedBy: Attila Rigó; individualCount: 40; reproductiveCondition: fruit-bearing; occurrenceID: D3B1BBF3-5182-5955-845E-B19A8FCFACF4; **Taxon:** scientificName: Euphorbiaserpens; family: Euphorbiaceae; taxonRank: species; **Location:** continent: Europe; country: Hungary; county: Budapest; municipality: Budapest III.; locality: Ezüsthegy street; decimalLatitude: 47.601214; decimalLongitude: 19.047174; **Identification:** identifiedBy: Attila Rigó; **Event:** eventDate: 07/07/2022; habitat: crevices of pavement
- **Type status:** Other material. **Occurrence:** recordedBy: Attila Rigó; individualCount: 80; reproductiveCondition: fruit-bearing; occurrenceID: E92BE988-D189-5525-AFC6-5B9B4A5B031F; **Taxon:** scientificName: Euphorbiaserpens; family: Euphorbiaceae; taxonRank: species; **Location:** continent: Europe; country: Hungary; county: Budapest; municipality: Budapest III.; locality: Frigyes street; decimalLatitude: 47.593848; decimalLongitude: 19.064482; **Identification:** identifiedBy: Attila Rigó; **Event:** eventDate: 19/07/2022; habitat: crevices of pavement
- **Type status:** Other material. **Occurrence:** recordedBy: Attila Rigó; individualCount: 40; reproductiveCondition: fruit-bearing; occurrenceID: C78A5521-7AA0-5CD3-AEA2-AF1983142187; **Taxon:** scientificName: Euphorbiaserpens; family: Euphorbiaceae; taxonRank: species; **Location:** continent: Europe; country: Hungary; county: Budapest; municipality: Budapest XIV.; locality: Zalán street; decimalLatitude: 47.504507; decimalLongitude: 19.143548; **Identification:** identifiedBy: Attila Rigó; **Event:** eventDate: 31/07/2022; habitat: crevices of pavement in garden store
- **Type status:** Other material. **Occurrence:** recordedBy: Attila Rigó; individualCount: 100; reproductiveCondition: fruit-bearing; occurrenceID: A4B83F46-9431-5A0B-86E1-21713CC601A7; **Taxon:** scientificName: Euphorbiaserpens; family: Euphorbiaceae; taxonRank: species; **Location:** continent: Europe; country: Hungary; county: Budapest; municipality: Budapest III.; locality: Árpád street; decimalLatitude: 47.590572; decimalLongitude: 19.051222; **Identification:** identifiedBy: Attila Rigó; **Event:** eventDate: 28/09/2022; habitat: crevices of pavement in garden store

#### Notes

Native to North America, but a widespread urbanophil alien throughout the world (Takács et al. 2020). In Hungary, it was first found in 2013 in Veszprém (western Hungary) (Wolf and Király 2014). Occurs rarely in the urban flora of Budapest, especially in garden stores. Studies suggest that this species is a stowaway of the ornamental plant trade (Takács et al. 2020).

### Ficus carica

L. 1753

0C4D991A-5597-5381-8F4E-29CCD6CBFF82

#### Materials

- **Type status:** Other material. **Occurrence:** recordedBy: Attila Rigó; individualCount: 1; reproductiveCondition: non-reproductive; occurrenceID: 9533AAE4-61EE-5E97-983D-79DDD91A2609; **Taxon:** scientificName: Ficuscarica; family: Moraceae; taxonRank: species; **Location:** continent: Europe; country: Hungary; county: Budapest; municipality: Budapest III.; locality: Szindbád street; decimalLatitude: 47.594881; decimalLongitude: 19.050861; **Identification:** identifiedBy: Attila Rigó; **Event:** eventDate: 24/10/2019; habitat: crack in road pavement

#### Notes

Native to Southeast Asia and the eastern Mediterranean and cultivated worldwide for its fruit and as a medicinal and ornamental plant (Mawa et al. 2013). In Hungary, it became locally naturalised in the area of Pécs (southern Hungary) and is spreading to the north (Wirth et al. 2020b). This is its first documented spontaneous occurrence in Budapest.

### Glebionis coronaria

(L.) Cass. ex Spach 1841

9BD88469-E024-5EDD-9009-190A8E08308E

#### Materials

- **Type status:** Other material. **Occurrence:** recordedBy: Attila Rigó; individualCount: 1; reproductiveCondition: in bloom; occurrenceID: 5C99E75A-E334-529A-864E-6EEC0020FE50; **Taxon:** scientificName: Glebioniscoronaria; family: Asteraceae; taxonRank: species; **Location:** continent: Europe; country: Hungary; county: Budapest; municipality: Budapest VI.; locality: Eiffel square; decimalLatitude: 47.510399; decimalLongitude: 19.059088; **Identification:** identifiedBy: Attila Rigó; **Event:** eventDate: 29/08/2019; habitat: disturbed ground

#### Notes

A Mediterranean plant used as a vegetable, a medicinal plant and an ornamental plant, which became naturalised in almost every continent (Ivashchenko 2019). It had no recent records from Hungary.

### Heliopsis helianthoides

Sweet 1826

7E2B1AF2-87C4-55A7-AD0B-DFCB3F0B9626

#### Materials

- **Type status:** Other material. **Occurrence:** recordedBy: Attila Rigó; individualCount: 3; reproductiveCondition: in bloom; occurrenceID: EC744421-360F-5873-AAC5-24758CAD0711; **Taxon:** scientificName: Heliopsishelianthoides; family: Asteraceae; taxonRank: species; **Location:** continent: Europe; country: Hungary; county: Budapest; municipality: Budapest XVIII.; locality: Szövet street; decimalLatitude: 47.398211; decimalLongitude: 19.189528; **Identification:** identifiedBy: Attila Rigó; **Event:** eventDate: 11/07/2022; habitat: crack at the base of a wall
- **Type status:** Other material. **Occurrence:** recordedBy: Attila Rigó; individualCount: 12; reproductiveCondition: non-reproductive; occurrenceID: E3221C9F-EC64-579E-883B-34E14E15129E; **Taxon:** scientificName: Heliopsishelianthoides; family: Asteraceae; taxonRank: species; **Location:** continent: Europe; country: Hungary; county: Budapest; municipality: Budapest XVII.; locality: Lemberg street; decimalLatitude: 47.483932; decimalLongitude: 19.278160; **Identification:** identifiedBy: Attila Rigó; **Event:** eventDate: 24/07/2022; habitat: crack at the base of a wall
- **Type status:** Other material. **Occurrence:** recordedBy: Attila Rigó; individualCount: 2; reproductiveCondition: in bloom; occurrenceID: 19B9753B-AE42-58B3-8AFB-1A8E5F7CFDB5; **Taxon:** scientificName: Heliopsishelianthoides; family: Asteraceae; taxonRank: species; **Location:** continent: Europe; country: Hungary; county: Budapest; municipality: Budapest XVII.; locality: Nyitány street; decimalLatitude: 47.484500; decimalLongitude: 19.286216; **Identification:** identifiedBy: Attila Rigó; **Event:** eventDate: 24/07/2022; habitat: road verge, crevices of pavement
- **Type status:** Other material. **Occurrence:** recordedBy: Attila Rigó; individualCount: 2; reproductiveCondition: in bloom; occurrenceID: FC593684-8B53-56FE-9984-7FB18F75019F; **Taxon:** scientificName: Heliopsishelianthoides; family: Asteraceae; taxonRank: species; **Location:** continent: Europe; country: Hungary; county: Budapest; municipality: Budapest XIV.; locality: Gödöllői street; decimalLatitude: 47.520879; decimalLongitude: 19.141696; **Identification:** identifiedBy: Attila Rigó; **Event:** eventDate: 10/09/2022; habitat: crevices of pavement, urban hedge
- **Type status:** Other material. **Occurrence:** recordedBy: Attila Rigó; individualCount: 15; reproductiveCondition: fruit-bearing; occurrenceID: 949D8E52-6A24-5820-A0A7-2137DB6486F6; **Taxon:** scientificName: Heliopsishelianthoides; family: Asteraceae; taxonRank: species; **Location:** continent: Europe; country: Hungary; county: Budapest; municipality: Budapest XIX.; locality: Corvin boulevard; decimalLatitude: 47.458214; decimalLongitude: 19.124556; **Identification:** identifiedBy: Attila Rigó; **Event:** eventDate: 17/09/2022; habitat: dried-up ditch
- **Type status:** Other material. **Occurrence:** recordedBy: Attila Rigó; individualCount: 10; reproductiveCondition: fruit-bearing; occurrenceID: DA6B8FF4-100D-5D4D-89DC-EB5BC4A5AA28; **Taxon:** scientificName: Heliopsishelianthoides; family: Asteraceae; taxonRank: species; **Location:** continent: Europe; country: Hungary; county: Budapest; municipality: Budapest XIX.; locality: Hunyadi street; decimalLatitude: 47.448518; decimalLongitude: 19.130056; **Identification:** identifiedBy: Attila Rigó; **Event:** eventDate: 17/09/2022; habitat: crack at the base of a wall, crevices of pavement

#### Notes

Native to North America and cultivated as an ornamental in Europe. It occurs as a casual alien in European countries (Nāburga and Evarts-Bunders 2019). Missing from the most recent alien list of Hungary (Csiky et al. 2023), but has some records from the country (András Schmotzer ex litt.). New to Budapest.

### Lagenaria siceraria

(Molina) Standl. 1930

A42E8576-4732-504C-A91D-2F3235D45734

#### Materials

- **Type status:** Other material. **Occurrence:** recordedBy: Attila Rigó; individualCount: 3; reproductiveCondition: in bloom; occurrenceID: 7D64ADCF-67CD-58A8-BB8C-6B15C7BF7642; **Taxon:** scientificName: Lagenariasiceraria; family: Cucurbitaceae; taxonRank: species; **Location:** continent: Europe; country: Hungary; county: Budapest; municipality: Budapest XI.; locality: Mesterházi street; decimalLatitude: 47.451981; decimalLongitude: 19.041545; **Identification:** identifiedBy: Attila Rigó; **Event:** eventDate: 22/10/2022; habitat: gravelly road verge

#### Notes

Native to tropical Africa and cultivated as a vegetable in tropical and subtropical regions and sometimes in temperate regions, but only recently recorded as a casual garden escapee (Gudžinskas 2017). Balogh et al. (2004) listed it as a casual neophyte in Hungary, but it had no recent records from the country.

### Lepidium didymum

L. 1767

D088B1E8-885A-58E7-8B51-DD85C4AD6367

#### Materials

- **Type status:** Other material. **Occurrence:** recordedBy: Attila Rigó; individualCount: 2; reproductiveCondition: fruit-bearing; occurrenceID: 1B19905B-5287-5993-A5B8-014B7E50D4EC; **Taxon:** scientificName: Lepidiumdidymum; family: Brassicaceae; taxonRank: species; **Location:** continent: Europe; country: Hungary; county: Budapest; municipality: Budapest XIV.; locality: roof of the Museum of Etnography; decimalLatitude: 47.510830; decimalLongitude: 19.082220; **Identification:** identifiedBy: Attila Rigó; **Event:** eventDate: 02/06/2022; habitat: gravelly flower bed
- **Type status:** Other material. **Occurrence:** recordedBy: Attila Rigó; individualCount: 4; reproductiveCondition: fruit-bearing; occurrenceID: F562EA7E-4A84-5C9D-8C31-CB610E59F7C3; **Taxon:** scientificName: Lepidiumdidymum; family: Brassicaceae; taxonRank: species; **Location:** continent: Europe; country: Hungary; county: Budapest; municipality: Budapest XIV.; locality: Zalán street; decimalLatitude: 47.504850; decimalLongitude: 19.144037; **Identification:** identifiedBy: Attila Rigó; **Event:** eventDate: 10/06/2022; habitat: irrigated flower pot in gardening store

#### Notes

A plant of uncertain origin which is a widespread weed in western and southern Europe, but a rare casual alien in Central Europe (Schmidt 2020). There are very few recent records of this species from Hungary. New to Budapest.

### Linaria maroccana

Hook.f. 1872

637EB3D3-4ABD-5E81-8D8E-C8F9435DF95E

#### Materials

- **Type status:** Other material. **Occurrence:** recordedBy: Attila Rigó; individualCount: 1; reproductiveCondition: in bloom; occurrenceID: 7D312410-B935-55B7-B40C-47A4E71B8FB4; **Taxon:** scientificName: Linariamaroccana; family: Plantaginaceae; taxonRank: species; **Location:** continent: Europe; country: Hungary; county: Budapest; municipality: Budapest VIII.; locality: Villám street; decimalLatitude: 47.479347; decimalLongitude: 19.099769; **Identification:** identifiedBy: Attila Rigó; **Event:** eventDate: 27/06/2019; habitat: crack in road pavement
- **Type status:** Other material. **Occurrence:** recordedBy: Attila Rigó; individualCount: 4; reproductiveCondition: fruit-bearing; occurrenceID: A283E098-1676-5BD5-A42E-574203500A51; **Taxon:** scientificName: Linariamaroccana; family: Plantaginaceae; taxonRank: species; **Location:** continent: Europe; country: Hungary; county: Budapest; municipality: Budapest VI.; locality: Eiffel square; decimalLatitude: 47.510399; decimalLongitude: 19.059088; **Identification:** identifiedBy: Attila Rigó; **Event:** eventDate: 29/08/2019; habitat: disturbed ground

#### Notes

An endemic plant of Morocco that is cultivated as an ornamental in many countries (Verloove and Sánchez Gullón 2012). Appears as a casual alien in Europe, for example, Spain (Verloove and Sánchez Gullón 2012) and Czechia (Kocián 2014), but also in North America (Poindexter et al. 2011). New casual alien to Hungary.

### Matthiola longipetala subsp. bicornis

Ball 1963

7310B88C-B0C0-5F21-BFFC-D9136DBD90B3

#### Materials

- **Type status:** Other material. **Occurrence:** recordedBy: Attila Rigó; individualCount: 2; reproductiveCondition: in bloom; occurrenceID: 9A13D87F-E2EC-5C14-9CD3-493016032D53; **Taxon:** scientificName: Matthiola Matthiolalongipetalasubsp.bicornis; family: Brassicaceae; taxonRank: subspecies; **Location:** continent: Europe; country: Hungary; county: Budapest; municipality: Budapest VII.; locality: Verseny street; decimalLatitude: 47.501992; decimalLongitude: 19.089403; **Identification:** identifiedBy: Attila Rigó; **Event:** eventDate: 30/05/2022; habitat: disturbed ground in vacant ground-plot

#### Notes

Native to the Mediterranean and used as an ornamental (Király 2009). An occasional garden escapee in Europe, for example, Ukraine (Mosyakin and Yavorska 2002). It has two recent records from Hungary (Schmotzer 2015 and Király and Király 2018). New to Budapest.

### Nassella tenuissima

(Trin.) Barkworth 1990

20EEF338-E7C9-5F41-A489-7E6FEDC821DB

#### Materials

- **Type status:** Other material. **Occurrence:** recordedBy: Attila Rigó; individualCount: 2; reproductiveCondition: fruit-bearing; occurrenceID: 6969F020-1A53-5927-BDFC-8299D9D0CA0E; **Taxon:** scientificName: Nassellatenuissima; family: Poaceae; taxonRank: species; **Location:** continent: Europe; country: Hungary; county: Budapest; municipality: Budapest XIV.; locality: Zalán street; decimalLatitude: 47.504780; decimalLongitude: 19.145875; **Identification:** identifiedBy: Attila Rigó; **Event:** eventDate: 10/06/2022; habitat: crevices in pavement
- **Type status:** Other material. **Occurrence:** recordedBy: Attila Rigó; individualCount: 6; reproductiveCondition: non-reproductive; occurrenceID: 9842E4E3-DE07-5E26-8897-60E6651A2EB3; **Taxon:** scientificName: Nassellatenuissima; family: Poaceae; taxonRank: species; **Location:** continent: Europe; country: Hungary; county: Budapest; municipality: Budapest XIII.; locality: Szent István park; decimalLatitude: 47.518783; decimalLongitude: 19.050875; **Identification:** identifiedBy: Attila Rigó; **Event:** eventDate: 24/06/2022; habitat: crevices in pavement
- **Type status:** Other material. **Occurrence:** recordedBy: Attila Rigó; individualCount: 1; reproductiveCondition: non-reproductive; occurrenceID: 307ABFFB-ED7B-51D0-841A-4AEA9ACA01C9; **Taxon:** scientificName: Nassellatenuissima; family: Poaceae; taxonRank: species; **Location:** continent: Europe; country: Hungary; county: Budapest; municipality: Budapest II.; locality: Szabó Magda promenade; decimalLatitude: 47.509689; decimalLongitude: 19.028398; **Identification:** identifiedBy: Attila Rigó; **Event:** eventDate: 05/10/2022; habitat: crevices in pavement
- **Type status:** Other material. **Occurrence:** recordedBy: Attila Rigó; individualCount: 5; reproductiveCondition: non-reproductive; occurrenceID: A19B6A06-34DF-54F5-9450-D2F6FFE865FF; **Taxon:** scientificName: Nassellatenuissima; family: Poaceae; taxonRank: species; **Location:** continent: Europe; country: Hungary; county: Budapest; municipality: Budapest I.; locality: Batthány square; decimalLatitude: 47.506133; decimalLongitude: 19.038567; **Identification:** identifiedBy: Attila Rigó; **Event:** eventDate: 17/11/2022; habitat: crevices in pavement

#### Notes

A grass native to North America that is used as an ornamental worldwide. Naturalised in New Zealand, Australia and South Africa. According to recent reports, it has become a casual alien in western Europe (Álvarez et al. 2016). In recent years, this plant was found in Hungary as a casual alien as well (Wirth et al. 2020a and Molnár et al. 2022). New to Budapest.

### Nepeta racemosa

Bergmans ex Stearn 1939

E3681234-BAD8-588B-B648-EAF6784FCB68

#### Materials

- **Type status:** Other material. **Occurrence:** recordedBy: Attila Rigó; individualCount: 3; reproductiveCondition: non-reproductive; occurrenceID: 3BF389BB-6E45-5AB1-89E8-20DB1C883608; **Taxon:** scientificName: Nepetaracemosa; family: Lamiaceae; taxonRank: species; **Location:** continent: Europe; country: Hungary; county: Budapest; municipality: Budapest XI.; locality: Balogh street; decimalLatitude: 47.479351; decimalLongitude: 19.042380; **Identification:** identifiedBy: Attila Rigó; **Event:** eventDate: 29/03/2022; habitat: crevices in pavement
- **Type status:** Other material. **Occurrence:** recordedBy: Attila Rigó; individualCount: 6; reproductiveCondition: in bloom; occurrenceID: E21F524B-8D39-507E-8BA9-6A1A7701E3A6; **Taxon:** scientificName: Nepetaracemosa; family: Lamiaceae; taxonRank: species; **Location:** continent: Europe; country: Hungary; county: Budapest; municipality: Budapest XII.; locality: Győri road; decimalLatitude: 47.493967; decimalLongitude: 19.026843; **Identification:** identifiedBy: Attila Rigó; **Event:** eventDate: 13/05/2022; habitat: lawn
- **Type status:** Other material. **Occurrence:** recordedBy: Attila Rigó; individualCount: 12; reproductiveCondition: fruit-bearing; occurrenceID: C1330943-39DE-5569-9746-ECC050D76644; **Taxon:** scientificName: Nepetaracemosa; family: Lamiaceae; taxonRank: species; **Location:** continent: Europe; country: Hungary; county: Budapest; municipality: Budapest XIII.; locality: Árpád bridge; decimalLatitude: 47.532775; decimalLongitude: 19.065995; **Identification:** identifiedBy: Attila Rigó; **Event:** eventDate: 20/05/2022; habitat: lawn
- **Type status:** Other material. **Occurrence:** recordedBy: Attila Rigó; individualCount: 40; reproductiveCondition: fruit-bearing; occurrenceID: 9C52599D-5CC5-5C75-9504-D7C71BA942E9; **Taxon:** scientificName: Nepetaracemosa; family: Lamiaceae; taxonRank: species; **Location:** continent: Europe; country: Hungary; county: Budapest; municipality: Budapest II.; locality: Mansfeld Péter park; decimalLatitude: 47.516809; decimalLongitude: 19.032353; **Identification:** identifiedBy: Attila Rigó; **Event:** eventDate: 27/05/2022; habitat: lawn, crevices in road pavement
- **Type status:** Other material. **Occurrence:** recordedBy: Attila Rigó; individualCount: 12; reproductiveCondition: non-reproductive; occurrenceID: 19675DEE-17D1-5D84-93B0-39ABB5DC9486; **Taxon:** scientificName: Nepetaracemosa; family: Lamiaceae; taxonRank: species; **Location:** continent: Europe; country: Hungary; county: Budapest; municipality: Budapest V.; locality: Erzsébet square; decimalLatitude: 47.498292; decimalLongitude: 19.051708; **Identification:** identifiedBy: Attila Rigó; **Event:** eventDate: 04/06/2022; habitat: crevices in pavement
- **Type status:** Other material. **Occurrence:** recordedBy: Attila Rigó; individualCount: 10; reproductiveCondition: non-reproductive; occurrenceID: 03A476A7-93A2-534D-96E6-66AA50323701; **Taxon:** scientificName: Nepetaracemosa; family: Lamiaceae; taxonRank: species; **Location:** continent: Europe; country: Hungary; county: Budapest; municipality: Budapest V.; locality: Kossuth square; decimalLatitude: 47.505951; decimalLongitude: 19.046678; **Identification:** identifiedBy: Attila Rigó; **Event:** eventDate: 04/06/2022; habitat: flower bed
- **Type status:** Other material. **Occurrence:** recordedBy: Attila Rigó; individualCount: 30; reproductiveCondition: fruit-bearing; occurrenceID: 6274AA8F-1134-5908-A8CB-5A74E81F9E2C; **Taxon:** scientificName: Nepetaracemosa; family: Lamiaceae; taxonRank: species; **Location:** continent: Europe; country: Hungary; county: Budapest; municipality: Budapest XIV.; locality: Örs Vezér square; decimalLatitude: 47.504781; decimalLongitude: 19.138095; **Identification:** identifiedBy: Attila Rigó; **Event:** eventDate: 10/06/2022; habitat: urban hedge, flower bed
- **Type status:** Other material. **Occurrence:** recordedBy: Attila Rigó; individualCount: 5; reproductiveCondition: fruit-bearing; occurrenceID: F7E60954-EF29-56ED-881F-3B798061615A; **Taxon:** scientificName: Nepetaracemosa; family: Lamiaceae; taxonRank: species; **Location:** continent: Europe; country: Hungary; county: Budapest; municipality: Budapest II.; locality: Temető street; decimalLatitude: 47.566199; decimalLongitude: 18.956917; **Identification:** identifiedBy: Attila Rigó; **Event:** eventDate: 12/06/2022; habitat: crevices in pavement
- **Type status:** Other material. **Occurrence:** recordedBy: Attila Rigó; individualCount: 12; reproductiveCondition: in bloom; occurrenceID: 1E6A7DE7-7B5A-54A4-8297-162C073B367B; **Taxon:** scientificName: Nepetaracemosa; family: Lamiaceae; taxonRank: species; **Location:** continent: Europe; country: Hungary; county: Budapest; municipality: Budapest XIII.; locality: Béke square; decimalLatitude: 47.530358; decimalLongitude: 19.082712; **Identification:** identifiedBy: Attila Rigó; **Event:** eventDate: 13/06/2022; habitat: crevices in pavement
- **Type status:** Other material. **Occurrence:** recordedBy: Attila Rigó; individualCount: 10; reproductiveCondition: in bloom; occurrenceID: 3C98F0BD-0AA8-5CE3-AB18-762234B85C95; **Taxon:** scientificName: Nepetaracemosa; family: Lamiaceae; taxonRank: species; **Location:** continent: Europe; country: Hungary; county: Budapest; municipality: Budapest XIII.; locality: Pozsonyi road; decimalLatitude: 47.514414; decimalLongitude: 19.048822; **Identification:** identifiedBy: Attila Rigó; **Event:** eventDate: 24/06/2022; habitat: flower bed
- **Type status:** Other material. **Occurrence:** recordedBy: Attila Rigó; individualCount: 3; reproductiveCondition: non-reproductive; occurrenceID: B624C06B-39C4-5E87-92B4-A905ACCC5CA1; **Taxon:** scientificName: Nepetaracemosa; family: Lamiaceae; taxonRank: species; **Location:** continent: Europe; country: Hungary; county: Budapest; municipality: Budapest IV.; locality: Wolfner street; decimalLatitude: 47.563286; decimalLongitude: 19.079628; **Identification:** identifiedBy: Attila Rigó; **Event:** eventDate: 06/09/2022; habitat: crevices in pavement
- **Type status:** Other material. **Occurrence:** recordedBy: Attila Rigó; individualCount: 12; reproductiveCondition: non-reproductive; occurrenceID: A498704E-EF0C-56C0-860E-291F76AE38C5; **Taxon:** scientificName: Nepetaracemosa; family: Lamiaceae; taxonRank: species; **Location:** continent: Europe; country: Hungary; county: Budapest; municipality: Budapest XVI.; locality: Cica street; decimalLatitude: 47.526713; decimalLongitude: 19.223969; **Identification:** identifiedBy: Attila Rigó; **Event:** eventDate: 07/09/2022; habitat: dried-up ditch, road verge

#### Notes

An ornamental and medicinal plant that originates from the Caucasus. It has some old and recent records from Hungary (Király and Király 2018). Balogh et al. (2004) lists this species as a casual neophyte, but it is missing from the list of Csiky et al. (2023). We suggest that this plant is locally naturalised in Budapest. *Nepetafaasenii* Bergmans ex Stearn in Rigó and Barina (2020) was erroneously reported, all of its records refer to *Nepetaracemosa* Bergmans ex Stearn.

### Nicotiana alata

Link & Otto 1830

C3796847-5814-5602-8A04-76A30486E5AA

#### Materials

- **Type status:** Other material. **Occurrence:** recordedBy: Attila Rigó; individualCount: 13; reproductiveCondition: in bloom; occurrenceID: 0CF8F9F7-C4B1-5BE5-92BB-075E45375BD8; **Taxon:** scientificName: Nicotianaalata; family: Solanaceae; taxonRank: species; **Location:** continent: Europe; country: Hungary; county: Budapest; municipality: Budapest IX.; locality: Szent István Hospital; decimalLatitude: 47.477710; decimalLongitude: 19.088893; **Identification:** identifiedBy: Attila Rigó; **Event:** eventDate: 25/09/2022; habitat: flower bed

#### Notes

A South American plant that is cultivated as an ornamental (Király 2009). It appears as an ephemeral casual alien in some European countries, for example, Romania (Anastasiu et al. 2009). Balogh et al. (2004) and Csiky et al. (2023) list it as a casual alien. This species has two recent records from Hungary (Wirth et al. 2020a), neither of which is from Budapest.

### Nicotiana sylvestris

Speg. 1898

4874112A-9816-50A5-9E03-2EFD44D0C5BC

#### Materials

- **Type status:** Other material. **Occurrence:** recordedBy: Attila Rigó; individualCount: 1; reproductiveCondition: in bloom; occurrenceID: 166C8F00-EFCD-5289-916F-B51061D86C43; **Taxon:** scientificName: Nicotianasylvestris; family: Solanaceae; taxonRank: species; **Location:** continent: Europe; country: Hungary; county: Budapest; municipality: Budapest XXI.; locality: Vasút street; decimalLatitude: 47.425845; decimalLongitude: 19.062677; **Identification:** identifiedBy: Attila Rigó; **Event:** eventDate: 16/09/2022; habitat: disturbed ground

#### Notes

A rare ornamental plant of South American origin (Sierro et al. 2013). It appears as a casual alien in Belgium (Verloove 2006). New to the flora of Hungary. Probably an ephemeral alien.

### Panicum riparium

H.Scholz 2002

CAD02385-BB7F-5731-8510-727BBBBEBF40

#### Materials

- **Type status:** Other material. **Occurrence:** recordedBy: Attila Rigó; individualCount: 3; reproductiveCondition: fruit-bearing; occurrenceID: 8A24FDAB-DD17-5BA9-9420-6A1D6C3D917B; **Taxon:** scientificName: Panicumriparium; family: Poaceae; taxonRank: species; **Location:** continent: Europe; country: Hungary; county: Budapest; municipality: Budapest III.; locality: Kunigunda road; decimalLatitude: 47.564684; decimalLongitude: 19.036358; **Identification:** identifiedBy: Attila Rigó; **Event:** eventDate: 02/09/2022; habitat: dried-up ditch
- **Type status:** Other material. **Occurrence:** recordedBy: Attila Rigó; individualCount: 20; reproductiveCondition: fruit-bearing; occurrenceID: F25194E2-CAED-5467-B9D7-47249F1B58E6; **Taxon:** scientificName: Panicumriparium; family: Poaceae; taxonRank: species; **Location:** continent: Europe; country: Hungary; county: Budapest; municipality: Budapest XI.; locality: Gazdagréti road; decimalLatitude: 47.473155; decimalLongitude: 18.99201; **Identification:** identifiedBy: Attila Rigó; **Event:** eventDate: 18/09/2022; habitat: crevices of pavement

#### Notes

An overlooked, long established, but data-poor taxon described from Europe by H. Scholz in 2002, but it originates from North America. Recently, it was recognised and recorded from Central European countries (Scholz 2002 and Király and Alegro 2015). It had some previous records from Hungary (e.g. Schmidt 2015) and one record from Budapest (Dávid Schmidt ined.).

### Panicum virgatum

L. 1753

804BC948-6824-5CBE-99B9-4AE46940EA63

#### Materials

- **Type status:** Other material. **Occurrence:** recordedBy: Attila Rigó; individualCount: 2; reproductiveCondition: in bloom; occurrenceID: 7FF2EFD8-F686-56AD-B140-2F2C74B13578; **Taxon:** scientificName: Panicumvirgatum; family: Poaceae; taxonRank: species; **Location:** continent: Europe; country: Hungary; county: Budapest; municipality: Budapest III.; locality: Holdudvar park; decimalLatitude: 47.546522; decimalLongitude: 19.030164; **Identification:** identifiedBy: Attila Rigó; **Event:** eventDate: 23/09/2022; habitat: crack at the base of a wall, lawn
- **Type status:** Other material. **Occurrence:** recordedBy: Zoltán Barina; individualCount: 1; reproductiveCondition: in bloom; occurrenceID: 808EE2B3-E3C0-5AE5-8F9C-FC8FAECB740F; **Taxon:** scientificName: Panicumvirgatum; family: Poaceae; taxonRank: species; **Location:** continent: Europe; country: Hungary; county: Budapest; municipality: Budapest IX.; locality: Kvassay bridge; decimalLatitude: 47.480768; decimalLongitude: 19.072170; **Identification:** identifiedBy: Zoltán Barina; **Event:** eventDate: 01/10/2022; habitat: crevices of road pavement

#### Notes

A large perennial grass native to North America which is used as an ornamental in several countries and appears as a garden escapee in many European countries (Eliáš Jr. et al. 2023). In Slovakia, the species is naturalised and could potentially become invasive (Eliáš Jr. et al. 2023). In Hungary, it had one previous record (Wirth et al. 2020a). New to Budapest.

### Perovskia atriplicifolia

Benth. 1848

25E1A297-6C96-545D-9FB6-59D0DD2E2D9D

#### Materials

- **Type status:** Other material. **Occurrence:** recordedBy: Attila Rigó; individualCount: 20; reproductiveCondition: non-reproductive; occurrenceID: BFC960E9-035B-513C-8C2E-D26D11DC1E77; **Taxon:** scientificName: Perovskiaatriplicifolia; family: Lamiaceae; taxonRank: species; **Location:** continent: Europe; country: Hungary; county: Budapest; municipality: Budapest V.; locality: Erzsébet square; decimalLatitude: 47.498473; decimalLongitude: 19.051634; **Identification:** identifiedBy: Attila Rigó; **Event:** eventDate: 04/06/2022; habitat: crevices of pavement, front garden
- **Type status:** Other material. **Occurrence:** recordedBy: Attila Rigó, Ákos Malatinszky; individualCount: 3; reproductiveCondition: non-reproductive; occurrenceID: A7744EC1-A858-5832-8A82-EEF0360F6DB3; **Taxon:** scientificName: Perovskiaatriplicifolia; family: Lamiaceae; taxonRank: species; **Location:** continent: Europe; country: Hungary; county: Budapest; municipality: Budapest XIII.; locality: Gömb street; decimalLatitude: 47.532243; decimalLongitude: 19.073084; **Identification:** identifiedBy: Attila Rigó; **Event:** eventDate: 13/06/2022; habitat: front garden
- **Type status:** Other material. **Occurrence:** recordedBy: Attila Rigó; individualCount: 20; reproductiveCondition: non-reproductive; occurrenceID: 0A030E54-61C1-50AC-BB85-56F812586F49; **Taxon:** scientificName: Perovskiaatriplicifolia; family: Lamiaceae; taxonRank: species; **Location:** continent: Europe; country: Hungary; county: Budapest; municipality: Budapest VIII.; locality: II. János Pál Pápa square; decimalLatitude: 47.497110; decimalLongitude: 19.078076; **Identification:** identifiedBy: Attila Rigó; **Event:** eventDate: 17/06/2022; habitat: lawn, flower bed
- **Type status:** Other material. **Occurrence:** recordedBy: Attila Rigó; individualCount: 5; reproductiveCondition: non-reproductive; occurrenceID: CE9B1F25-EBE0-5155-994C-BFDF9B89542A; **Taxon:** scientificName: Perovskiaatriplicifolia; family: Lamiaceae; taxonRank: species; **Location:** continent: Europe; country: Hungary; county: Budapest; municipality: Budapest XI.; locality: Bukarest street; decimalLatitude: 47.474532; decimalLongitude: 19.040319; **Identification:** identifiedBy: Attila Rigó; **Event:** eventDate: 22/06/2022; habitat: front garden
- **Type status:** Other material. **Occurrence:** recordedBy: Attila Rigó; individualCount: 3; reproductiveCondition: in bloom; occurrenceID: 9BA02F4D-A6E6-5A9B-AFE7-5BB4FF923D7E; **Taxon:** scientificName: Perovskiaatriplicifolia; family: Lamiaceae; taxonRank: species; **Location:** continent: Europe; country: Hungary; county: Budapest; municipality: Budapest II.; locality: Aranka street; decimalLatitude: 47.514517; decimalLongitude: 19.021874; **Identification:** identifiedBy: Attila Rigó; **Event:** eventDate: 06/07/2022; habitat: flower bed
- **Type status:** Other material. **Occurrence:** recordedBy: Attila Rigó; individualCount: 20; reproductiveCondition: non-reproductive; occurrenceID: 22812CF3-8058-5F7F-92C2-00E9C21ABFA3; **Taxon:** scientificName: Perovskiaatriplicifolia; family: Lamiaceae; taxonRank: species; **Location:** continent: Europe; country: Hungary; county: Budapest; municipality: Budapest II.; locality: Millenáris park; decimalLatitude: 47.510363; decimalLongitude: 19.026510; **Identification:** identifiedBy: Attila Rigó; **Event:** eventDate: 06/07/2022; habitat: flower bed
- **Type status:** Other material. **Occurrence:** recordedBy: Attila Rigó; individualCount: 5; reproductiveCondition: fruit-bearing; occurrenceID: 9990069D-0561-599A-B807-817E3CD7936C; **Taxon:** scientificName: Perovskiaatriplicifolia; family: Lamiaceae; taxonRank: species; **Location:** continent: Europe; country: Hungary; county: Budapest; municipality: Budapest XXI.; locality: Szent Imre tér; decimalLatitude: 47.431425; decimalLongitude: 19.068341; **Identification:** identifiedBy: Attila Rigó; **Event:** eventDate: 04/09/2022; habitat: urban hedge
- **Type status:** Other material. **Occurrence:** recordedBy: Attila Rigó; individualCount: 10; reproductiveCondition: fruit-bearing; occurrenceID: 9C25CFE3-F735-52FD-B7F5-A2D0E9021142; **Taxon:** scientificName: Perovskiaatriplicifolia; family: Lamiaceae; taxonRank: species; **Location:** continent: Europe; country: Hungary; county: Budapest; municipality: Budapest XIV.; locality: Patakpart park; decimalLatitude: 47.522839; decimalLongitude: 19.128744; **Identification:** identifiedBy: Attila Rigó; **Event:** eventDate: 10/09/2022; habitat: disturbed ground, flower bed
- **Type status:** Other material. **Occurrence:** recordedBy: Attila Rigó; individualCount: 12; reproductiveCondition: non-reproductive; occurrenceID: 29676F87-FE72-51D1-8FB2-6F25D87F5B7E; **Taxon:** scientificName: Perovskiaatriplicifolia; family: Lamiaceae; taxonRank: species; **Location:** continent: Europe; country: Hungary; county: Budapest; municipality: Budapest XI.; locality: Ratkóc street; decimalLatitude: 47.471512; decimalLongitude: 19.003734; **Identification:** identifiedBy: Attila Rigó; **Event:** eventDate: 18/09/2022; habitat: urban hedge
- **Type status:** Other material. **Occurrence:** recordedBy: Attila Rigó; individualCount: 30; reproductiveCondition: non-reproductive; occurrenceID: 000AE938-60D3-5261-A606-FB83BFEFDB55; **Taxon:** scientificName: Perovskiaatriplicifolia; family: Lamiaceae; taxonRank: species; **Location:** continent: Europe; country: Hungary; county: Budapest; municipality: Budapest III.; locality: Holdudvar park; decimalLatitude: 47.545750; decimalLongitude: 19.031604; **Identification:** identifiedBy: Attila Rigó; **Event:** eventDate: 23/09/2022; habitat: crevices of pavement, front garden
- **Type status:** Other material. **Occurrence:** recordedBy: Attila Rigó; individualCount: 30; reproductiveCondition: non-reproductive; occurrenceID: 197B5424-E760-5442-B7DD-63BF18E82326; **Taxon:** scientificName: Perovskiaatriplicifolia; family: Lamiaceae; taxonRank: species; **Location:** continent: Europe; country: Hungary; county: Budapest; municipality: Budapest VIII.; locality: Kőbányai road; decimalLatitude: 47.484173; decimalLongitude: 19.115278; **Identification:** identifiedBy: Attila Rigó; **Event:** eventDate: 28/10/2022; habitat: crevices of pavement

#### Notes

An ornamental plant of Asian origin that appears as an alien in some European countries (Gilli et al. 2019). It had some previous records from Hungary (Szabó and Horváth 2005). We suggest that this species is locally naturalised in Budapest.

### Persicaria orientalis

(L.) Spach 1841

0DC6EAD0-5799-5221-AE33-4ED0428ECCE1

#### Materials

- **Type status:** Other material. **Occurrence:** recordedBy: Attila Rigó; individualCount: 2; reproductiveCondition: in bloom; occurrenceID: 3FFEB232-3880-5BD8-9AD0-BA0C2A421391; **Taxon:** scientificName: Persicariaorientalis; family: Polygonaceae; taxonRank: species; **Location:** continent: Europe; country: Hungary; county: Budapest; municipality: Budapest XVI.; locality: Tabódy Ida square; decimalLatitude: 47.518890; decimalLongitude: 19.230594; **Identification:** identifiedBy: Attila Rigó; **Event:** eventDate: 07/09/2022; habitat: crevices of pavement

#### Notes

Ornamental plant that originates from East Asia and appears as an alien invasive in many countries (Güneş Özkan and Yazlik 2020). In Hungary, it only has casual occurrences (Csiky et al. 2018 and Haszonits et al. 2021). New to Budapest.

### Polygonum rurivagum

Jord. ex Boreau 1857

46E74E4C-A65C-5DF7-97E4-0FA0F0479042

#### Materials

- **Type status:** Other material. **Occurrence:** recordedBy: Attila Rigó, Ákos Malatinszky; individualCount: 18; reproductiveCondition: in bloom; occurrenceID: F4222A30-BCD4-518F-8A92-ECE5C5C6E6D0; **Taxon:** scientificName: Polygonumrurivagum; family: Polygonaceae; taxonRank: species; **Location:** continent: Europe; country: Hungary; county: Budapest; municipality: Budapest IV.; locality: Szent István square; decimalLatitude: 47.562156; decimalLongitude: 19.088614; **Identification:** identifiedBy: Attila Rigó; **Event:** eventDate: 06/09/2022; habitat: flower bed

#### Notes

An archaeophyte that belongs to the *Polygonumaviculare* L. complex (Király 2009). The species is data-poor in Hungary and has only one recent published record (Molnár et al. 2018).

### Polypogon viridis

(Gouan) Breistr. 1966

B95E1FCB-0599-5FA1-B288-D89D88D5C64D

#### Materials

- **Type status:** Other material. **Occurrence:** recordedBy: Attila Rigó; individualCount: 8; reproductiveCondition: fruit-bearing; occurrenceID: BB81364B-1C81-51AD-95C5-A4BBB7F0CA37; **Taxon:** scientificName: Polypogonviridis; family: Poaceae; taxonRank: species; **Location:** continent: Europe; country: Hungary; county: Budapest; municipality: Budapest II.; locality: Zilah street; decimalLatitude: 47.515377; decimalLongitude: 19.010235; **Identification:** identifiedBy: Attila Rigó; **Event:** eventDate: 22/10/2022; habitat: flower bed in a garden store

#### Description

Native to the Mediterranean and was reported from many European countries as an alien. First found in Hungary in 2019 in many localities in the western part of the country (Wirth 2019). New to Budapest.

### Polystichum setiferum

Rosendahl 1916

A6A90453-4808-5C62-93AC-A3675B7A87D7

#### Materials

- **Type status:** Other material. **Occurrence:** recordedBy: Attila Rigó; individualCount: 1; reproductiveCondition: spore bearing; occurrenceID: 33E004CC-6D7A-5033-AA99-B29C42F60C58; **Taxon:** scientificName: Polystichumsetiferum; family: Dryopteridaceae; taxonRank: species; **Location:** continent: Europe; country: Hungary; county: Budapest; municipality: Budapest XXI.; locality: Hőerőmű street; decimalLatitude: 47.426974; decimalLongitude: 19.056444; **Identification:** identifiedBy: Attila Rigó; **Event:** eventDate: 16/09/2022; habitat: manhole under the influence of a broken drainage pipe

#### Notes

A native, but rare and protected fern in Hungary, with localities mainly in the western part of the country (Király 2009). The occurrence of this species in an urban environment is noteworthy.

### Pteridium aquilinum

(L.) Kuhn 1879

AEA807B8-31F5-55AB-81BF-543AD2399971

#### Materials

- **Type status:** Other material. **Occurrence:** recordedBy: Attila Rigó; individualCount: 1; reproductiveCondition: non-reproductive; occurrenceID: 43579498-892D-551E-B632-A165BCEA9422; **Taxon:** scientificName: Pteridiumaquilinum; family: Dennstaedtiaceae; taxonRank: species; **Location:** continent: Europe; country: Hungary; county: Budapest; municipality: Budapest III.; locality: Botond street; decimalLatitude: 47.5797433; decimalLongitude: 19.044764; **Identification:** identifiedBy: Attila Rigó; **Event:** eventDate: 22/09/2022; habitat: crack at the base of a wall

#### Notes

A native fern in Hungary that occurs rarely in urban environments. It has some previous records from Budapest (Tamás et al. 2017).

### Sagina apetala

Ard. 1764

922101F4-4D64-525C-90EC-9914F9CAFBEF

#### Materials

- **Type status:** Other material. **Occurrence:** recordedBy: Attila Rigó; individualCount: 5; reproductiveCondition: fruit-bearing; occurrenceID: 2E18CB62-4E22-5250-A7D7-102CF5FF39D0; **Taxon:** scientificName: Saginaapetala; family: Caryophyllaceae; taxonRank: species; **Location:** continent: Europe; country: Hungary; county: Budapest; municipality: Budapest I.; locality: Kosciuskó Tádé street; decimalLatitude: 47.499133; decimalLongitude: 19.026057; **Identification:** identifiedBy: Attila Rigó; **Event:** eventDate: 13/05/2022; habitat: crevices of pavement
- **Type status:** Other material. **Occurrence:** recordedBy: Attila Rigó; individualCount: 20; reproductiveCondition: fruit-bearing; occurrenceID: 69E2AC98-93A5-515E-84ED-8A661DD22D94; **Taxon:** scientificName: Saginaapetala; family: Caryophyllaceae; taxonRank: species; **Location:** continent: Europe; country: Hungary; county: Budapest; municipality: Budapest XI.; locality: Kelenföldi road; decimalLatitude: 47.468687; decimalLongitude: 19.023522; **Identification:** identifiedBy: Attila Rigó; **Event:** eventDate: 21/05/2022; habitat: crevices of pavement
- **Type status:** Other material. **Occurrence:** recordedBy: Attila Rigó; individualCount: 15; reproductiveCondition: fruit-bearing; occurrenceID: CB27F7FC-BBE5-5174-BB44-8671D250879F; **Taxon:** scientificName: Saginaapetala; family: Caryophyllaceae; taxonRank: species; **Location:** continent: Europe; country: Hungary; county: Budapest; municipality: Budapest IX.; locality: Közraktár street; decimalLatitude: 47.483969; decimalLongitude: 19.060879; **Identification:** identifiedBy: Attila Rigó; **Event:** eventDate: 23/05/2022; habitat: crevices of pavement
- **Type status:** Other material. **Occurrence:** recordedBy: Attila Rigó; individualCount: 15; reproductiveCondition: fruit-bearing; occurrenceID: 11FD98A7-61B2-5E4D-BD66-B8EB485EF1E4; **Taxon:** scientificName: Saginaapetala; family: Caryophyllaceae; taxonRank: species; **Location:** continent: Europe; country: Hungary; county: Budapest; municipality: Budapest IX.; locality: Kinizsi street; decimalLatitude: 47.483817; decimalLongitude: 19.062278; **Identification:** identifiedBy: Attila Rigó; **Event:** eventDate: 23/05/2022; habitat: crevices of pavement
- **Type status:** Other material. **Occurrence:** recordedBy: Attila Rigó; individualCount: 10; reproductiveCondition: fruit-bearing; occurrenceID: 0D619337-97B5-595E-81D5-D06A03453218; **Taxon:** scientificName: Saginaapetala; family: Caryophyllaceae; taxonRank: species; **Location:** continent: Europe; country: Hungary; county: Budapest; municipality: Budapest VIII.; locality: Salgótarjáni street; decimalLatitude: 47.491166; decimalLongitude: 19.095814; **Identification:** identifiedBy: Attila Rigó; **Event:** eventDate: 30/05/2022; habitat: crevices of pavement
- **Type status:** Other material. **Occurrence:** recordedBy: Attila Rigó; individualCount: 60; reproductiveCondition: fruit-bearing; occurrenceID: 40451456-488D-5D0E-A90C-B5A1617293B0; **Taxon:** scientificName: Saginaapetala; family: Caryophyllaceae; taxonRank: species; **Location:** continent: Europe; country: Hungary; county: Budapest; municipality: Budapest VII.; locality: Herzl Tivadar park; decimalLatitude: 47.495876; decimalLongitude: 19.059922; **Identification:** identifiedBy: Attila Rigó; **Event:** eventDate: 02/06/2022; habitat: crevices of pavement
- **Type status:** Other material. **Occurrence:** recordedBy: Attila Rigó; individualCount: 10; reproductiveCondition: fruit-bearing; occurrenceID: 77B90D52-B75C-52D3-A034-A84FAAE1A3DB; **Taxon:** scientificName: Saginaapetala; family: Caryophyllaceae; taxonRank: species; **Location:** continent: Europe; country: Hungary; county: Budapest; municipality: Budapest XIV.; locality: Hősök square; decimalLatitude: 47.514753; decimalLongitude: 19.078455; **Identification:** identifiedBy: Attila Rigó; **Event:** eventDate: 02/06/2022; habitat: crevices of pavement
- **Type status:** Other material. **Occurrence:** recordedBy: Attila Rigó; individualCount: 30; reproductiveCondition: fruit-bearing; occurrenceID: F2E02297-6833-5F94-97B1-38FC0346B8E1; **Taxon:** scientificName: Saginaapetala; family: Caryophyllaceae; taxonRank: species; **Location:** continent: Europe; country: Hungary; county: Budapest; municipality: Budapest XI.; locality: Savoya park; decimalLatitude: 47.436168; decimalLongitude: 19.040259; **Identification:** identifiedBy: Attila Rigó; **Event:** eventDate: 03/06/2022; habitat: crevices of pavement
- **Type status:** Other material. **Occurrence:** recordedBy: Attila Rigó; individualCount: 3; reproductiveCondition: fruit-bearing; occurrenceID: AAB4C2EA-D80B-5C01-947C-F8BF4A5B3136; **Taxon:** scientificName: Saginaapetala; family: Caryophyllaceae; taxonRank: species; **Location:** continent: Europe; country: Hungary; county: Budapest; municipality: Budapest XI.; locality: Fehérvári road; decimalLatitude: 47.440370; decimalLongitude: 19.036607; **Identification:** identifiedBy: Attila Rigó; **Event:** eventDate: 03/06/2022; habitat: crevices of pavement
- **Type status:** Other material. **Occurrence:** recordedBy: Attila Rigó; individualCount: 5; reproductiveCondition: fruit-bearing; occurrenceID: E2C4753C-C79B-555D-90B5-3BD39FEF6EFF; **Taxon:** scientificName: Saginaapetala; family: Caryophyllaceae; taxonRank: species; **Location:** continent: Europe; country: Hungary; county: Budapest; municipality: Budapest XI.; locality: Gellért square; decimalLatitude: 47.483955; decimalLongitude: 19.053079; **Identification:** identifiedBy: Attila Rigó; **Event:** eventDate: 03/06/2022; habitat: crevices of pavement
- **Type status:** Other material. **Occurrence:** recordedBy: Attila Rigó; individualCount: 30; reproductiveCondition: fruit-bearing; occurrenceID: A72476D3-F445-59F3-958B-C98B1CCF1395; **Taxon:** scientificName: Saginaapetala; family: Caryophyllaceae; taxonRank: species; **Location:** continent: Europe; country: Hungary; county: Budapest; municipality: Budapest VI.; locality: Westend rooftop; decimalLatitude: 47.512864; decimalLongitude: 19.058991; **Identification:** identifiedBy: Attila Rigó; **Event:** eventDate: 04/06/2022; habitat: crevices of pavement
- **Type status:** Other material. **Occurrence:** recordedBy: Attila Rigó; individualCount: 30; reproductiveCondition: fruit-bearing; occurrenceID: CAF3709A-A2FE-5C17-969B-709898CFF9D4; **Taxon:** scientificName: Saginaapetala; family: Caryophyllaceae; taxonRank: species; **Location:** continent: Europe; country: Hungary; county: Budapest; municipality: Budapest VI.; locality: Eiffel square; decimalLatitude: 47.509606; decimalLongitude: 19.057631; **Identification:** identifiedBy: Attila Rigó; **Event:** eventDate: 04/06/2022; habitat: crevices of pavement
- **Type status:** Other material. **Occurrence:** recordedBy: Attila Rigó; individualCount: 10; reproductiveCondition: fruit-bearing; occurrenceID: AA279DE1-87DE-5382-845D-5F6B2E485431; **Taxon:** scientificName: Saginaapetala; family: Caryophyllaceae; taxonRank: species; **Location:** continent: Europe; country: Hungary; county: Budapest; municipality: Budapest II.; locality: Temető street; decimalLatitude: 47.566211; decimalLongitude: 18.956976; **Identification:** identifiedBy: Attila Rigó; **Event:** eventDate: 12/06/2022; habitat: crevices of pavement
- **Type status:** Other material. **Occurrence:** recordedBy: Attila Rigó; individualCount: 30; reproductiveCondition: fruit-bearing; occurrenceID: AE2B7154-01C9-53F9-B81C-E6EED8F33859; **Taxon:** scientificName: Saginaapetala; family: Caryophyllaceae; taxonRank: species; **Location:** continent: Europe; country: Hungary; county: Budapest; municipality: Budapest XI.; locality: Placid atya park; decimalLatitude: 47.478078; decimalLongitude: 19.041953; **Identification:** identifiedBy: Attila Rigó; **Event:** eventDate: 22/06/2022; habitat: crevices of pavement

#### Notes

A native weed in Hungary which is currently expanding its range mainly in urban areas (Schmidt 2019). Recently found in many Hungarian towns and cities and had one record from Budapest (Dávid Schmidt ined.). We found that this species is widespread in the urban areas of Budapest.

### Sagina micropetala

Rauschert 1969

65B499CC-F456-516F-919C-FBC227CAF720

#### Materials

- **Type status:** Other material. **Occurrence:** recordedBy: Attila Rigó; individualCount: 20; reproductiveCondition: fruit-bearing; occurrenceID: 861CB4B5-F06E-545D-AE62-CBEE31045434; **Taxon:** scientificName: Saginamicropetala; family: Caryophyllaceae; taxonRank: species; **Location:** continent: Europe; country: Hungary; county: Budapest; municipality: Budapest III.; locality: Gyűrű street; decimalLatitude: 47.598048; decimalLongitude: 19.060886; **Identification:** identifiedBy: Attila Rigó; **Event:** eventDate: 31/05/2023; habitat: crevices of pavement

#### Notes

A native weed in Hungary (Király 2009) with very few recent records (Mesterházy and Kulcsár 2015). This is the first published occurrence of this species in an urban environment, although it had been found in Budapest before (Tamás Rédei ex litt.).

### Saponaria ocymoides

L. 1753

9142520F-8E3C-5221-A8FF-AD22005837F5

#### Materials

- **Type status:** Other material. **Occurrence:** recordedBy: Attila Rigó; individualCount: 10; reproductiveCondition: in bloom; occurrenceID: E3C04068-6A6A-5F51-A71D-A4E04161E1E1; **Taxon:** scientificName: Saponariaocymoides; family: Caryophyllaceae; taxonRank: species; **Location:** continent: Europe; country: Hungary; county: Budapest; municipality: Budapest III.; locality: Ármány street; decimalLatitude: 47.593588; decimalLongitude: 19.060615; **Identification:** identifiedBy: Attila Rigó, Ákos Malatinszky; **Event:** eventDate: 24/04/2020; habitat: crevices of pavement

#### Notes

A European-Mediterranean species that is used as an ornamental in other parts of Europe. Occurs as a rare casual alien in the Czechia and Belgium (Verloove 2006 and Pyšek et al. 2022). In Hungary, it has one recent record (Wirth et al. 2020a).

### Sisymbrium irio

L. 1753

007A3A52-8E57-5DD7-A34C-79E09E4D7B4D

#### Materials

- **Type status:** Other material. **Occurrence:** recordedBy: Attila Rigó, Ákos Malatinszky; individualCount: 70; reproductiveCondition: fruit-bearing; occurrenceID: B500D70F-CB30-5131-9D46-4BB34CD3BE68; **Taxon:** scientificName: Sisymbriumirio; family: Brassicaceae; taxonRank: species; **Location:** continent: Europe; country: Hungary; county: Budapest; municipality: Budapest XIII.; locality: Lehel street; decimalLatitude: 47.517988; decimalLongitude: 19.063308; **Identification:** identifiedBy: Attila Rigó; **Event:** eventDate: 16/05/2022; habitat: road crevices, urban hedges, lawns

#### Notes

A Mediterranean weed that is naturalised worldwide (Kim et al. 2021). It had some previous records from Hungary, but was labelled extinct (Soó 1968). We suggest that this species is locally naturalised in Budapest.

### Talinum paniculatum

(Jacq.) Gaertn. 1791

7A6E4F50-0414-5536-A53C-7232C9DE31CE

#### Materials

- **Type status:** Other material. **Occurrence:** recordedBy: Attila Rigó; individualCount: 4; reproductiveCondition: fruit-bearing; occurrenceID: B856A021-BC61-500A-A8DD-964A875FBEAF; **Taxon:** scientificName: Talinumpaniculatum; family: Talinaceae; taxonRank: species; **Location:** continent: Europe; country: Hungary; county: Budapest; municipality: Budapest XI.; locality: Csíkihegyek street; decimalLatitude: 47.472860; decimalLongitude: 18.996353; **Identification:** identifiedBy: Attila Rigó; **Event:** eventDate: 28/09/2022; habitat: crevices of road pavement

#### Notes

Native to tropical America, used as an ornamental and became naturalised and invasive in large parts of the tropical and subtropical regions (Walthers et al. 2011). It appears as a rare casual alien in temperate regions, for example, in Italy (Spampinato et al. 2022) and Ukraine (Shynder et al. 2022). New to the adventive flora of Hungary.

### Vaccaria hispanica

(Mill.) Rauschert 1965

F9770E0B-4896-5780-8359-F2DF6BB5AA7B

#### Materials

- **Type status:** Other material. **Occurrence:** recordedBy: Attila Rigó; individualCount: 1; reproductiveCondition: in bloom; occurrenceID: 427B4DAC-48E5-52C2-A236-C506A2FAB36A; **Taxon:** scientificName: Vaccariahispanica; family: Caryophyllaceae; taxonRank: species; **Location:** continent: Europe; country: Hungary; county: Budapest; municipality: Budapest XIII.; locality: Szabolcs street; decimalLatitude: 47.516879; decimalLongitude: 19.067071; **Identification:** identifiedBy: Attila Rigó; **Event:** eventDate: 16/05/2022; habitat: crack at the base of a building

#### Notes

A rare archaeophyte in Hungary with a decreasing number of localities (Király 2009 and Csiky et al. 2023). Its presence in an urban environment is noteworthy.

### Verbena bonariensis

L. 1753

EB31D1CD-F3BB-5026-ACBF-712432E47F30

#### Materials

- **Type status:** Other material. **Occurrence:** recordedBy: Attila Rigó; individualCount: 2; reproductiveCondition: non-reproductive; occurrenceID: 3E56C475-A72A-5AEF-AD5C-D786EB03D04A; **Taxon:** scientificName: Verbenabonariensis; family: Verbenaceae; taxonRank: species; **Location:** continent: Europe; country: Hungary; county: Budapest; municipality: Budapest I.; locality: Márvány street; decimalLatitude: 47.495629; decimalLongitude: 19.027060; **Identification:** identifiedBy: Attila Rigó; **Event:** eventDate: 13/05/2022; habitat: crevices of pavement
- **Type status:** Other material. **Occurrence:** recordedBy: Attila Rigó; individualCount: 2; reproductiveCondition: non-reproductive; occurrenceID: 948B72B4-C54C-5AA8-90B0-D57B98A2D146; **Taxon:** scientificName: Verbenabonariensis; family: Verbenaceae; taxonRank: species; **Location:** continent: Europe; country: Hungary; county: Budapest; municipality: Budapest VIII.; locality: Vásár street; decimalLatitude: 47.493412; decimalLongitude: 19.073037; **Identification:** identifiedBy: Attila Rigó; **Event:** eventDate: 17/06/2022; habitat: crevices of pavement
- **Type status:** Other material. **Occurrence:** recordedBy: Attila Rigó; individualCount: 3; reproductiveCondition: non-reproductive; occurrenceID: D790E8A0-AEA7-5488-B3AF-CEC0879CA608; **Taxon:** scientificName: Verbenabonariensis; family: Verbenaceae; taxonRank: species; **Location:** continent: Europe; country: Hungary; county: Budapest; municipality: Budapest VIII.; locality: Corvin alley; decimalLatitude: 47.485875; decimalLongitude: 19.071200; **Identification:** identifiedBy: Attila Rigó; **Event:** eventDate: 19/06/2022; habitat: crevices of pavement
- **Type status:** Other material. **Occurrence:** recordedBy: Attila Rigó; individualCount: 5; reproductiveCondition: non-reproductive; occurrenceID: 431AD51B-0633-5A85-8074-F818B07CCB4F; **Taxon:** scientificName: Verbenabonariensis; family: Verbenaceae; taxonRank: species; **Location:** continent: Europe; country: Hungary; county: Budapest; municipality: Budapest V.; locality: Jászai Mari square; decimalLatitude: 47.513003; decimalLongitude: 19.047315; **Identification:** identifiedBy: Attila Rigó; **Event:** eventDate: 24/06/2022; habitat: crevices of pavement
- **Type status:** Other material. **Occurrence:** recordedBy: Attila Rigó; individualCount: 7; reproductiveCondition: in bloom; occurrenceID: 01A7B72D-A253-532C-8554-918C832A6A24; **Taxon:** scientificName: Verbenabonariensis; family: Verbenaceae; taxonRank: species; **Location:** continent: Europe; country: Hungary; county: Budapest; municipality: Budapest XIII.; locality: Jászai Mari square; decimalLatitude: 47.513694; decimalLongitude: 19.047731; **Identification:** identifiedBy: Attila Rigó; **Event:** eventDate: 24/06/2022; habitat: crevices of pavement
- **Type status:** Other material. **Occurrence:** recordedBy: Attila Rigó; individualCount: 4; reproductiveCondition: in bloom; occurrenceID: E9A99FF0-E41E-5429-9C59-B73ADDF9605C; **Taxon:** scientificName: Verbenabonariensis; family: Verbenaceae; taxonRank: species; **Location:** continent: Europe; country: Hungary; county: Budapest; municipality: Budapest VIII.; locality: Mikszáth Kálmán square; decimalLatitude: 47.489953; decimalLongitude: 19.066680; **Identification:** identifiedBy: Attila Rigó; **Event:** eventDate: 10/08/2022; habitat: crevices of pavement
- **Type status:** Other material. **Occurrence:** recordedBy: Attila Rigó; individualCount: 10; reproductiveCondition: in bloom; occurrenceID: 493DC7FB-1E1D-54F5-A115-FB5286B017EB; **Taxon:** scientificName: Verbenabonariensis; family: Verbenaceae; taxonRank: species; **Location:** continent: Europe; country: Hungary; county: Budapest; municipality: Budapest III.; locality: Árpád street; decimalLatitude: 47.590743; decimalLongitude: 19.051335; **Identification:** identifiedBy: Attila Rigó; **Event:** eventDate: 28/09/2022; habitat: disturbed ground in a garden store
- **Type status:** Other material. **Occurrence:** recordedBy: Attila Rigó; individualCount: 3; reproductiveCondition: fruit-bearing; occurrenceID: 7A3945FC-FDBF-5FCD-8AA1-4C64E272595F; **Taxon:** scientificName: Verbenabonariensis; family: Verbenaceae; taxonRank: species; **Location:** continent: Europe; country: Hungary; county: Budapest; municipality: Budapest II.; locality: Szabó Magda promenade; decimalLatitude: 47.510143; decimalLongitude: 19.026267; **Identification:** identifiedBy: Attila Rigó; **Event:** eventDate: 05/10/2022; habitat: crevices of pavement

#### Notes

Native to South America and used as an ornamental worldwide. Appears as a casual and as a naturalised alien in many countries worldwide (Galasso et al. 2019). This species has few recent casual occurrences in Hungary (Wirth et al. 2020a). New to Budapest.

## Analysis

We found 973 spontaneously or subspontaneously occurring plant taxa in the artificial secondary habitats in the built-up parts of Budapest between 2018 and 2023 (Table 1, Suppl. materials 2, 3). A total of 47% of them were native, 19% of them were archaeophyte and 28% of them were neophyte, while the status of 6% of them is questionable.

Considering the frequency of the species, it can be concluded that there were very few really common species in the urban areas of Budapest. There are only 12 species that were found in more than 40% of the surveyed street sections and squares, while 27 species were found in 20–40%, 35 species were found in 10–20%, 188 species were found in 1–10% and 711 species were found in less than 1% of surveyed units. The 20 most frequent species of Budapest were *Polygonumaviculare* L. (found in 79% of the surveyed units), *Taraxacumofficinale* F.H.Wigg (75%)., *Erigeroncanadensis* L. (59%), *Setariaviridis* (L.) P.Beauv. (58%), *Sonchusoleraceus* L. (56%), *Chenopodiumalbum* L. (52%), *Stellariamedia* (L.) Vill. (51%), *Erigeronannuus* (L.) Pers. (43%), *Ailanthusaltissima* (Mill.) Swingle (42%), *Plantagomajor* L. (41%), *Eleusineindica* (L.) Gaertn. (41%), *Loliumperenne* L. (41%), *Eragrostisminor* Host (40%), *Portulacaoleracea* L. (39%), *Convolvulusarvensis* L. (38%), *Poaannua* L. (38%), *Plantagolanceolata* L. (37%), *Oxalisdillenii* Jacq. (36%), *Ballotanigra* L. (34%) and *Oxaliscorniculata* L. (32%). Five of these taxa are native, one is a doubtfully native taxon (native or archaeophyte), eight are archaeophytes and six are neophytes. There were 38 taxa that occurred in all 23 districts of Budapest. A total of 184 taxa occurred in 12 or more districts, while 789 occurred in 11 or less districts, 445 of which only occurred in one or two districts.

## Discussion

The main result of our research is the compiled inventory of the urban flora of Budapest with the distribution of each taxon. It is neccesary to make an inventory of the flora of different, well-defined areas from time to time so that changes in the flora can be monitored. The 973 found taxa is 28.3 % of the Hungarian flora (3437 taxa) according to Bartha et al. (2023), while Budapest is only 0.5% of the country's area. As different urban floristic studies use completely different methodologies, comparing the results does not always lead to good conclusions, but some general conclusions can be drawn. The 973 plant species found in Budapest can be considered below average from an international perspective (Pysek 1998), but most of the urban floristic researches include natural and semi-natural habitats within cities, which we excluded from our research.

In Hungary, the floristic research of Pécs (southern Hungary) took place most recently (Wirth et al. 2020c), resulting in 1641 taxa found in and around Pécs and the native-archaeophyte-neophyte ratio was 7-1-2. In Budapest, on the other hand, we found 973 species and the native-archaeophyte-neophyte ratio was 5-2-3. One possible reason for this is that Wirth et al. (2020c) also examined the plants of natural and semi-natural habitats in Pécs, which we omitted in Budapest.

By reviewing the changes in the urban flora of Budapest from the beginning of the 19^th^ century, we can see that the records of taxa from the urban habitats of Budapest is constantly increasing. Sadler (1840) reported 366 taxa, Borbás (1879) reported 441 taxa and Hegedűs (1994) reported 568 taxa from the urban habitats of Budapest.

All seven species that are new to the Hungarian flora are casual aliens which escaped from cultivation. Only *Clinopodiumnepeta* (L.) Kuntze and *Linariamaroccana* Hook.f. has more than one record. Rediscovered species are also casuals, except *Sisymbriumirio* L., which we consider locally naturalised in Budapest. We detected a major expansion in the case of six species (*Daturawrightii* Regel, *Erigeronsumatrensis* Retz., *Euphorbiaprostrata* Aiton, *Nepetaracemosa* Bergmans ex Stearn, *Perovskiaatriplicifolia* Benth., *Saginaapetala* Ard.). Of these, *Saginaapetala* is native and the expansion of the species in urban habitats is well documented (Schmidt 2019). We suggest that *Daturawrightii* Regel, *Erigeronsumatrensis* Retz., *Euphorbiaprostrata* Aiton, *Nepetaracemosa* Bergmans ex Stearn, *Perovskiaatriplicifolia* Benth. are locally naturalised in Budapest.

In several cases, protected and rare casual alien ferns were found in urban environments (e.g. Tamás et al. 2017). Of the 18 ferns found in Budapest, seven are legally protected in Hungary (*Aspleniumadiantum*-*nigrum* L., *Aspleniumscolopendrium* L., *Dryopteriscarthusiana* (Vill.) H.P.Fuchs, *Gymnocarpiumrobertianum* (Hoffm.) Newman, *Polystichumaculeatum* (L.) Roth, *Polystichumsetiferum* Rosendahl and *Thelypterispalustris* Schott) and four are casual aliens (*Adiantumcapillus-veneris* L., *Cyrtomiumfalcatum* (L.f.) C.Presl, *Cyrtomiumfortunei* J.Sm. and *Pterismultifida* Poir.).

Our research draws attention to the fact that the flora of urban areas is under-researched. The appearance of certain species in urban environments is accidental, therefore small-scale, high-resolution mapping is recommended in order to better understand urban flora.

## Supplementary Material

XML Treatment for Achillea filipendulina

XML Treatment for Allium ramosum

XML Treatment for Artemisia scoparia

XML Treatment for Begonia cucullata

XML Treatment for Borago officinalis

XML Treatment for Bromus catharticus

XML Treatment for Campanula portenschlagiana

XML Treatment for Catapodium rigidum

XML Treatment for Cenchrus alopecuroides

XML Treatment for Chasmanthium latifolium

XML Treatment for Clinopodium nepeta

XML Treatment for Cyperus eragrostis

XML Treatment for Cyrtomium falcatum

XML Treatment for Cyrtomium fortunei

XML Treatment for Datura innoxia

XML Treatment for Datura wrightii

XML Treatment for Digitaria ciliaris

XML Treatment for Eclipta prostrata

XML Treatment for Eragrostis spectabilis

XML Treatment for Eragrostis virescens

XML Treatment for Erigeron sumatrensis

XML Treatment for Euphorbia prostrata

XML Treatment for Euphorbia serpens

XML Treatment for Ficus carica

XML Treatment for Glebionis coronaria

XML Treatment for Heliopsis helianthoides

XML Treatment for Lagenaria siceraria

XML Treatment for Lepidium didymum

XML Treatment for Linaria maroccana

XML Treatment for Matthiola longipetala subsp. bicornis

XML Treatment for Nassella tenuissima

XML Treatment for Nepeta racemosa

XML Treatment for Nicotiana alata

XML Treatment for Nicotiana sylvestris

XML Treatment for Panicum riparium

XML Treatment for Panicum virgatum

XML Treatment for Perovskia atriplicifolia

XML Treatment for Persicaria orientalis

XML Treatment for Polygonum rurivagum

XML Treatment for Polypogon viridis

XML Treatment for Polystichum setiferum

XML Treatment for Pteridium aquilinum

XML Treatment for Sagina apetala

XML Treatment for Sagina micropetala

XML Treatment for Saponaria ocymoides

XML Treatment for Sisymbrium irio

XML Treatment for Talinum paniculatum

XML Treatment for Vaccaria hispanica

XML Treatment for Verbena bonariensis

56C69B59-01CE-5BA0-9B23-2423A4EE199D 10.3897/BDJ.11.e110450.suppl1 Supplementary material 1 New and noteworthy floristic records from Budapest (materials) Data type occurrences, individual count, phenology Brief description Materials for the new and noteworthy floristic records found in the urban flora of Budapest between 2018 and 2023. Includes: scientificName = scientific name of the taxon; family; continent; country; county; municipality = districts of Budapest; locality = name of the public place; decimalLatitude; decimalLongitude; eventDate; habitat; individualCount; reproductiveCondition; occurrenceID; recordedBy; identifiedBy File: oo_881557.xlsx https://binary.pensoft.net/file/881557 Attila Rigó, Ákos Malatinszky, Zoltán Barina

467E9554-7061-52B1-AAF7-54F788B801C9 10.3897/BDJ.11.e110450.suppl2 Supplementary material 2 Inventory of the secondary habitats of the urban, built-up areas of Budapest Data type residency status, distribution Brief description An inventory of the taxa found by the authors in the secondary habitats of the urban, built-up areas of Budapest between 2018 and 2023. Includes: Taxon = scientific name of the taxon; Residency status (abbreviations: ntv = native, arc = archaeophyte, neo = neophyte); Distribution = list of the districts of Budapest where the taxon occurs. Data source: Budapest districts taxon list. File: oo_881558.xlsx https://binary.pensoft.net/file/881558 Attila Rigó, Ákos Malatinszky, Zoltán Barina

BB36CC39-A5B2-5F8A-B346-760F7EC25A2F 10.3897/BDJ.11.e110450.suppl3 Supplementary material 3 Budapest districts taxon list Data type occurrences Brief description Taxon list of each 23 districts of Budapest. 1 = the taxon is registered in the district; 0 = the taxon is not registered in the district. File: oo_881559.xlsx https://binary.pensoft.net/file/881559 Attila Rigó, Ákos Malatinszky, Zoltán Barina

## Figures and Tables

**Table 1.** Inventory of the flora of secondary habitats in the urban, built-up areas of Budapest (Hungary). Summary: Taxon = scientific name of the taxon; Residency status = residency status of the taxon according to Csiky et al. (2023) (abbreviations: ntv = native, arc = archaeophyte, neo = neophyte); Distribution = list of the districts of Budapest where the taxon occurs, districts are indicated by Roman numerals (from I. to XXIII.).

Taxon	Residency status	Distribution
*Abiesconcolor* (Gordon) Lindl. ex. Hildebr.	neo	II.
*Abutilontheophrasti* Medik.	arc	III. VI.
*Acercampestre* L.	ntv	I. II. III. IV. V. VI. VIII. IX. X. XI. XII. XIII. XIV. XV. XVI. XIX. XX. XXI. XXII. XIII.
*Acernegundo* L.	neo	I. II. III. IV. VI. VII. VIII. IX. X. XI. XII. XIII. XIV. XV. XVI. XVII. XIX. XX. XXI. XXIII.
*Acerplatanoides* L.	ntv	I. II. III. IV. VIII. IX. X. XI. XII. XIII. XIV. XV. XVI. XVII. XIX. XX. XXI. XXII.
*Acerpseudoplatanus* L.	ntv	I. II. III. IV. VIII. IX. X. XI. XII. XIII. XIV. XV. XVI. XIX. XX. XXII.
*Acersaccharinum* L.	neo	III. IV. VIII. IX. XI. XIII. XIV. XVII. XX. XXI.
*Acertataricum* L.	ntv	XI. XXII.
*Achilleacollina* (Becker ex Rchb.f.) Heimerl	ntv	I. II. III. IV. V. VI. VII. VIII. IX. X. XI. XII. XIII. XIV. XV. XVI. XVII. XVIII. XIX. XX. XXI. XXII. XXIII.
*Achilleafilipendulina* Lam.	neo	I. II. VIII. IX. XIII. XIV.
*Achilleanobilis* L.	ntv	XV.
*Achilleapannonica* Scheele	ntv	II. XI. XIV. XV.
*Achnatherumvirescens* (Trin.) Banfi, Galasso & Bartolucci	ntv	III. XII.
*Adiantumcapillus-veneris* L.	neo	IX. XII.
*Adonisaestivalis* L.	arc	XI.
*Adonisvernalis* L.	ntv	XI.
*Aegilopscylindrica* Host	ntv	III. XI. XIII. XX.
*Aesculushippocastanum* L.	neo	I. III. XI. XII. XVI. XVII. XIX.
*Aethionemasaxatile* (L.) W.T.Aiton	ntv	III.
*Aethusacynapium* L.	ntv/arc	XII.
*Ageratumhoustonianum* Mill.	neo	XXII.
*Agrimoniaeupatoria* L.	ntv	I. II. III. X. XI. XIII.
*Agropyroncristatum* (L.) Gaertn.	ntv	IX.
*Agrostiscapillaris* L.	ntv	III. XVI.
*Agrostisstolonifera* L.	ntv	III. VI. IX. XIV.
*Ailanthusaltissima* (Mill.) Swingle	neo	I. II. III. IV. V. VI. VII. VIII. IX. X. XI. XII. XIII. XIV. XV. XVI. XVII. XVIII. XIX. XX. XXI. XXII. XXIII.
*Ajugachamaepitys* (L.) Schreb.	arc	II. XII. XIII. XIV. XXIII.
*Ajugagenevensis* L.	ntv	II.
*Ajugareptans* L.	ntv	III. XIII. XV. XVI.
*Albiziajulibrissin* Durazz.	neo	XI. XVI. XXII.
*Alcearosea* L.	neo	III. IV. IX. XI. XII. XV. XVII. XXII.
*Alismaplantago-aquatica* L.	ntv	XVII.
*Alkekengiofficinarum* Moench	arc	VIII.
*Alliariapetiolata* (M.Bieb.) Cavara & Grande	ntv	I. II. III. V. VIII. XI. XII. XIII. XIV. XV.
*Alliumramosum* L.	neo	IX. XI. XXI.
*Alliumschoenoprasum* L.	neo	XII.
*Alliumscorodoprasum* L.	arc	II. XII. XX.
*Alliumvineale* L.	ntv	II. IX.
*Alopecurusmyosuroides* Huds.	arc	VI. XIII.
*Althaeacannabina* L.	arc/neo	XI.
*Althaeaofficinalis* L.	arc	III.
*Alyssumalyssoides* L.	ntv	XIII.
*Alyssummontanum* L.	ntv	XI.
*Amaranthusalbus* L.	neo	II. IV. V. VIII. IX. X. XI. XII. XIII. XIV. XV. XX. XXI.
*Amaranthusblitoides* S.Watson	arc	II. IV. V. VII. VIII. IX. X. XII. XIII.
*Amaranthusblitum* L.	neo	I. II. III. IV. V. VI. VII. VIII. IX. XI. XIII. XIV. XV. XX. XXI.
Amaranthusblitumsubsp.emarginatus (Salzm. ex Uline & W.L.Bray) Carretero, Muñoz Garm. & Pedrol	neo	IX.
*Amaranthuscruentus* L.	neo	I.
*Amaranthusdeflexus* L.	neo	I. II. III. IV. V. VI. VII. VIII. IX. X. XI. XII. XIII. XIV. XV. XVI. XVII. XVIII. XIX. XX. XXI. XXII. XXIII.
*Amaranthushybridus* L.	neo	IX.
*Amaranthushypochondriacus* L.	neo	IX. XI.
*Amaranthuspowellii* S.Watson	neo	I. II. III. IV. VI. VII. VIII. IX. X. XI. XII. XIII. XIV. XV. XVI. XVII. XVIII. XIX. XX. XXI. XXII. XXIII.
*Amaranthusretroflexus* L.	neo	I. II. III. IV. V. VI. VII. VIII. IX. X. XI. XII. XIII. XIV. XV. XVI. XVIII. XIX. XX. XXI. XXII. XXIII.
*Ambrosiaartemisiifolia* L.	neo	I. II. III. IV. V. VI. VII. VIII. IX. X. XI. XII. XIII. XIV. XV. XVI. XVII. XVIII. XIX. XX. XXI. XXII. XXIII.
*Amorphafruticosa* L.	neo	II. III. IV. V. XI. XIII. XIX. XXI.
*Anchusaofficinalis* L.	ntv	I. II. III. IV. VI. VIII. IX. X. XI. XII. XIII. XIV. XV. XVI. XVII. XX. XXI. XXIII.
*Anemonesylvestris* L.	ntv	XIII.
*Anethumgraveolens* L.	arc	VIII.
*Anthemisarvensis* L.	arc	III.
*Anthemisruthenica* M.Bieb.	ntv/arc	VI. IX. XI. XIII. XX.
*Anthriscuscaucalis* M.Bieb.	arc	VI. VIII. IX. X. XI. XIII. XV.
*Anthriscuscerefolium* Hoffm.	arc	I. II. III. IV. V. VI. VII. VIII. IX. X. XI. XII. XIII. XIV. XV. XVI. XX. XXI.
*Anthriscussylvestris* (L.) Hoffm.	ntv	III. IX. XII.
*Anthyllisvulneraria* L.	ntv	IX.
*Antirrhinummajus* L.	neo	II. III. VIII. IX. XI. XIV. XV. XVI.
Apera spica-venti (L.) P.Beauv.	arc	II. V. VI. VIII. XI. XIII. XIV. XV. XVII. XX. XXIII.
*Aphanesarvensis* L.	arc	III. XIV.
*Apiumgraveolens* L.	arc	IX.
*Apiumrepens* (Jacq.) Lag.	ntv	XIII.
*Aquilegiavulgaris* L.	ntv	IV. XI. XII. XV. XXI. XXII.
*Arabidopsisarenosa* (L.) Lawalrée	ntv	I.
*Arabidopsisthaliana* (L.) Heynh.	ntv	I. II. III. VII. IX. XII. XIII. XIV. XVI.
*Arctiumlappa* L.	arc	I. III. XI. XII. XIII. XIV. XVII.
*Arctiumminus* Schkuhr	arc	III. IX. XII.
*Arctiumtomentosum* Mill.	ntv	I. II. III. IV. VI. VIII. XI. XII. XIV. XX. XXII.
*Arenariaserpyllifolia* L.	ntv	I. II. III. IV. V. VI. VII. VIII. IX. X. XI. XII. XIII. XIV. XV. XVI. XX. XXI. XXII. XXIII.
*Aristolochiaclematitis* L.	arc	III. XI. XXI.
*Armoraciarusticana* G.Gaertn., B.Mey. & Scherb.	arc	XIII.
*Arrhenatherumelatius* (L.) P.Beauv. ex J.Presl & C.Presl	ntv	I. II. III. VIII. IX. X. XI. XII. XIII. XIV. XV. XVII. XIX. XXI. XXIII.
*Artemisiaabsinthium* L.	arc	VIII. X. XI. XIV. XV. XXI.
*Artemisiaalba* Turra	ntv	XI.
*Artemisiaannua* L.	neo	II. III. IV. VII. VIII. IX. XIII.
*Artemisiacampestris* L.	ntv	IX. XXI.
*Artemisiadracunculus* L.	arc	XV.
*Artemisiapontica* L.	ntv	VIII. IX. X. XI. XIII. XV.
*Artemisiascoparia* Waldst. & Kit.	ntv	IX.
*Artemisiaverlotiorum* Lamotte	neo	XI.
*Artemisiavulgaris* L.	ntv	I. II. III. IV. V. VI. VIII. IX. X. XI. XII. XIII. XIV. XV. XVI. XVII. XX. XXI. XXII. XXIII.
*Arumorientale* M.Bieb.	ntv	XII.
*Arundodonax* L.	arc	XV.
*Asarinaprocumbens* Mill.	neo	I.
*Asclepiassyriaca* L.	neo	II. III. IV. VI. VIII. IX. X. XI. XII. XIV. XV. XVI. XVII. XIX. XXI. XXIII.
*Asparagusofficinalis* L.	ntv	IX.
*Asparagusverticillatus* L.	neo	III.
*Asperugoprocumbens* L.	arc	III. XI. XIII. XV.
Aspleniumadiantum-nigrum L.	ntv	VI. VIII. IX. XI. XII. XXI.
Asplenium ruta-muraria L.	ntv	I. II. III. V. VIII. IX. XI. XII.
*Aspleniumscolopendrium* L.	ntv	VIII. IX. XII. XXI.
*Aspleniumtrichomanes* L.	ntv	I. VI. VIII. XII.
*Astragalusasper* Jacq.	ntv	XI.
*Astragaluscicer* L.	ntv	III. XI. XIII. XIV. XVI. XXI.
*Astragalusglycyphyllos* L.	ntv	II. XII. XIII.
*Astragalusonobrychis* L.	ntv	IV. XI. XXI.
Athyrium filix-femina (L.) Roth	ntv	XI.
*Atriplexlittoralis* L.	ntv	III.
*Atriplexoblongifolia* Waldst. & Kit.	ntv	I. II.
*Atriplexpatula* L.	arc	I. II. III. IV. VIII. IX. X. XI. XII. XIII. XV. XVI. XIX. XX. XXI. XXII. XXIII.
*Atriplexprostrata* Boucher ex DC.	ntv	II. III. VIII. XI. XII. XIII. XVII. XXIII.
*Atriplexsagittata* Borkh.	arc	IX.
*Atriplextatarica* L.	arc	I. II. III. IV. VIII. IX. X. XI. XII. XIII. XIV. XV. XX. XXI. XXII. XXIII.
*Avenafatua* L.	arc	XIV.
*Avenasativa* L.	arc	VI. XI.
*Azollafiliculoides* Lam.	neo	IX.
*Ballotanigra* L.	arc	I. II. III. IV. V. VI. VII. VIII. IX. X. XI. XII. XIII. XIV. XV. XVI. XVII. XVIII. XIX. XX. XXI. XXII. XXIII.
*Bassiascoparia* (L.) A.J.Scott	arc/neo	V. VI. VIII. IX. X. XI. XIV. XVI. XX. XXI. XXIII.
*Begoniacucullata* Willd.	neo	XI. XIII.
*Bellisperennis* L.	ntv/arc	I. II. III. IV. V. VI. VII. VIII. IX. X. XI. XII. XIII. XIV. XV. XVI. XVIII. XIX. XX. XXI. XXII. XXIII.
*Berberisaquifolium* Pursh	neo	I. II. III. IV. V. VII. VIII. IX. X. XI. XII. XIII. XIV. XV. XVI. XVII. XIX. XX. XXI.
*Berberisjulianae* C.K.Schneid.	neo	I. III. V. VII. XI.
*Berberisthunbergii* DC.	neo	III.
*Berberisvulgaris* L.	ntv	I. II. V. VIII. XI. XII. XVI. XX.
*Berteroaincana* (L.) DC.	ntv/arc	III. IV. IX. X. XI. XIV. XV. XVII. XXI.
*Berulaerecta* (Huds.) Coville	ntv	XVII.
*Betonicaofficinalis* L.	ntv	XI.
*Betulapendula* Roth	ntv	II. III. IV. IX. X. XI. XIII.
*Bidenscernua* L.	ntv	XXI.
*Bidensfrondosa* L.	neo	XIV. XXI.
*Boragoofficinalis* L.	neo	III.
*Bothriochloaischaemum* (L.) Keng	ntv	III. IV. XI. XII. XIV. XVI. XX. XXI. XXIII.
*Brachypodiumsylvaticum* (Huds.) P.Beauv.	ntv	I. III. XI. XII. XXI.
*Brassicaelongata* Ehrh.	ntv/arc	III.
*Brassicanapus* L.	arc	XXI.
*Brassicarapa* L.	neo	VI. VIII. IX. X. XI. XII. XIV. XXI.
Brassicarapavar.oleiferaDC.	neo	VIII.
*Bromuscatharticus* Vahl	neo	III. XV.
*Bromuscommutatus* Schrad.	ntv	II. III.
*Bromuserectus* Huds.	ntv	XI. XII.
*Bromushordeaceus* L.	ntv/arc	II. V. VI. VIII. IX. X. XI. XII. XIII. XIV. XV. XVI. XX.
*Bromusinermis* Leyss.	ntv	I. III. VIII. IX. X. XI. XII. XIV. XXI. XXIII.
*Bromusjaponicus* Houtt.	arc	III. IV. V. VI. XI. XIV. XX.
*Bromussquarrosus* L.	ntv	VIII. IX. XI. XIII.
*Bromussterilis* L.	arc	I. II. III. IV. V. VI. VII. VIII. IX. X. XI. XII. XIII. XIV. XV. XVI. XVII. XVIII. XIX. XX. XXI. XXII. XXIII.
*Bromustectorum* L.	arc	I. II. III. VI. VIII. IX. X. XI. XII. XIII. XIV. XV. XVI. XVII. XX. XXI.
*Broussonetiapapyrifera* (L.) Vent.	neo	I. II. V. VI. XI. XIII. XIV. XX. XXI.
*Brunneramacrophylla* (Adams) I.M.Johnst.	neo	I. II. III. IV. V. XI. XII. XIII. XIV. XV. XVI. XVII. XIX. XXII.
*Bryoniaalba* L.	ntv/arc	II. III. XI. XII. XV. XXI.
*Buddlejadavidii* Franch.	neo	II. IX. X. XII. XVI. XXI.
*Buglossoidesarvensis* (L.) I.M.Johnst.	ntv	I. III. VI. XI. XIII. XIV. XV. XVI.
*Buniasorientalis* L.	neo	III. XI.
*Bupleurumfalcatum* L.	ntv	XII.
*Butomusumbellatus* L.	ntv	XIV.
*Buxussempervirens* L.	neo	III. XI.
*Calendulaofficinalis* L.	arc	I. III. IX. XI. XIII. XV. XIX.
*Calepinairregularis* Thell.	arc	I. III.
*Calystegiasepium* (L.) R.Br.	ntv	I. II. III. V. VIII. IX. X. XI. XII. XIII. XVII. XXII. XXIII.
*Camelinamicrocarpa* Andrz. ex DC.	arc	XII.
*Campanulabononiensis* L.	ntv	XII.
*Campanulacarpatica* Jacq.	neo	IX.
*Campanulaglomerata* L.	ntv	XII.
*Campanulapersicifolia* L.	ntv	III. XI. XVI.
*Campanulaportenschlagiana* Roem. & Schult.	neo	XII.
*Campanulaposcharskyana* Degen	neo	III. XI. XII. XIX.
*Campanularapunculoides* L.	ntv	I. II. III. XI. XII. XIV. XV. XVI. XVII. XXII.
*Campanulatrachelium* L.	ntv	XII.
*Campsisradicans* (L.) Bureau	neo	I. II. III. VI. VIII. IX. XI. XIV. XV. XVI. XVII. XX. XXI. XXII.
*Cannabissativa* L.	arc	II. V. VII. VIII. IX. XIII. XIV. XX.
*Capsellabursa-pastoris* Medik.	arc	I. II. III. IV. V. VI. VII. VIII. IX. X. XI. XII. XIII. XIV. XV. XVI. XX. XXI. XXII.
*Capsicumannuum* L.	neo	VI. XI.
*Cardaminebulbifera* Crantz	ntv	II. XII.
*Cardaminehirsuta* L.	ntv	I. II. III. V. VIII. IX. XI. XII. XIII. XIV. XV. XVI.
*Cardamineocculta* Hornem.	neo	XIV.
*Carduusacanthoides* L.	arc	I. II. III. IV. V. VII. VIII. IX. X. XI. XII. XIII. XIV. XV. XVI. XVII. XX. XXI. XXIII.
*Carduusnutans* L.	arc	III. VIII. IX. X. XI. XXIII.
*Carexacuta* L.	ntv	XX.
*Carexacutiformis* Ehrh.	ntv	IV. XXIII.
*Carexcaryophyllea* Latourr.	ntv	III.
*Carexdivulsa* Stokes	ntv	XI.
*Carexhirta* L.	ntv	I. II. III. IV. V. VI. VIII. IX. X. XI. XII. XIII. XV. XX. XXI. XXIII.
*Carexleersii* F.W.Schultz	ntv	II.
*Carexpairae* F.W.Schultz	ntv	XII.
*Carexpilosa* Scop.	ntv	XII.
*Carexpraecox* Schreb.	ntv	IV. IX. X. XII. XIV. XVI. XXI.
*Carexspicata* Huds.	ntv	II. XII. XIII. XIV. XV.
*Carexstenophylla* Wahlenb.	ntv	IX.
*Carlinavulgaris* L.	ntv	III. IV. IX.
*Carpinusbetulus* L.	ntv	III. IX. XII.
*Caryopteris×clandonensis* A.Simmonds	neo	III.
*Catalpabignonioides* Walter	neo	IX. XVII.
*Catapodiumrigidum* (L.) C.E.Hubb.	neo	III.
*Catharanthusroseus* (L.) G.Don	neo	XI.
*Celosiaargentea* L.	neo	IX.
*Celtisaustralis* L.	neo	XIX.
*Celtisoccidentalis* L.	neo	I. II. III. IV. V. VI. VII. VIII. IX. X. XI. XII. XIII. XIV. XV. XVI. XVII. XVIII. XIX. XX. XXI. XXII. XXIII.
*Cenchrusalopecuroides* (L.) Thunb.	neo	VIII. IX. XI. XV. XXI.
*Cenchrusincertus* M.A.Curtis	neo	IV. IX. XIV. XXI.
*Centaureaarenaria* M.Bieb.	ntv	XV.
*Centaureacyanus* L.	arc	IX. XIII.
Centaureajaceasubsp.angustifolia (DC.)	ntv	II. III. IX. X. XI. XII. XIV. XV. XVI. XXII.
Centaureascabiosasubsp.sadleriana (Janka) Asch. & Graebn.	ntv	I. III. VIII. XI. XII. XIII. XV. XXI. XXIII.
*Centaureastoebe* L.	ntv/arc	I. II. III. IV. VIII. IX. X. XI. XII. XIV. XV. XX. XXI. XXIII.
*Centaureatriumfettii* All.	ntv	XI.
*Cerastiumbrachypetalum* Pers.	ntv	III.
Cerastiumfontanumsubsp.vulgare (Hartm.) Greuter & Burdet	ntv	I. II. III. IV. V. VI. VIII. IX. X. XI. XII. XIII. XIV. XV. XVI. XXIII.
*Cerastiumglomeratum* Thuill.	ntv	III.
*Cerastiumpumilum* Curtis	ntv	III. XV. XVI.
Cerastiumpumilumvar.glutinosum(Fries) E.Rico	ntv	XIV.
*Cerastiumsemidecandrum* L.	ntv	XI. XIII.
*Cerastiumtomentosum* L.	neo	III. XI. XII. XV.
*Ceratocephalatesticulata* (Crantz) Besser	arc	XI.
*Ceratostigmaplumbaginoides* Bunge	neo	XV. XXII.
*Cercissiliquastrum* L.	neo	I. II. V. VIII. IX. XI. XII. XIII. XIV.
*Cerintheminor* L.	ntv	XII.
*Chaenorhinumminus* (L.) Lange	arc	II. V. VIII. IX. XI. XII. XIII. XIV. XV.
*Chaerophyllumaromaticum* L.	ntv	XII.
*Chaerophyllumtemulum* L.	ntv	XI. XII.
*Chamaecytisusaustriacus* (L.) Link	ntv	XI. XII.
*Chasmanthiumlatifolium* (Michx.) H.O.Yates	neo	VIII.
*Chelidoniummajus* L.	ntv	I. II. III. IV. V. VI. VII. VIII. IX. X. XI. XII. XIII. XIV. XV. XVI. XVII. XVIII. XIX. XX. XXI. XXII. XXIII.
*Chenopodiumalbum* L.	arc	I. II. III. IV. V. VI. VII. VIII. IX. X. XI. XII. XIII. XIV. XV. XVI. XVII. XVIII. XIX. XX. XXI. XXII. XXIII.
*Chenopodiumficifolium* Sm.	arc	XIII.
*Chenopodiumglaucum* L.	ntv/arc	II. III. IV. V. VIII. XI. XIII. XIV. XXIII.
*Chenopodiumhybridum* L.	arc	I. II. III. IV. V. VI. VII. VIII. IX. X. XI. XII. XIII. XIV. XV. XVI. XVII. XVIII. XX. XXI. XXII. XXIII.
*Chenopodiummurale* L.	arc	II. VIII. XI. XIII. XIV.
*Chenopodiumopulifolium* Schrad. ex W.D.J.Koch & Ziz	ntv	I. III. V. VIII. IX. X. XI. XII. XIII. XVI. XX. XXI. XXII. XXIII.
*Chenopodiumpolyspermum* L.	ntv	XIV.
*Chenopodiumrubrum* L.	ntv	IV.
*Chenopodiumstrictum* Roth	arc/neo	II. III. IV. VIII. IX. X. XI. XIV. XV. XVI. XIX. XX.
*Chenopodiumurbicum* L.	arc	VIII.
*Chenopodiumvulvaria* L.	ntv/arc	I. II. III. V. VI. VII. VIII. IX. XI. XII. XIII. XXII.
*Chondrillajuncea* L.	ntv	I. II. III. IV. VI. VIII. IX. X. XI. XII. XIII. XIV. XV. XVI. XIX. XX. XXI. XXII. XXIII.
*Chorisporatenella* DC.	neo	VIII. IX.
*Cichoriumintybus* L.	arc	I. II. III. IV. V. VI. VII. VIII. IX. X. XI. XII. XIII. XIV. XV. XVI. XVII. XVIII. XIX. XX. XXI. XXII. XXIII.
*Cirsiumarvense* (L.) Scop.	arc	I. II. III. IV. V. VI. VII. VIII. IX. X. XI. XII. XIII. XIV. XV. XVI. XVII. XVIII. XX. XXI. XXII. XXIII.
*Cirsiumeriophorum* Scop.	ntv	XI.
*Cirsiumvulgare* (Savi) Ten.	ntv/arc	II. III. V. VI. VIII. IX. X. XI. XII. XIII. XIV. XVI. XXI. XXIII.
*Citrulluslanatus* (Thunb.) Matsum. & Nakai	arc	II. VIII. IX. X. XIII. XV. XVI. XVII. XIX. XXII.
*Clematisvitalba* L.	ntv	I. II. III. V. VII. VIII. IX. X. XI. XII. XIII. XIV. XV. XX. XXI. XXII. XXIII.
*Clerodendrumbungei* Steud.	neo	XVI.
*Clinopodiumacinos* Kuntze	ntv	III. XI. XXI.
*Clinopodiumnepeta* (L.) Kuntze	neo	III. IX. XIII.
*Clinopodiumvulgare* L.	ntv	II. III.
*Coluteaarborescens* L.	ntv	IV. IX.
*Commelinacommunis* L.	neo	I. II. III. IV. V. VI. VII. VIII. IX. X. XI. XIII. XIV. XV. XVI. XIX. XX. XXI. XXII.
*Coniummaculatum* L.	arc	I. III. VI. XV. XXI.
*Convallariamajalis* L.	ntv	I. II. III. VIII. IX. XI. XIV. XV. XVI. XIX. XX. XXII.
*Convolvulusarvensis* L.	arc	I. II. III. IV. V. VI. VII. VIII. IX. X. XI. XII. XIII. XIV. XV. XVI. XVII. XVIII. XIX. XX. XXI. XXII. XXIII.
*Coreopsislanceolata* L.	neo	III.
*Coriandrumsativum* L.	arc	II. VII. XIV.
*Corispermumnitidum* Kit. ex Schult.	ntv	XI.
*Cornusmas* L.	ntv	XII.
*Cornussanguinea* L.	ntv	I. II. III. IV. V. VI. VIII. XI. XII. XIII. XV. XVI. XIX. XX. XXI. XXII. XXIII.
*Coronillavaria* L.	ntv	I. II. III. IV. VI. IX. X. XI. XII. XIII. XIV. XV. XVI. XXI. XXIII.
*Corydaliscava* (L.) Schweigg. & Körte	ntv	I. XI. XII.
*Corydalissolida* (L.) Clairv.	ntv	XI. XII. XV.
*Corylusavellana* L.	ntv	II. III. V. XI. XII. XIII. XV. XVI.
*Coryluscolurna* L.	neo	I. II. III. VIII. XII.
*Cosmosbipinnatus* Cav.	neo	III. XI. XV. XVI. XXI. XXII.
*Cotinuscoggygria* Scop.	ntv	III. XI. XII. XX.
*Cotoneasterhjelmqvistii* Flinck & B.Hylmö	neo	XI.
*Cotoneasterhorizontalis* Decne.	neo	I. II. III. VIII. IX. XI. XII. XVI. XIX. XXII.
*Cotoneastermultiflorus* Bunge	neo	XI.
*Cotoneasterniger* (Wahlb.) Fr.	ntv	I.
*Cotoneasternitens* Rehder & E.H.Wilson	neo	XIX.
*Cotoneastersalicifolius* Franch.	neo	I. III. V.
*Crataeguslaevigata* (Poir.) DC.	ntv	III.
*Crataegusmonogyna* Jacq.	ntv	I. II. III. IV. V. IX. X. XI. XII. XIII. XV. XX. XXI. XXII. XXIII.
*Crepisbiennis* L.	ntv	II. III.
*Crepiscapillaris* (L.) Wallr.	arc	XXI.
Crepisfoetidasubsp.rhoeadifolia (M.Bieb.) Celak.	ntv/arc	II. III. IV. V. VI. VIII. IX. X. XI. XII. XIII. XIV. XV. XVI. XVII. XIX. XX. XXI. XXII. XXIII.
*Crepispulchra* L.	ntv	I. V. XIV.
*Crepissetosa* Haller f.	ntv	VIII. XI. XIV. XX.
*Crepistectorum* L.	arc	V. VI. VIII. IX. X. XI. XIII. XIV. XXI.
*Crocusvernus* (L.) Hill	neo	IV.
*Cucumissativus* L.	arc	XI.
*Cucurbitapepo* L.	neo	XIII.
*Cuscutacampestris* Yunck.	neo	III. IX. X. XI. XII. XIV. XV. XX. XXI. XXII.
*Cuscutaeuropaea* L.	ntv	VIII. IX.
*Cymbalariamuralis* G.Gaertn., B.Mey. & Scherb.	neo	I. II. V. VI. VII. VIII. IX. XI. XII. XIII. XIV. XX.
*Cynodondactylon* (L.) Pers.	arc	I. II. III. IV. V. VI. VII. VIII. IX. X. XI. XII. XIII. XIV. XV. XVI. XVII. XVIII. XIX. XX. XXI. XXII. XXIII.
*Cynoglossumofficinale* L.	ntv	IV. VII.
*Cyperusalternifolius* L.	neo	XIV.
*Cyperuseragrostis* Lam.	neo	III. VIII. X.
*Cyperusfuscus* L.	ntv	III. IV. XXI.
*Cyperusglomeratus* L.	ntv	II. III. XII. XIV.
*Cyperusmichelianus* (L.) Delile	ntv	IV. XXI.
*Cyrtomiumfalcatum* (L.f.) C.Presl	neo	XXI.
*Cyrtomiumfortunei* J.Sm.	neo	VII.
*Cystopterisfragilis* (L.) Bernh.	ntv	VI.
*Dactylisglomerata* L.	ntv	I. II. III. IV. V. VI. VII. VIII. IX. X. XI. XII. XIII. XIV. XV. XVI. XVII. XVIII. XIX. XX. XXI. XXII. XXIII.
Dactylisglomeratasubsp.lobata (Drejer) H.Lindb.	ntv	I. XII.
*Daturainnoxia* Mill.	neo	IV.
*Daturastramonium* L.	arc	I. III. IV. V. VII. VIII. IX. X. XI. XIII. XIV. XV. XX. XXI. XXIII.
*Daturawrightii* Regel	neo	VIII. IX. XI. XIII. XV. XXI. XXII.
*Daucuscarota* L.	arc	I. II. III. IV. V. VI. VIII. IX. X. XI. XII. XIII. XIV. XV. XVI. XVII. XXI. XXII. XXIII.
*Delphiniumajacis* L.	arc	II. III. IX. XI. XV. XVI.
*Delphiniumconsolida* L.	arc/neo	II. III. V. VIII. IX. XI. XIII. XIV. XX. XXI. XXII.
*Deschampsiacaespitosa* P.Beauv.	ntv	IV.
*Descurainiasophia* (L.) Webb ex Prantl	arc	I. II. III. V. VI. VII. VIII. IX. XI. XIII. XIV. XV. XVI. XX. XXIII.
*Deutziascabra* Thunb.	neo	I. III.
Dianthusgiganteiformissubsp.pontederae (A.Kern.) Soó	ntv	XI. XII.
*Digitariaciliaris* (Retz.) Koeler	arc	II. VIII.
*Digitariasanguinalis* (L.) Scop.	arc	I. II. III. IV. V. VI. VII. VIII. IX. X. XI. XII. XIII. XIV. XV. XVI. XVII. XVIII. XIX. XX. XXI. XXII. XXIII.
*Dioscoreapolystachya* Turcz.	neo	I.
*Diplotaxismuralis* DC.	arc	I. II. III. IV. VIII. IX. X. XI. XII. XIII. XIV. XX. XXI. XXIII.
*Diplotaxistenuifolia* (L.) DC.	arc	I. II. III. IV. VIII. XI. XII. XIII. XIV. XV. XVI. XIX. XX. XXI. XXII.
*Drabanemorosa* L.	ntv	XXIII.
*Drabaverna* L.	ntv	I. II. III. IV. IX. XI. XIII. XIV. XV. XVI.
*Dryopteriscarthusiana* (Vill.) H.P.Fuchs	ntv	IX.
Dryopteris filix-mas (L.) Schott	ntv	III. V. VI. VIII. IX. X. XI. XII. XIII. XV.
*Duchesneaindica* (Andrews) Teschem.	neo	I. II. III. IV. V. VI. VIII. IX. XI. XII. XIII. XVI. XXII.
*Dysphaniaambrosioides* (L.) Mosyakin & Clemants	neo	XIII.
*Dysphaniaaristata* (L.) Mosyakin & Clemants	neo	II.
*Dysphaniabotrys* (L.) Mosyakin & Clemants	arc	I. XIV.
*Dysphaniapumilio* (R.Br.) Mosyakin & Clemants	neo	IV. VIII. IX.
*Echinochloacrus-galli* (L.) P.Beauv.	arc	I. II. III. IV. V. VI. VII. VIII. IX. X. XI. XII. XIII. XIV. XV. XVI. XVII. XVIII. XX. XXI. XXII. XXIII.
*Echinopssphaerocephalus* L.	ntv	I. II. III. V. VII. VIII. XI. XII. XIII. XVII.
*Echiumvulgare* L.	arc	I. II. III. IV. VI. VIII. IX. X. XI. XII. XIII. XIV. XV. XVII. XX. XXI. XXIII.
*Ecliptaprostrata* (L.) L.	neo	II. III.
*Elaeagnusangustifolia* L.	neo	III. IV. VIII. IX. X. XI. XII. XV. XVI. XXI. XXIII.
*Eleusineindica* (L.) Gaertn.	neo	I. II. III. IV. V. VI. VII. VIII. IX. X. XI. XII. XIII. XIV. XV. XVI. XVII. XVIII. XIX. XX. XXI. XXII.
*Elymushispidus* (Opiz) Melderis	ntv	III.
*Elymusrepens* (L.) Gould	ntv	I. II. III. IV. V. VI. VII. VIII. IX. X. XI. XII. XIII. XIV. XV. XVI. XVII. XVIII. XIX. XX. XXI. XXII. XXIII.
*Epilobiumangustifolium* L.	ntv	IX.
*Epilobiumciliatum* Raf.	neo	XIV.
*Epilobiumdodonaei* Vill.	ntv	XIII.
*Epilobiumhirsutum* L.	ntv	II. XVII. XXIII.
*Epilobiumparviflorum* (Schreb.) Schreb.	ntv	I. III. V. VIII. IX. XIV. XXI.
*Epilobiumtetragonum* L.	ntv	II. IV. V. VIII. IX. X. XI. XII. XIV. XV. XXI.
*Epimediumalpinum* L.	neo	VIII.
*Epipactishelleborine* (L.) Crantz	ntv	IV.
*Equisetumarvense* L.	ntv	III. V. VI. XI. XXIII.
*Equisetumhyemale* L.	ntv	III. VIII.
*Equisetummoorei* Newman	ntv	III.
*Equisetumramosissimum* Desf.	ntv	II. III. VIII. IX. XI. XIII. XIV. XX. XXI. XXIII.
*Eragrostisminor* Host	arc	I. II. III. IV. V. VI. VII. VIII. IX. X. XI. XII. XIII. XIV. XV. XVI. XVII. XVIII. XIX. XX. XXI. XXII. XXIII.
*Eragrostispilosa* (L.) P.Beauv.	arc	III. VIII. IX. XIV. XV.
*Eragrostisspectabilis* (Pursh) Steud.	neo	II.
*Eragrostisvirescens* J.Presl	neo	I. XIV.
*Eranthishyemalis* Salisb.	arc/neo	III. XII. XV.
*Erigeronacris* L.	ntv	XXIII.
*Erigeronannuus* (L.) Pers.	neo	I. II. III. IV. V. VI. VII. VIII. IX. X. XI. XII. XIII. XIV. XV. XVI. XVII. XVIII. XIX. XX. XXI. XXII. XXIII.
*Erigeroncanadensis* L.	neo	I. II. III. IV. V. VI. VII. VIII. IX. X. XI. XII. XIII. XIV. XV. XVI. XVII. XVIII. XIX. XX. XXI. XXII. XXIII.
*Erigeronsumatrensis* Retz.	neo	III. IX. XI. XIV. XV. XXI.
*Erodiumcicutarium* (L.) L'Hér.	arc	I. II. III. IV. V. VI. VII. VIII. IX. X. XI. XII. XIII. XIV. XV. XVI. XVII. XVIII. XIX. XX. XXI. XXII. XXIII.
*Erucavesicaria* (L.) Cav.	neo	XIII.
*Eryngiumcampestre* L.	ntv	II. III. VIII. XI. XII. XIV. XX. XXI. XXIII.
*Erysimumdiffusum* Ehrh.	arc	VI. XIII.
*Erysimumodoratum* Ehrh.	ntv	VIII. XI. XII.
*Erysimumrepandum* L.	arc	XII.
*Eschscholziacalifornica* Cham.	neo	V. XIV.
*Euonymuseuropaeus* L.	ntv	II. IV. VIII. XI. XII. XIII. XIX. XXII.
*Euonymusjaponicus* Thunb.	neo	III. IV. XXII.
*Euonymusverrucosus* Scop.	ntv	XI. XII.
*Eupatoriumcannabinum* L.	ntv	II. IV. VIII. XIII.
*Euphorbiaamygdaloides* L.	ntv	XVI.
*Euphorbiacyparissias* L.	ntv/arc	III. IV. IX. XI. XII. XIII. XIV. XV. XIX. XXI. XXIII.
*Euphorbiaesula* L.	ntv/arc	III. XII. XXI. XXIII.
*Euphorbiafalcata* L.	arc/neo	IX. XII. XIV.
*Euphorbiaglareosa* Pall. ex M.Bieb.	ntv	XI.
*Euphorbiahelioscopia* L.	arc	III. VI. VIII. XI. XIV. XV.
*Euphorbiahumifusa* Willd.	neo	XI.
*Euphorbialathyris* L.	arc	II. III. IX. XI. XII. XIII. XIV. XV. XVI. XIX. XXII.
*Euphorbiamaculata* L.	neo	I. II. III. IV. V. VI. VII. VIII. IX. X. XI. XII. XIII. XIV. XV. XVI. XVII. XVIII. XIX. XX. XXI. XXII. XXIII.
*Euphorbiamarginata* Pursh	neo	III. XV. XVI. XXI.
*Euphorbiamyrsinites* L.	neo	III. XI. XII. XIV. XVI. XXII.
*Euphorbiapeplus* L.	arc	I. II. III. IV. V. VI. VII. VIII. IX. X. XI. XII. XIII. XIV. XV. XVI. XVII. XVIII. XIX. XX. XXII. XXIII.
*Euphorbiaprostrata* Aiton	neo	II. III. IV. VI. VIII. IX. XI. XIV. XV. XVI.
*Euphorbiasalicifolia* Host	ntv	XI.
*Euphorbiaserpens* Kunth	neo	III. XIV.
*Euphorbiataurinensis* All.	arc	VIII. XI. XII. XIII. XIV.
*Euphorbiavirgata* Waldst. & Kit.	ntv	XI.
*Fagopyrumesculentum* Moench	neo	IV. IX. XIV.
*Falcariavulgaris* Bernh.	ntv	I. II. III. VIII. IX. X. XI. XII. XIII. XV. XVI. XX. XXI.
*Fallopiaaubertii* (L.Henry) Holub	neo	XXII.
*Fallopiabaldschuanica* (Regel) Holub	neo	I. XI. XII. XIV. XIX.
*Fallopiaconvolvulus* (L.) Á.Löve	arc	I. II. III. IV. V. VII. VIII. IX. X. XI. XII. XIII. XIV. XVI. XVII. XIX. XX. XXI. XXIII.
*Fallopiadumetorum* (L.) Holub	arc	III. XII. XV.
*Festucapseudovina* Hack. ex Wiesb.	ntv	III. VIII. IX. X. XI. XIV. XXIII.
*Festucarubra* L.	ntv	II. III. VI. VIII. IX. X. XI. XII. XIV. XVII.
*Festucarupicola* Heuff.	ntv	III. IV. IX. X. XI. XIII. XX. XXI. XXIII.
*Festucavalesiaca* Schleich. ex Gaudin	ntv	II. IX. X. XI. XVI.
*Ficuscarica* L.	arc/neo	III.
*Filagogermanica* (L.) Huds.	ntv	XIV.
*Forsythiaintermedia* Zabel	neo	XV.
*Fragariaananassa* (Duchesne ex Weston) Duchesne ex Rozier	neo	II. XX.
*Fragariavesca* L.	ntv	II. VIII. XI. XII.
*Fragariaviridis* Weston	ntv	II.
*Fraxinusangustifolia* Vahl	ntv	I. III. VIII. X. XIII.
*Fraxinusexcelsior* L.	ntv	I. II. III. V. VI. VIII. IX. XI. XII. XIII.
*Fraxinusornus* L.	ntv	I. II. III. VIII. XI. XII. XIII. XIV. XV.
*Fraxinuspennsylvanica* Marshall	neo	II. IV. V. VIII. IX. X. XI. XIII. XX. XXI.
*Fumariaschleicheri* Soy.-Will.	arc	II. XI. XII. XIV.
*Fumariavaillantii* Loisel.	arc	I. II. III. VI. VIII. IX. XI. XII. XIV. XV. XX.
*Gageapratensis* (Pers.) Dumort.	ntv	XV.
*Gageavillosa* (M.Bieb.) Sweet	ntv	I. XI.
*Gaillardiagrandiflora* Hort. ex Van Houtte	neo	XVI.
*Galanthuselwesii* Hook.f.	neo	XI. XII. XV.
*Galanthusnivalis* L.	ntv	III. VIII. XV.
*Galinsogaparviflora* Cav.	neo	I. II. III. IV. V. VI. VII. VIII. IX. X. XI. XII. XIII. XIV. XV. XVI. XIX. XX. XXI.
*Galinsogaquadriradiata* Ruiz & Pav.	neo	I. II. V. VIII. IX. XI. XIII. XIV.
*Galiumalbum* Mill.	ntv	II.
*Galiumaparine* L.	arc	I. II. III. IV. V. VI. VII. VIII. IX. X. XI. XII. XIII. XIV. XV. XVI. XX. XXI. XXII. XXIII.
*Galiumglaucum* L.	ntv	XI.
*Galiummollugo* L.	ntv	II. III. IV. V. VI. IX. X. XI. XII. XIII. XV. XVII. XX. XXIII.
*Galiumpalustre* L.	ntv	VI.
*Galiumspurium* L.	arc	VIII.
*Galiumverum* L.	ntv	III. IX. XI. XIV. XXIII.
*Gazaniarigens* (L.) Gaertn.	neo	IV.
*Geraniummacrorrhizum* L.	neo	III. IX. XIII. XV.
*Geraniummolle* L.	arc	XI. XVI.
*Geraniumpurpureum* Vill.	neo	III. VIII. X. XIV.
*Geraniumpusillum* L.	arc	I. II. III. IV. V. VI. VII. VIII. IX. X. XI. XII. XIII. XIV. XV. XVI. XVIII. XIX. XX. XXI. XXII. XXIII.
*Geraniumpyrenaicum* Burm.f.	neo	II. III. XII. XV. XX.
*Geraniumrobertianum* L.	ntv/arc	VII. IX. X. XI. XII. XIII.
*Geraniumrotundifolium* L.	ntv	VIII. XI.
*Geraniumsanguineum* L.	ntv	IX.
*Geraniumsibiricum* L.	neo	VIII.
*Geumcoccineum* Sm.	neo	XXI.
*Geumurbanum* L.	ntv	I. II. III. IV. V. VIII. IX. X. XI. XII. XIII. XIV. XV. XVI. XVII. XIX. XX. XXI. XXII.
*Ginkgobiloba* L.	neo	VIII. XIII.
*Glebioniscoronaria* (L.) Cass. ex Spach	neo	VI.
*Glechomahederacea* L.	ntv	I. II. III. IV. V. VI. VIII. IX. X. XI. XII. XIII. XIV. XV. XVI. XVII. XX. XXI. XXII. XXIII.
*Glechomahirsuta* Waldst. & Kit.	ntv	XI.
*Gleditsiatriacanthos* L.	neo	II. III. IV. V. VIII. IX. X. XI. XII. XIII. XIV. XIX. XX. XXI. XXII.
*Glyceriamaxima* (Hartm.) Holmb.	ntv	XX.
*Gnaphaliumuliginosum* L.	ntv	IX.
*Gymnocarpiumrobertianum* (Hoffm.) Newman	ntv	IV. V. IX. XII. XIII.
*Gypsophilapaniculata* L.	ntv	IV. IX. XIII. XV.
*Hederahelix* L.	ntv	I. II. III. IV. V. VI. VII. VIII. IX. X. XI. XII. XIII. XIV. XV. XVI. XVII. XVIII. XIX. XX. XXI. XXII. XXIII.
*Helianthemumovatum* Dunal	ntv	II. XII.
*Helianthusannuus* L.	neo	III. V. IX. XVI. XXI.
*Helianthustuberosus* L.	neo	II. III. XI. XIII. XIV. XV. XIX. XXII.
*Heliopsishelianthoides* Sweet	neo	XIV. XVII. XVIII. XIX.
*Heliotropiumeuropaeum* L.	arc	IV. XIX.
*Helminthothecaechioides* (L.) Holub	arc/neo	III.
*Hemerocallisfulva* (L.) L.	neo	III. XII. XV. XVI.
*Heracleumsphondylium* L.	ntv	II. XII.
*Herniariaglabra* L.	ntv	VIII.
*Herniariahirsuta* L.	arc	II. III. VIII. XIV.
*Hibiscussyriacus* L.	neo	I. IV. V. VIII. IX. XI. XIII. XIV. XV. XVI. XIX. XXII.
*Hibiscustrionum* L.	arc	II. III. IV. VI. VIII. IX. X. XI. XIV. XV. XXI.
*Hieraciummurorum* L.	ntv	XII.
*Hieraciumsabaudum* L.	ntv	I. III. XI. XII.
*Hieraciumumbellatum* L.	ntv	III. XII.
*Hippophaerhamnoides* L.	ntv	XI. XIV.
*Holcuslanatus* L.	ntv	III. XI. XIV.
*Holosteumumbellatum* L.	ntv	I. III. IX. X. XI. XII. XIII. XIV. XV. XVI.
*Hordelymuseuropaeus* (L.) Jess. ex Harz	ntv	XII.
*Hordeummurinum* L.	arc	I. II. III. IV. V. VI. VII. VIII. IX. X. XI. XII. XIII. XIV. XV. XVI. XVII. XVIII. XIX. XX. XXI. XXII. XXIII.
*Hordeumvulgare* L.	arc	II. V. VI. VIII. XIII. XX.
*Hornungiapetraea* Rchb.	ntv	V.
*Hostaplantaginea* (Lam.) Asch.	neo	XVI.
*Humuluslupulus* L.	ntv	I. II. III. IV. VIII. IX. X. XI. XII. XIII. XIV. XV. XVI. XVII. XX. XXI. XXII. XXIII.
*Hyacinthusorientalis* L.	neo	XV.
*Hylotelephiumspectabile* (Boreau) H.Ohba	neo	XII. XIII.
*Hylotelephiumtelephium* (L.) H.Ohba	ntv	XI. XV.
*Hyoscyamusniger* L.	arc	XIV.
*Hypericumcalycinum* L.	neo	VIII. XI. XIV. XV.
*Hypericumperforatum* L.	ntv	I. II. III. IV. IX. X. XI. XII. XIV. XVI. XX. XXI. XXIII.
*Hypochaerisradicata* L.	ntv	I. II. III. IV. V. VI. VIII. IX. X. XI. XII. XIII. XIV. XV. XVI. XVII. XVIII. XIX. XX. XXI. XXII. XXIII.
*Hyssopusofficinalis* L.	neo	II.
*Iberissempervirens* L.	neo	XV.
*Impatiensbalfourii* Hook.f.	neo	III. XVI. XXI.
*Impatiensbalsamina* L.	neo	XI.
*Impatiensparviflora* DC.	neo	XII.
*Impatienswalleriana* Hook.f.	neo	VIII. IX.
*Inulabritannica* L.	ntv	III. V. VIII. IX. X. XI. XIII. XIV. XXIII.
*Inulaconyza* DC.	ntv	III. XII. XXI.
Inulaoculus-christi L.	ntv	XI.
*Ipomoeapurpurea* (L.) Roth	neo	I. II. IV. VIII. X. XI. XIII. XV. XIX. XXII.
*Irisgermanica* L.	arc	III. XI. XV. XVI.
*Irispseudacorus* L.	ntv	IX.
*Isatistinctoria* L.	arc	III.
*Jacobaeavulgaris* Gaertn.	ntv	III. IV. IX. XIV. XXI. XXII. XXIII.
*Jasminumnudiflorum* Lindl.	neo	III. VIII.
*Juglansregia* L.	arc	I. II. III. IV. VIII. IX. X. XI. XII. XIII. XIV. XV. XVI. XVII. XIX. XXI. XXII. XXIII.
*Juncusarticulatus* L.	ntv	XIV. XXIII.
*Juncusbufonius* L.	ntv	VIII.
*Juncuscompressus* Jacq.	ntv	III. IV. VIII. IX. XI. XII. XIII. XIV. XX.
*Juncuseffusus* L.	ntv	VIII.
*Juncusinflexus* L.	ntv	XI.
*Juniperusvirginiana* L.	neo	XII. XV.
*Kickxiaelatine* (L.) Dumort.	arc	XI.
*Kickxiaspuria* (L.) Dumort.	arc	IV.
*Knautiaarvensis* (L.) Coult.	ntv	III. XI.
*Koeleriacristata* Pers.	ntv	X. XI.
*Koelreuteriapaniculata* Laxm.	neo	I. II. III. IV. V. VI. VII. VIII. IX. X. XI. XII. XIII. XIV. XV. XVI. XVII. XVIII. XIX. XX. XXI. XXIII.
*Laburnumanagyroides* Medik.	arc/neo	II. XII.
*Lactucamuralis* (L.) Gaertn.	ntv	V. XII. XIII.
*Lactucasaligna* L.	ntv/arc	III. IV. VIII. XIII. XIV. XV. XVII. XX. XXII.
*Lactucaserriola* L.	arc	I. II. III. IV. V. VI. VII. VIII. IX. X. XI. XII. XIII. XIV. XV. XVI. XVII. XVIII. XIX. XX. XXI. XXII. XXIII.
*Lagenariasiceraria* (Molina) Standl.	neo	XI.
*Lamiumamplexicaule* L.	arc	I. II. III. IV. V. VI. VII. VIII. IX. X. XI. XII. XIII. XIV. XV. XVI. XX. XXI. XXIII.
*Lamiumgaleobdolon* (L.) L.	ntv	XII.
Lamiumgaleobdolonsubsp.argentatum (Smejkal) J.Duvign.	neo	XII.
*Lamiummaculatum* L.	ntv	XI. XII. XV.
*Lamiumpurpureum* L.	arc	I. II. III. VII. VIII. IX. XI. XII. XIII. XIV. XV. XVI.
*Lappulasquarrosa* Dumort.	arc	II. VIII. IX. XIII.
*Lapsanacommunis* L.	ntv	I. II. VIII. XI. XII. XIII.
*Lathyruslatifolius* L.	ntv	XII.
*Lathyrustuberosus* L.	arc	I. II. V. IX. XI. XII. XIV. XV. XXIII.
*Lavandulaangustifolia* Mill.	neo	III. XVI.
*Lemnaminor* L.	ntv	XVII.
*Leontodonhispidus* L.	ntv	I. II. III. XI. XII. XIII. XIV. XV.
*Leontodonsaxatilis* Lam.	ntv	I. II. III. IV. V. XI. XIII. XIV. XXIII.
*Leopoldiacomosa* (L.) Parl.	ntv/arc	IX.
*Lepidiumcampestre* (L.) W.T.Aiton	ntv/arc	III. IX. XI. XII.
*Lepidiumdensiflorum* Schrad.	neo	II. VII. VIII. IX. XI. XIII. XIV. XX.
*Lepidiumdidymum* L.	neo	XIV.
*Lepidiumdraba* L.	ntv/arc	I. II. III. IV. VI. VIII. IX. X. XI. XII. XIII. XIV. XV. XVI. XX. XXI. XXII. XXIII.
*Lepidiumperfoliatum* L.	arc	XIV.
*Lepidiumruderale* L.	arc	I. II. III. IV. V. VI. VII. VIII. IX. X. XI. XII. XIII. XIV. XV. XVI. XXII.
*Lepidiumvirginicum* L.	neo	III. IV. VI. XXI.
Leucanthemumadustumsubsp.margaritae (Gáyer) Holub	ntv	XVI.
*Leymusarenarius* (L.) Hochst.	neo	I. III. XIX.
*Ligustrumovalifolium* Hassk.	neo	XVI. XIX.
*Ligustrumvulgare* L.	ntv	I. II. III. V. VI. VII. VIII. IX. XI. XII. XV. XVI. XIX. XX. XXI.
*Limoniumgmelinii* Kuntze	ntv	III. XI.
*Linariagenistifolia* (L.) Mill.	ntv	I. X. XI. XII. XXI. XXIII.
*Linariamaroccana* Hook.f.	neo	VI. VIII.
*Linariavulgaris* Mill.	ntv	I. III. IV. VI. VIII. IX. X. XI. XII. XIII. XIV. XV. XVII. XXI. XXII. XXIII.
*Linumaustriacum* L.	ntv	III. VI. IX. XI. XV. XVI.
*Linumperenne* L.	ntv	VIII. IX. X.
*Lithospermumofficinale* L.	ntv	IX.
*Lobulariamaritima* (L.) Desv.	neo	V. VIII. IX. XI. XIII. XIV. XV. XIX. XX. XXII.
*Loliummultiflorum* Lam.	arc/neo	II. III. XI. XIV. XXI.
*Loliumperenne* L.	ntv/arc	I. II. III. IV. V. VI. VII. VIII. IX. X. XI. XII. XIII. XIV. XV. XVI. XVII. XVIII. XIX. XX. XXI. XXII. XXIII.
*Loliumpratense* (Huds.) Darbysh.	ntv	I. II. III. IV. V. VI. VIII. IX. X. XI. XII. XIII. XIV. XV. XVI. XVII. XIX. XXI. XXII. XXIII.
*Lonicerajaponica* Thunb.	neo	III. XIV.
*Loniceramaackii* (Rupr.) Maxim.	neo	XI.
*Loniceranitida* E.H.Wilson	neo	I. II. III. VIII. XI. XII. XIII. XV.
*Loniceratatarica* L.	neo	V. VI. XI.
*Loniceraxylosteum* L.	ntv	I. II. VI. X. XI. XII. XIII. XIV. XVI.
*Lotusborbasii* Ujhelyi	ntv	XI.
*Lotuscorniculatus* L.	ntv	I. II. III. IV. IX. XI. XII. XIII. XIV. XV. XX. XXI. XXII. XXIII.
*Lotusmaritimus* L.	ntv	XV.
*Lotustenuis* Waldst. & Kit.	ntv	II. III. XI.
*Lunariaannua* L.	arc/neo	IX. XII. XV.
*Lyciumbarbarum* L.	arc	I. II. III. IV. VIII. IX. X. XI. XII. XIII. XIV. XIX. XXI. XXII. XXIII.
*Lycopuseuropaeus* L.	ntv	XVII. XXI.
*Lysimachiaarvensis* (L.) U.Manns & Anderb.	arc	II. III. IV. V. VI. VIII. IX. X. XI. XII. XIII. XIV. XV. XVI. XXII. XXIII.
*Lysimachiaciliata* L.	neo	II.
*Lysimachianummularia* L.	ntv	II. III. IV. VIII. IX. XI. XII. XVI.
*Lysimachiapunctata* L.	ntv	I. XX.
*Lysimachiavulgaris* L.	ntv	III.
*Lythrumsalicaria* L.	ntv	II. III. IX.
*Macleayacordata* (Willd.) R.Br.	neo	XI.
*Maclurapomifera* (Raf.) C.K.Schneid.	neo	V. XI.
*Malusdomestica* (Suckow) Borkh.	arc	I. IX. X.
*Malussylvestris* (L.) Mill.	ntv	IV.
*Malvaneglecta* Wallr.	arc	I. II. III. IV. V. VI. VII. VIII. IX. X. XI. XII. XIII. XIV. XV. XVI. XVIII. XIX. XX. XXI. XXII. XXIII.
*Malvasylvestris* L.	arc	I. II. III. IV. V. VI. VIII. IX. X. XI. XII. XIII. XIV. XV. XVI. XVII. XX. XXI. XXII. XXIII.
*Marrubiumperegrinum* L.	arc	XXII.
*Matricariachamomilla* L.	ntv	II. IX. XI. XIV. XXI.
*Matricariadiscoidea* DC.	neo	II. V. XII. XIII. XIV. XV.
Matthiolalongipetalasubsp.bicornis Ball	neo	VII.
*Medicagofalcata* L.	ntv	II. III. IX. X. XI. XII. XIII. XIV. XV. XVI. XIX. XXI. XXII. XXIII.
*Medicagolupulina* L.	ntv	I. II. III. IV. V. VI. VII. VIII. IX. X. XI. XII. XIII. XIV. XV. XVI. XVII. XVIII. XIX. XX. XXI. XXII. XXIII.
*Medicagominima* (L.) L.	ntv	II. IV. VI. VIII. X. XI. XII. XIII. XIV. XV.
*Medicagomonspeliaca* (L.) Trautv.	ntv	III. XIV.
*Medicagosativa* L.	arc	I. II. III. IV. V. VI. VIII. IX. X. XI. XII. XIII. XIV. XV. XVI. XIX. XX. XXI. XXII. XXIII.
*Medicagovaria* Martyn	arc	II. III. XI. XII. XIII. XIV. XV.
*Melampyrumnemorosum* L.	ntv	XI.
*Melicaciliata* L.	ntv	I. IX.
*Melicatranssilvanica* Schur	ntv	I. IV. XI. XII. XIII. XIV. XIX. XXIII.
*Melicauniflora* Retz.	ntv	XII.
*Melilotusalbus* Medik.	arc	V. VIII. XIV. XX.
*Melilotusofficinalis* (L.) Pall.	arc	I. II. III. IV. V. VI. VII. VIII. IX. X. XI. XII. XIII. XIV. XV. XVI. XVII. XX. XXI. XXIII.
*Melissaofficinalis* L.	arc	I. II. XI. XII. XIV. XV. XVI. XIX. XXII.
*Menthaaquatica* L.	ntv	II. V. VIII. XI. XIII. XIX. XXI. XXIII.
*Menthalongifolia* (L.) L.	ntv	II. VIII. XIII. XIV. XX. XXII.
*Menthapulegium* L.	ntv	V.
*Menthaspicata* L.	neo	III. VIII. IX. XI. XIII. XV. XVI. XXII.
*Mercurialisannua* L.	arc	II. III. VI. IX. X. XI. XII. XXII.
*Mercurialisperennis* L.	ntv	III. XII.
*Minuartiaglomerata* (M.Bieb.) Degen	ntv	V. XI.
*Mirabilisjalapa* L.	neo	III. XI. XII. XV. XVI.
*Miscanthussinensis* Andersson	neo	VIII. IX. XX.
*Morusalba* L.	arc	I. II. III. IV. V. VI. VII. VIII. IX. X. XI. XII. XIII. XIV. XV. XVI. XVII. XVIII. XIX. XX. XXI. XXII. XXIII.
*Morusnigra* L.	arc	XI. XV. XVII.
*Muscarineglectum* Guss. ex Ten.	ntv/arc	I. II. III. VIII. IX. X. XI. XII. XIII. XIV. XV. XVI. XVII.
*Myosotisarvensis* Hill	arc	II. VI. IX.
*Myosotisstricta* Link ex Roem. & Schult.	ntv	X.
*Nassellatenuissima* (Trin.) Barkworth	neo	I. II. XIII. XIV.
*Nepetacataria* L.	arc	IX.
*Nepetaracemosa* Lam.	neo	II. IV. V. IX. XI. XII. XIII. XIV. XVI.
*Nicotianaalata* Link & Otto	neo	IX.
*Nicotianasylvestris* Speg.	neo	XXI.
*Nonealutea* DC.	neo	VIII.
*Odontitesvernus* (Bellardi) Dumort.	ntv	X.
*Oenotherabiennis* L.	neo	I. IV. VIII. IX. X. XI. XIII. XXI. XXIII.
*Oenotheragaura* W.L.Wagner & Hoch	neo	XIV.
*Oenotheraglazioviana* Micheli	neo	XV.
*Ononisspinosa* L.	ntv	XXI.
*Onopordumacanthium* L.	arc	III. X. XI. XIV. XV. XX. XXI. XXIII.
*Origanumvulgare* L.	ntv	II. III. IX. X.
*Orlayagrandiflora* (L.) Hoffm.	arc	V.
*Ornithogalumboucheanum* (Kunth) Asch.	ntv	XV.
*Ornithogalumrefractum* Kit. ex Schltdl.	ntv/arc	I. XI. XV. XVI.
*Ornithogalumumbellatum* L.	ntv/arc	XVI.
*Oxaliscorniculata* L.	neo	I. II. III. IV. V. VI. VII. VIII. IX. X. XI. XII. XIII. XIV. XV. XVI. XVII. XVIII. XX. XXI. XXII. XXIII.
*Oxalisdillenii* Jacq.	neo	I. II. III. IV. V. VI. VII. VIII. IX. X. XI. XII. XIII. XIV. XV. XVI. XVII. XVIII. XIX. XX. XXI. XXII. XXIII.
*Oxybaphusnyctagineus* (Michx.) Sweet	neo	III. IV. V. VI. VIII. IX. X. XIII. XIV. XV. XX. XXI.
*Pachysandraterminalis* Siebold & Zucc.	neo	I.
*Panicumcapillare* L.	neo	I. III. VIII. IX. XI. XXI. XXIII.
*Panicummiliaceum* L.	arc	VIII. XIX.
Panicummiliaceumsubsp.ruderale (Kitag.) Tzvelev	neo	XIX.
*Panicumriparium* H.Scholz	neo	III. XI.
*Panicumvirgatum* L.	neo	III. IX.
*Papaverdubium* L.	arc	I. II. III. V. VI. XI. XIII. XIV. XV.
*Papaverrhoeas* L.	arc	I. II. III. IV. V. VI. VII. VIII. IX. X. XI. XII. XIII. XIV. XV. XVI. XVII. XX.
*Papaversomniferum* L.	arc	II. III.
*Parietariajudaica* L.	neo	II. VIII.
*Parietariaofficinalis* L.	ntv	I. II. III. IV. V. VI. VII. VIII. IX. XI. XII. XIII. XIV. XX. XXI. XXII.
*Parthenocissusinserta* (A.Kern.) Fritsch	neo	I. II. III. V. VI. VII. VIII. IX. X. XI. XII. XIII. XIV. XV. XVI. XX. XXI. XXII.
*Parthenocissustricuspidata* Planch.	neo	I. III. XI. XIII.
*Pastinacasativa* L.	ntv	III. XI. XII. XIII. XV. XVII. XXIII.
*Paulowniatomentosa* (Thunb.) Steud.	neo	II. IV. V. VIII. X. XI. XIX.
*Perillafrutescens* (L.) Britton	neo	VIII. XIX.
*Periplocagraeca* L.	neo	I. V. IX. XI. XII. XV.
*Perovskiaatriplicifolia* Benth.	neo	II. III. V. VIII. XI. XIII. XIV. XXI.
*Persicariaamphibia* (L.) Delarbre	ntv	X.
*Persicariadubia* Fourr.	ntv	XIII. XIV.
*Persicariahydropiper* (L.) Delarbre	ntv	III.
*Persicarialapathifolia* (L.) Delarbre	arc	II. V. VI. VII. VIII. IX. XII. XIII. XV. XVII. XXIII.
*Persicariamaculosa* Gray	arc	II. III. IV. VI. VII. VIII. XI. XII. XIII. XIV. XV. XVI. XXI. XXII.
*Persicariaminor* Opiz	ntv	XVII.
*Persicariaorientalis* (L.) Spach	neo	XVI.
*Petrorhagiaprolifera* (L.) P.W.Ball & Heywood	ntv	IV. IX. X. XI. XV. XX. XXI.
*Petrorhagiasaxifraga* Link	ntv	X. XV.
*Petrosedumrupestre* (L.) P.V.Heath	neo	I. II. III. VIII. IX. XI. XV.
*Petroselinumcrispum* (Mill.) Fuss	arc/neo	IX. XI.
*Petuniahybrida* E.Vilm.	neo	I. IV. V. VIII. IX. XI. XIII. XIV. XV. XIX. XX.
*Peucedanumalsaticum* L.	ntv	III.
*Peucedanumcervaria* (L.) Lapeyr.	ntv	III.
*Peucedanumoreoselinum* Moench	ntv	IV.
*Phalarisarundinacea* L.	ntv	IX.
*Phalariscanariensis* L.	neo	III.
*Phedimuskamtschaticus* (Fisch. & C.A.Mey.) 't Hart	neo	XIV.
*Philadelphuscoronarius* L.	neo	XI. XII. XV.
*Phleumphleoides* (L.) H.Karst.	ntv	III.
*Phleumpratense* L.	ntv	XXIII.
*Phragmitesaustralis* (Cav.) Trin. ex Steud.	ntv	II. III. IV. VIII. IX. X. XI. XIII. XIV. XX. XXI. XXIII.
*Physalisperuviana* L.	neo	XIX. XXII.
*Phytolaccaamericana* L.	neo	II. VIII. IX. XIII. XVI. XXII.
*Phytolaccaesculenta* Van Houtte	neo	I. III. IV. V. VI. VII. VIII. IX. XI. XII. XIII. XIV. XV. XVI. XVII. XIX. XXI.
*Piceaabies* (L.) H.Karst.	ntv/arc	III. IX. XII.
*Picrishieracioides* Sm.	arc	II. III. IV. V. VIII. IX. X. XI. XII. XIII. XIV. XV. XVI. XXI. XXII. XXIII.
*Pileamicrophylla* (L.) Liebm.	neo	IX.
*Pilosellabauhini* (Schult.) Arv.-Touv.	ntv	III. IV. IX. XII. XIII. XV. XXIII.
*Pilosellacaespitosa* (Dumort.) P.D.Sell & C.West	ntv	XIV.
*Pilosellaofficinarum* Vaill.	ntv	II. III. IV. VI. IX. XI. XII. XIII. XIV. XV. XXI. XXII. XXIII.
*Pilosellapiloselloides* (Vill.) Soják	ntv	XI.
*Pimpinellasaxifraga* L.	ntv	I. V. VIII. IX. XII. XIII. XV. XVI.
*Pinusnigra* J.F.Arnold	neo	IX. XII.
*Pinussylvestris* L.	ntv	IV.
*Plantagoaltissima* L.	ntv	III.
*Plantagocoronopus* L.	neo	VII.
*Plantagoindica* L.	ntv	IV. IX. XIII. XX.
*Plantagolanceolata* L.	ntv	I. II. III. IV. V. VI. VII. VIII. IX. X. XI. XII. XIII. XIV. XV. XVI. XVII. XVIII. XIX. XX. XXI. XXII. XXIII.
*Plantagomajor* L.	ntv	I. II. III. IV. V. VI. VII. VIII. IX. X. XI. XII. XIII. XIV. XV. XVI. XVII. XVIII. XIX. XX. XXI. XXII. XXIII.
*Plantagomaritima* L.	ntv	XI.
*Plantagomedia* L.	ntv	II. VIII. XII.
*Platanusacerifolia* (Aiton) Willd.	neo	I. V. VI. VIII. IX. X. XI. XIII. XIV. XIX.
*Platycladusorientalis* (L.) Franco	neo	III. IX. XII. XV. XXII.
*Platycodongrandiflorus* A.DC.	neo	XXI.
*Poaangustifolia* L.	ntv	I. II. III. IV. V. VI. VII. VIII. IX. X. XI. XII. XIII. XIV. XV. XVI. XVII. XX. XXI. XXIII.
*Poaannua* L.	ntv	I. II. III. IV. V. VI. VII. VIII. IX. X. XI. XII. XIII. XIV. XV. XVI. XX. XXI. XXII. XXIII.
*Poabulbosa* L.	ntv	I. II. III. VI. VIII. XI. XIII. XIV. XV. XVI. XVII.
*Poacompressa* L.	ntv	II. III. IV. IX. X. XI. XII. XIV. XX.
*Poanemoralis* L.	ntv	II. XI. XII.
*Poapalustris* L.	ntv	III.
*Poapratensis* L.	ntv	I. II. III. VI. VIII. IX. X. XI. XII. XIII. XIV. XV. XVI. XVII. XX. XXI. XXIII.
*Poatrivialis* L.	ntv	II.
*Podospermumcanum* C.A.Mey.	ntv	IV. IX. XI. XXIII.
*Polycarpontetraphyllum* (L.) L.	neo	III. VII. VIII. XIV. XV.
*Polygonatumlatifolium* (Jacq.) Desf.	ntv	II. XII.
*Polygonatumodoratum* (Mill.) Druce	ntv	XV. XVI.
*Polygonumaviculare* L.	arc	I. II. III. IV. V. VI. VII. VIII. IX. X. XI. XII. XIII. XIV. XV. XVI. XVII. XVIII. XIX. XX. XXI. XXII. XXIII.
*Polygonumrurivagum* Jord. ex Boreau	arc	IV.
*Polypodiumvulgare* L.	ntv	VIII.
*Polypogonviridis* (Gouan) Breistr.	neo	II.
*Polystichumaculeatum* (L.) Roth	ntv	VIII. XI. XXI.
*Polystichumsetiferum* Rosendahl	ntv	XXI.
*Populusalba* L.	ntv	I. II. III. IV. V. VIII. IX. X. XI. XIII. XVI. XIX. XXI. XXII. XXIII.
*Populuscanescens* (Aiton) Sm.	ntv	I. III. VI. XI. XIII. XIV. XV. XX.
*Populuseuramericana* (Dode) Guinier	neo	III. XVI.
*Populusnigra* L.	ntv	I. II. III. IV. V. VIII. IX. X. XI. XII. XIII. XIV. XV. XVI. XX. XXI. XXII. XXIII.
*Populussimonii* Carrière	neo	VIII. X. XI. XIII.
*Populustremula* L.	ntv	X. XIII.
*Portulacagrandiflora* Hook.	neo	I. III. VIII. IX. X. XIII. XIV. XV. XVI. XX. XXII.
*Portulacaoleracea* L.	arc	I. II. III. IV. V. VI. VII. VIII. IX. X. XI. XII. XIII. XIV. XV. XVI. XVII. XVIII. XIX. XX. XXI. XXII. XXIII.
*Potentillaanserina* L.	arc	V. VI. IX. X. XII. XIII. XIV. XXI. XXIII.
*Potentillaargentea* L.	ntv	I. II. III. IV. V. VII. VIII. IX. X. XI. XII. XIII. XIV. XV. XVI. XVII. XVIII. XIX. XX. XXI. XXII. XXIII.
*Potentillaincana* G.Gaertn., B.Mey. & Scherb.	ntv	VIII.
*Potentillarecta* L.	ntv	II. III. XI. XII. XIV. XVI. XXI.
*Potentillareptans* L.	ntv	I. II. III. IV. V. VI. VII. VIII. IX. X. XI. XII. XIII. XIV. XV. XVI. XVII. XVIII. XX. XXI. XXII. XXIII.
*Potentillasupina* L.	ntv	I. III. IV. V. VI. VIII. IX. XI. XIII. XIV. XV.
*Primulaveris* L.	ntv	II. XII.
*Primulavulgaris* Huds.	ntv	XI.
*Prunellavulgaris* L.	ntv	II. III. IV. VIII. XI. XII. XIII.
*Prunusarmeniaca* L.	arc	XI.
*Prunusavium* (L.) L.	ntv	II. III. IX. XI. XII. XXI. XXII.
*Prunuscerasifera* Ehrh.	arc	I. II. III. IV. V. VI. IX. X. XI. XII. XIII. XIV. XV. XVI. XVII. XX. XXI.
*Prunuscerasus* L.	arc	I. III. IV. VIII. XI. XII. XXI. XXIII.
*Prunusdomestica* L.	arc	III. VI. XII. XVII.
*Prunuslaurocerasus* L.	neo	XV.
*Prunusmahaleb* L.	ntv/arc	I. VI. IX. X. XI. XII.
*Prunuspadus* L.	ntv	IV. IX. XII. XV.
*Prunuspersica* (L.) Batsch	arc	IV. VIII.
*Prunusserotina* Ehrh.	neo	IV. XI.
*Prunusspinosa* L.	ntv	I. II. III. X. XI. XII. XXII.
*Prunustenella* Batsch	ntv	III. IV.
*Pteridiumaquilinum* (L.) Kuhn	ntv	III. IX.
*Pterismultifida* Poir.	neo	IX. XII.
*Puccinelliadistans* (Jacq.) Parl.	ntv	III. XXI.
*Pulicariadysenterica* (L.) Bernh.	ntv	III. IX.
*Pulmonariaofficinalis* L.	ntv	III.
*Pyracanthacoccinea* M.Roem.	neo	I. III. IV. VIII. IX. XI. XII.
*Pyruspyraster* (L.) Burgsd.	ntv	XII.
*Quercuscerris* L.	ntv	II. III. XI. XII.
*Quercuspetraea* (Matt.) Liebl.	ntv	III. IX. XI. XII.
*Quercuspubescens* Willd.	ntv	III.
*Quercusrobur* L.	ntv	II. III. IV. XIII. XIV. XXII.
*Ranunculusacris* L.	ntv	III.
*Ranunculusbulbosus* L.	ntv	I. II. X. XI. XIV.
*Ranunculusficaria* L.	ntv	I. IV. XI. XII. XV. XVI.
*Ranunculuslanuginosus* L.	ntv	III.
*Ranunculuspedatus* Waldst. & Kit.	ntv	IX.
*Ranunculuspolyanthemos* L.	ntv	I. II. III. IV. XII.
*Ranunculusrepens* L.	ntv	I. III. V. VIII. IX. X. XI. XIV. XVII. XXII. XXIII.
*Ranunculussardous* Crantz	arc	I. IX. XIII. XX.
*Ranunculussceleratus* L.	ntv	IV. XI. XIII. XX.
*Raphanusraphanistrum* L.	arc	XI. XIII. XIV.
Raphanusraphanistrumsubsp.sativus (L.) Domin	arc	V.
*Rapistrumperenne* (L.) All.	arc	XXIII.
*Resedalutea* L.	arc	I. II. III. IV. V. VI. VIII. IX. X. XI. XII. XIII. XIV. XV. XVI. XX. XXI. XXII. XXIII.
*Resedaphyteuma* L.	arc	IX. XI.
*Reynoutriabohemica* Chrtek & Chrtková	neo	I. II. III. IV. VI. VIII. IX. X. XI. XII. XIII. XIV. XVI. XX. XXI. XXII. XXIII.
*Rhamnuscathartica* L.	ntv	XII.
*Rhustyphina* L.	neo	II. III. IX. XI. XIII. XIV. XX. XXI.
*Ribesaureum* Pursh	neo	XV. XVI.
*Robiniapseudoacacia* L.	neo	I. II. III. IV. V. VI. VII. VIII. IX. X. XI. XII. XIII. XIV. XV. XVI. XVII. XVIII. XIX. XX. XXI. XXII. XXIII.
*Robiniaviscosa* Michx. ex Vent.	neo	XI. XIX. XX.
*Rorippaaustriaca* (Crantz) Besser	ntv	XIII. XX.
*Rorippapalustris* Besser	ntv	XIII. XIV. XXI.
*Rorippasylvestris* (L.) Besser	ntv	I. II. V. VI. VIII. IX. XI. XIII. XIV. XX. XXI.
*Rosacanina* L.	ntv	I. II. III. VI. VIII. IX. X. XI. XII. XIV. XV. XVI. XVII. XIX. XXI. XXII. XXIII.
*Rosarubiginosa* L.	ntv	XII.
*Rubuscaesius* L.	ntv	III. IV. VIII. IX. XI. XIII. XXI. XXIII.
*Rubusidaeus* L.	ntv	XIII.
*Rubuspraecox* Bertol.	ntv	IX.
*Rudbeckiahirta* L.	neo	I.
*Rumexacetosa* L.	ntv	IV. XV.
*Rumexacetosella* L.	ntv	VIII. IX. XIII. XIV. XXIII.
*Rumexconglomeratus* Murray	ntv	VII. XIV.
*Rumexcrispus* L.	arc	I. II. III. IV. V. VI. VIII. IX. X. XI. XII. XIII. XIV. XV. XVI. XVII. XX. XXI. XXII. XXIII.
*Rumexobtusifolius* L.	ntv	I. III. XI. XIX. XXII.
*Rumexpalustris* Sm.	ntv	IX.
*Rumexpatientia* L.	arc	III. IV. VII. VIII. IX. XI. XXIII.
*Rumexsanguineus* L.	ntv	II.
*Rumexthyrsiflorus* Fingerh.	ntv	III. VII. IX. X. XII. XIV. XVI.
*Ruscusaculeatus* L.	ntv	XI. XII. XIII.
*Rutagraveolens* L.	neo	XVI.
*Saginaapetala* Ard.	ntv	I. II. VI. VII. VIII. IX. XI. XIV.
*Saginamicropetala* Rauschert	ntv	III.
*Saginaprocumbens* L.	arc	I. II. III. IV. V. VI. VII. VIII. IX. XI. XII. XIII. XIV. XV. XVI. XX. XXI.
*Salixalba* L.	ntv	III. IV. V. VI. VIII. IX. XI. XIII.
*Salixbabylonica* L.	neo	IX. XXI.
*Salixcaprea* L.	ntv	I. IX.
*Salixcinerea* L.	ntv	III.
*Salixfragilis* L.	ntv	XXIII.
*Salixpurpurea* L.	ntv	IV.
*Salsolakali* L.	ntv	IV. XIV. XXI. XXIII.
*Salvianemorosa* L.	ntv	II. III. IV. VIII. IX. XI. XIV.
*Salviaofficinalis* L.	arc/neo	XII.
*Salviapratensis* L.	ntv	IV. VIII. IX. XI. XII.
*Salviasclarea* L.	arc/neo	II. III. XXII.
*Sambucusebulus* L.	ntv	III. XI. XII. XXI.
*Sambucusnigra* L.	ntv	I. II. III. IV. VI. VIII. IX. X. XI. XII. XIII. XIV. XV. XVI. XVII. XIX. XX. XXI. XXII.
*Sanguisorbaminor* Scop.	ntv	II. III. IV. VIII. XI. XII. XIV. XX. XXII.
*Saponariaocymoides* L.	neo	III.
*Saponariaofficinalis* L.	arc	VIII. IX. XI. XXI. XXIII.
*Saturejahortensis* L.	arc	III. VIII. XV. XVI.
*Saxifragatridactylites* L.	ntv	VIII.
*Scabiosaochroleuca* L.	ntv	I. III. IV. IX. X. XI. XII. XXI. XXIII.
*Schoenoplectuslacustris* (L.) Palla	ntv	XXIII.
*Scillavindobonensis* Speta	ntv	XI. XV.
*Scleranthusannuus* L.	ntv	XIV.
*Sclerochloadura* (L.) P.Beauv.	ntv/arc	I. II. III. VI. XI. XII. XIII. XIV. XV. XVI.
*Scorzoneroidesautumnalis* (L.) Moench	ntv	IV.
*Secalecereale* L.	arc	III. XI.
*Secalesylvestre* Host	ntv/arc	XV.
*Sedumacre* L.	ntv	II. III. IV. VIII. IX. X. XI. XII. XIII. XV. XX. XXI.
*Sedumalbum* L.	ntv	III. IV. VI. IX. XI. XIV. XV. XVI. XXII. XXIII.
*Sedumhispanicum* L.	ntv	III. IV. VIII. IX. X. XI. XII. XIII. XVI. XIX. XX. XXII.
*Sedumsarmentosum* Bunge	neo	III. IX. XII. XIII. XIV. XV.
*Sedumsexangulare* L.	ntv	II. III. IX. XI. XIII. XV. XXIII.
*Senecioinaequidens* DC.	neo	IV. VIII. IX. X. XI. XIV.
*Seneciovernalis* Waldst. & Kit.	neo	I. II. III. VI. VII. VIII. IX. X. XI. XII. XIII. XIV. XV. XVI. XX. XXI.
*Seneciovulgaris* L.	arc	I. II. III. IV. V. VI. VII. VIII. IX. X. XI. XII. XIII. XIV. XV. XVI. XVII. XVIII. XIX. XX. XXI. XXII. XXIII.
*Serratulatinctoria* L.	ntv	III.
*Seseliannuum* L.	ntv	XI.
*Seseliosseum* Crantz	ntv	I. XII.
*Seselipallasii* Besser	ntv	XXIII.
*Setariaitalica* (L.) P.Beauv.	arc	XI.
*Setariapumila* (Poir.) Roem. & Schult.	arc	II. III. IV. VIII. IX. X. XI. XII. XIII. XIV. XV. XVI. XVII. XX. XXI. XXII. XXIII.
*Setariaverticillata* (L.) P.Beauv.	arc	I. II. III. IV. V. VIII. IX. X. XI. XII. XIII. XIV. XV. XVI. XVII. XIX. XX. XXI. XXII.
*Setariaviridis* (L.) P.Beauv.	arc	I. II. III. IV. V. VI. VII. VIII. IX. X. XI. XII. XIII. XIV. XV. XVI. XVII. XVIII. XIX. XX. XXI. XXII. XXIII.
Setariaviridissubsp.pycnocoma (Steud.) Tzvelev	neo	XV.
*Sherardiaarvensis* L.	arc	XIV.
*Sileneconica* L.	ntv	XXI.
*Silenecoronaria* (L.) Clairv.	ntv	II.
Silenelatifoliasubsp.alba (Mill.) Greuter & Burdet	ntv	I. II. III. IV. V. VI. VII. VIII. IX. X. XI. XII. XIII. XIV. XV. XVI. XVII. XVIII. XIX. XX. XXI. XXII. XXIII.
*Silenenoctiflora* L.	ntv	XII.
*Silenenutans* L.	ntv	XI. XII.
*Sileneotites* (L.) Wibel	ntv	IX.
*Silenevulgaris* (Moench) Garcke	ntv	II. III. IV. VI. VIII. IX. XI. XII. XIII. XXIII.
*Silybummarianum* (L.) Gaertn.	neo	III.
*Sinapisalba* L.	arc	II. VIII. XI. XIII.
*Sinapisarvensis* L.	arc	V. VI. VIII. XII. XIV. XVI.
*Sisymbriumaltissimum* L.	arc	VIII. XI. XIII. XIV.
*Sisymbriumirio* L.	neo	XIII.
*Sisymbriumloeselii* L.	arc	I. II. IV. VI. VIII. IX. X. XI. XII. XIII. XIV. XV. XIX. XX. XXI. XXIII.
*Sisymbriumofficinale* (L.) Scop.	ntv/arc	III.
*Sisymbriumorientale* L.	arc	I. II. IV. VI. VII. VIII. IX. X. XI. XIV. XV.
*Sisymbriumstrictissimum* L.	ntv	X.
*Smyrniumperfoliatum* L.	ntv	I. XII.
*Solanumdulcamara* L.	ntv	I. II. III. VII. VIII. IX. X. XI. XII. XIII. XX. XXI. XXII.
*Solanumlycopersicum* L.	neo	I. II. III. IV. V. VI. VII. VIII. IX. X. XII. XIII. XIV. XVI. XIX. XX. XXI. XXII. XXIII.
*Solanumnigrum* L.	arc	I. II. III. IV. V. VI. VII. VIII. IX. X. XI. XII. XIII. XIV. XV. XVI. XVII. XVIII. XX. XXI. XXII. XXIII.
*Solanumtuberosum* L.	neo	IX.
*Solanumvillosum* Mill.	neo	IV. IX.
*Solidagocanadensis* L.	neo	I. II. III. IV. V. VI. VIII. IX. X. XI. XII. XIII. XIV. XV. XVI. XVII. XVIII. XIX. XX. XXI. XXII. XXIII.
*Solidagogigantea* Aiton	neo	II. III. IV. V. VII. VIII. IX. X. XI. XII. XIII. XIV. XV. XVI. XXI. XXII.
*Solidagovirgaurea* L.	ntv	XII.
*Sonchusarvensis* L.	arc	I. III. IV. VI. VIII. IX. XI. XII. XIII. XIV. XVI. XXI. XXIII.
*Sonchusasper* (L.) Hill	arc	I. II. VI. VIII. IX. XII. XIII. XIV. XV. XX. XXI.
*Sonchusoleraceus* L.	arc	I. II. III. IV. V. VI. VII. VIII. IX. X. XI. XII. XIII. XIV. XV. XVI. XVII. XVIII. XIX. XX. XXI. XXII. XXIII.
*Sophorajaponica* L.	neo	IV. V. VI. VIII. IX. XIII. XV. XXII.
*Sorbariasorbifolia* (L.) A.Braun	neo	II.
*Sorbusaucuparia* L.	ntv	IX.
*Sorbussemiincisa* Borbás	ntv	II. XI.
*Sorghumhalepense* (L.) Pers.	neo	I. II. III. IV. V. VIII. IX. X. XI. XIII. XIV. XVII. XIX. XX. XXI. XXII. XXIII.
*Spergulariarubra* J.Presl & C.Presl	ntv	V.
*Spiraeachamaedryfolia* L.	neo	IV.
*Spiraeajaponica* L.f.	neo	XIII.
*Spiraeavanhouttei* (Briot) Zabel	neo	VIII. XI. XII.
*Spirodelapolyrhiza* (L.) Schleid.	ntv	XX.
*Stachysannua* L.	arc	II. III. VIII. XIII. XV. XXII.
*Stachysbyzantina* K.Koch	neo	XXII.
*Stachyspalustris* L.	ntv	XIV.
*Stachysrecta* L.	ntv	I. XI. XII.
*Stachyssylvatica* L.	ntv	XII.
*Stellariaaquatica* Scop.	ntv	I. III. V. XVI. XVII. XXII.
*Stellariaholostea* L.	ntv	I.
*Stellariamedia* (L.) Vill.	ntv	I. II. III. IV. V. VI. VII. VIII. IX. X. XI. XII. XIII. XIV. XV. XVI. XVIII. XIX. XX. XXI. XXII. XXIII.
*Stellariapallida* (Dumort.) Crép.	ntv	I. II. III. IV. V. VI. VII. XI. XIV. XVI.
*Symphoricarposalbus* (L.) S.F.Blake	neo	I. II. III. IX. XI. XII. XIV. XV.
*Symphoricarposorbiculatus* Moench	neo	XI. XIII. XXII.
*Symphyotrichumlanceolatum* (Willd.) G.L.Nesom	neo	I. III. IV. V. VI. VIII. IX. X. XI. XII. XIII. XX. XXI. XXIII.
*Symphyotrichumnovi-belgii* (L.) G.L.Nesom	neo	III.
*Symphyotrichumtradescantii* (L.) G.L.Nesom	neo	III.
*Symphytumofficinale* L.	ntv	III. IX. XI. XIV. XV. XX. XXIII.
*Syringavulgaris* L.	neo	I. II. III. IV. V. VI. VIII. XI. XII. XIII. XIV. XV. XVI. XVII. XIX. XX. XXII.
*Tagetespatula* L.	neo	I. III. XIX. XXII.
*Talinumpaniculatum* (Jacq.) Gaertn.	neo	XI.
*Tamarixtetrandra* Pall. ex M.Bieb.	neo	III. XI. XV. XXI.
*Tanacetumcorymbosum* (L.) Sch.Bip.	ntv	XI.
*Tanacetumparthenium* (L.) Sch.Bip.	neo	III. VII. VIII. XI. XV. XIX.
*Tanacetumvulgare* L.	ntv/arc	V. IX. X. XIV.
*Taraxacumerythrospermum* Andrz. ex Besser	ntv	XI.
*Taraxacumofficinale* F.H.Wigg.	ntv	I. II. III. IV. V. VI. VII. VIII. IX. X. XI. XII. XIII. XIV. XV. XVI. XVII. XVIII. XIX. XX. XXI. XXII. XXIII.
*Taxusbaccata* L.	ntv	I. II. III. IV. V. VIII. IX. XI. XII.
*Teucriumchamaedrys* L.	ntv	XI. XIII. XX.
*Teucriumscordium* L.	ntv	VI.
*Thalictrumflavum* L.	ntv	V.
*Thelypterispalustris* Schott	ntv	V. VI. VIII. IX. X. XII. XIII.
*Thesiumramosum* Hayne	ntv	VI. XXII.
*Thladianthadubia* Bunge	neo	I. VIII.
*Thlaspiarvense* L.	ntv/arc	XI.
*Thlaspiperfoliatum* L.	ntv	I. III. XVI.
*Thujaoccidentalis* L.	neo	XV.
*Thymusodoratissimus* Mill.	ntv	IV. IX.
Thymuspulegioidessubsp.pannonicus (All.) Kerguélen	ntv	XV.
*Tiliacordata* Mill.	ntv	III. IX.
*Tiliaplatyphyllos* Scop.	ntv	I. II. III. X. XI. XII. XIII. XV. XVI.
*Tiliatomentosa* Moench	ntv	I. XIII. XV.
*Tordyliummaximum* L.	ntv/arc	XIV.
*Torilisarvensis* (Huds.) Link	arc	II. III. V. VIII. IX. X. XI. XII. XIII. XIV. XVII. XXII.
*Torilisjaponica* DC.	ntv	III. XI. XIII.
*Tradescantiavirginiana* L.	neo	III. XIII. XV. XIX. XX.
*Tragopogondubius* Scop.	ntv	I. II. VI. VIII. XI. XII. XIII. XIV. XV. XVI. XX. XXI.
*Tragopogonorientalis* L.	ntv	I. III. IV. V. VIII. IX. X. XI. XIII. XIV. XV. XVI. XVII. XX. XXI. XXII. XXIII.
*Tragusracemosus* (L.) All.	neo	III. IV. V. VIII. IX. X. XI. XIII. XV. XVI. XIX. XX. XXI. XXIII.
*Tribulusterrestris* L.	ntv/arc	II. III. IV. VI. VIII. IX. X. XI. XIII. XIV. XV. XIX. XX. XXI.
*Trifoliumarvense* L.	ntv	I. II. XV. XVI.
*Trifoliumcampestre* Schreb.	ntv	II. XI. XIV. XV.
*Trifoliumfragiferum* L.	ntv	XI. XX.
*Trifoliumincarnatum* L.	neo	XXIII.
*Trifoliummedium* L.	ntv	XII.
*Trifoliumpratense* L.	ntv	I. II. III. IV. VI. VIII. IX. X. XI. XII. XIII. XIV. XV. XVII. XX. XXI. XXIII.
*Trifoliumrepens* L.	ntv	I. II. III. IV. V. VI. VII. VIII. IX. X. XI. XII. XIII. XIV. XV. XVI. XVII. XVIII. XIX. XX. XXI. XXII. XXIII.
*Tripidiumravennae* (L.) H.Scholz	neo	X.
*Tripleurospermuminodorum* (L.) Sch.Bip.	arc	II. III. V. VI. VIII. IX. X. XI. XIII. XIV. XV. XX.
*Triticumaestivum* L.	arc	II. III. VIII. XII. XIV. XVI. XXI.
*Tulipagesneriana* L.	neo	IX.
*Tussilagofarfara* L.	ntv	II. III. VIII. IX. XII. XIII. XIV.
*Typhalatifolia* L.	ntv	II. VII. XXIII.
*Ulmuslaevis* Pall.	ntv	I. II. III. IX. XI. XII. XIII.
*Ulmusminor* Mill.	ntv	I. II. III. IV. V. VI. VII. VIII. IX. X. XI. XII. XIII. XIV. XV. XVI. XVII. XVIII. XIX. XX. XXI. XXII.
*Ulmuspumila* L.	neo	I. II. IV. V. VI. VII. VIII. IX. X. XI. XIII. XIV. XX. XXI. XXIII.
*Urticadioica* L.	ntv	I. II. III. IV. V. VIII. IX. X. XI. XII. XIII. XIV. XV. XVI. XVII. XX. XXI. XXII. XXIII.
*Urticaurens* L.	arc	I. V. XIII.
*Vaccariahispanica* (Mill.) Rauschert	arc	XIII.
*Valerianaofficinalis* L.	ntv	III.
*Valerianellacarinata* Loisel.	ntv/arc	XI. XIV. XX.
*Valerianellalocusta* (L.) Laterr.	arc	I. III. VI. VII. VIII. XIV. XV. XVI.
*Verbascumblattaria* L.	ntv	IV. X. XIII. XIV. XXIII.
Verbascumchaixiisubsp.austriacum (Schott ex Roem. & Schult.) Hayek	ntv	XII.
*Verbascumdensiflorum* Bertol.	ntv	XVIII.
*Verbascumlychnitis* L.	ntv	VI. X. XI. XV. XXIII.
*Verbascumphlomoides* L.	ntv	II. III. IV. V. VIII. IX. X. XI. XII. XIII. XIV. XV. XVI. XVII. XIX. XX. XXI. XXIII.
*Verbascumphoeniceum* L.	ntv	VI. IX.
*Verbenabonariensis* L.	neo	I. II. III. V. VIII. XIII.
*Verbenaofficinalis* L.	arc	I. II. III. IV. V. VI. VII. VIII. IX. X. XI. XII. XIII. XIV. XV. XVI. XVII. XVIII. XX. XXI. XXII. XXIII.
*Veronicaanagallis-aquatica* L.	ntv	II. III. XVII.
*Veronicaarvensis* L.	arc	I. II. III. V. VI. VII. VIII. IX. X. XI. XII. XIII. XIV. XV. XVI. XX.
*Veronicaaustriaca* L.	ntv	XI.
*Veronicacatenata* Pennell	ntv	XVI.
*Veronicahederifolia* L.	ntv/arc	I. II. III. IV. V. VI. VII. VIII. IX. X. XI. XII. XIII. XIV. XV. XVI.
*Veronicaperegrina* L.	neo	III. XIV.
*Veronicapersica* Poir.	neo	I. II. III. V. VI. VII. VIII. IX. XI. XII. XIII. XIV. XV. XVI. XXI.
*Veronicapolita* Fr.	arc	I. II. III. V. VI. VII. IX. XI. XII. XIV. XV. XVI. XX.
*Veronicapraecox* All.	ntv	XV.
*Veronicaprostrata* L.	ntv	X.
*Veronicaserpyllifolia* L.	ntv	XI.
*Veronicasublobata* M.A.Fisch.	arc/neo	I. III. XII.
*Veronicatriloba* (Opiz) Opiz	ntv/arc	III.
*Veronicatriphyllos* L.	arc	XV.
*Viburnumlantana* L.	ntv	III. VI. X. XII. XV.
*Viburnumopulus* L.	ntv	II. VIII.
*Viburnumrhytidophyllum* Hemsl.	neo	VIII. XII.
*Viciacracca* L.	arc	III. X. XI. XII. XXIII.
*Viciagrandiflora* Scop.	ntv/arc	III. VI. VII. XIII. XV. XVI.
*Viciahirsuta* (L.) Gray	arc	I. III. VIII. XI. XIII. XIV. XV. XVI. XX.
*Vicialathyroides* L.	ntv	I. IV. IX. X. XI.
*Viciasativa* L.	arc	I. II. III. V. VI. VII. VIII. XI. XII. XIII. XIV. XV. XX.
Viciasativasubsp.nigra (L.) Ehrh.	ntv/arc	IX.
*Viciasepium* L.	ntv	XII. XXIII.
*Viciatenuifolia* Roth	ntv/arc	III. XXI.
*Viciatetrasperma* (L.) Schreb.	arc	II. IV. VIII. IX. X. XI. XIV.
*Viciavillosa* Roth	arc	II. IV. VI. VIII. IX. X. XII. XIII. XIV. XX.
*Vincamajor* L.	neo	I. III. XI. XII. XIV. XV. XVI.
*Vincaminor* L.	ntv	VIII. XI. XII. XV. XVI.
*Vincetoxicumhirundinaria* Medik.	ntv	XI.
*Violaarvensis* Murray	arc	I. II. III. V. VII. VIII. IX. X. XI. XIII. XV. XX.
*Violahirta* L.	ntv	IX. XV.
*Violaodorata* L.	arc	I. II. III. IV. V. VI. VII. VIII. IX. X. XI. XII. XIII. XIV. XV. XVI. XVIII. XIX. XX. XXI. XXII. XXIII.
*Violareichenbachiana* Jord. ex Boreau	ntv	I. III. IX. XI. XII. XV.
*Violasororia* Willd.	neo	I. III. IV. VIII. IX. XI. XII. XIII. XIV. XV. XVI. XVII. XIX.
*Violatricolor* L.	ntv	II. III. IV. VIII. XII. XIV. XV. XVI.
*Violawittrockiana* Gams	neo	XIII.
*Viscumalbum* L.	ntv	III. XI. XII.
*Vitexagnus-castus* L.	neo	VIII. IX.
*Vitisvinifera* L.	arc	I. III. XI. XIII. XIV. XV. XVII. XIX. XXI. XXII.
*Vulpiamyuros* (L.) C.C.Gmel.	ntv/arc	I. II. III. VI. VIII. IX. X. XI. XIII. XIV. XV. XX. XXIII.
*Wisteriasinensis* (Sims) DC.	neo	I. X. XI.
*Xanthiumsaccharatum* Wallr. & Widder	neo	XI.
*Xanthiumstrumarium* L.	arc	XXI.
*Yuccafilamentosa* L.	neo	XIX.
*Zeamays* L.	neo	XIII. XX. XXI.
*Zinniaelegans* L.	neo	XIII.
